# Comparative Study of the Synthetic Approaches and Biological Activities of the Bioisosteres of 1,3,4-Oxadiazoles and 1,3,4-Thiadiazoles over the Past Decade

**DOI:** 10.3390/molecules27092709

**Published:** 2022-04-22

**Authors:** Rana M. El-Masry, Hanan H. Kadry, Azza T. Taher, Sahar M. Abou-Seri

**Affiliations:** 1 Organic Chemistry Department, Faculty of Pharmacy, October University for Modern Sciences and Arts (MSA), 6th of October City 12451, Egypt; 2 Department of Pharmaceutical Organic Chemistry, Faculty of Pharmacy, Cairo University, Cairo 11562, Egypt; hanan.kadry@pharma.cu.edu.eg (H.H.K.); azza.shalaby@pharma.cu.edu.eg (A.T.T.); 3 Department of Organic Pharmaceutical Chemistry, Faculty of Pharmacy, October 6 University (O6U), 6th of October City 12585, Egypt; 4 Department of Pharmaceutical Chemistry, Faculty of Pharmacy, Cairo University, Cairo 11562, Egypt

**Keywords:** 1,3,4-oxadiazole, 1,3,4-thiadiazole, bioisosteres, synthesis, biological activity, structure–activity relationship

## Abstract

The bioisosteres of 1,3,4-oxadiazoles and 1,3,4-thiadiazoles are well-known pharmacophores for many medicinally important drugs. Throughout the past 10 years, 1,3,4-oxa-/thiadiazole nuclei have been very attractive to researchers for drug design, synthesis, and the study of their potential activity towards a variety of diseases, including microbial and viral infections, cancer, diabetes, pain, and inflammation. This work is an up-to-date comparative study that identifies the differences between 1,3,4-thiadiazoles and 1,3,4-oxadiazoles concerning their methods of synthesis from different classes of starting compounds under various reaction conditions, as well as their biological activities and structure–activity relationship.

## 1. Introduction

The nitrogen-containing five-membered heterocycles, particularly 1,3,4-oxadiazoles and 1,3,4-thiadiazoles, have received considerable attention because of their versatile biological properties and applications in drug discovery [1,2]. Based on bioisosterism between sulfur and oxygen atoms [3], 2-amino-1,3,4-thiadiazole could act as a bioisostere of 2-amino-1,3,4-oxadiazole and, therefore, produce biological properties broadly similar to those of the latter [4]. Moreover, these moieties are used as pharmacophores due to their favorable metabolic profile and ability to form hydrogen bonds [5,6].

Different substituted 1,3,4-oxadiazole molecules possess antimicrobial [7,8], antitubercular [9], antimalarial [10], analgesic [11], anticonvulsant [12], anti-inflammatory [13], antihepatitis B virus [14], anti-severe acute respiratory syndrome coronavirus 2 (anti-SARS-CoV-2) [15], anticancer [10,16], and antioxidant activities [17]. The antioxidants prevent the onset and propagation of oxidative disorders like autoimmune, cardiovascular, neurovascular diseases [2].

**Figure 1:**
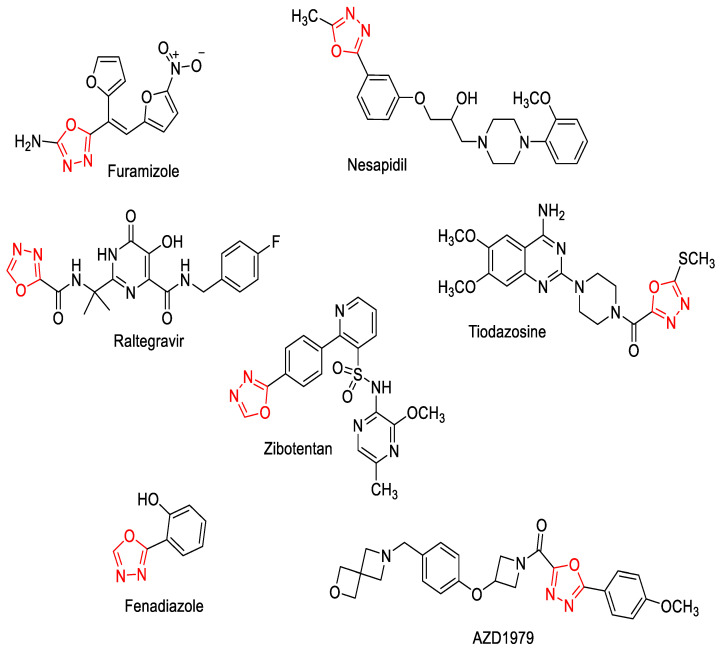
Structures of biologically active 1,3,4-oxadiazoles.

The potent pharmacological activity of 1,3,4-oxadiazoles can be attributed to the presence of the toxophoric –N=C–O– linkage, which may react with the nucleophilic centers of the cell [18,19]. Some of the drugs that contain an oxadiazole nucleus (Figure 1) are Furamizole, a nitrofuran derivative with a strong antibacterial activity [20,21]; Nesapidil, a well-established vasodilating agent [22]; Raltegravir, also known as MK-0518, which was the first FDA-approved HIV-1 integrase (IN) inhibitor launched by Merck & Co to treat HIV infection [23]; Tiodazosin, an α1-adrenergic receptor antagonist used to treat hypertension [24,25]; Fenadiazole, a hypnotic drug [26,27]; Zibotentan, an anticancer agent [28,29]; and AZD1979, a melanin-concentrating hormone receptor 1 (MCHr1) antagonist [30].

**Figure 2:**
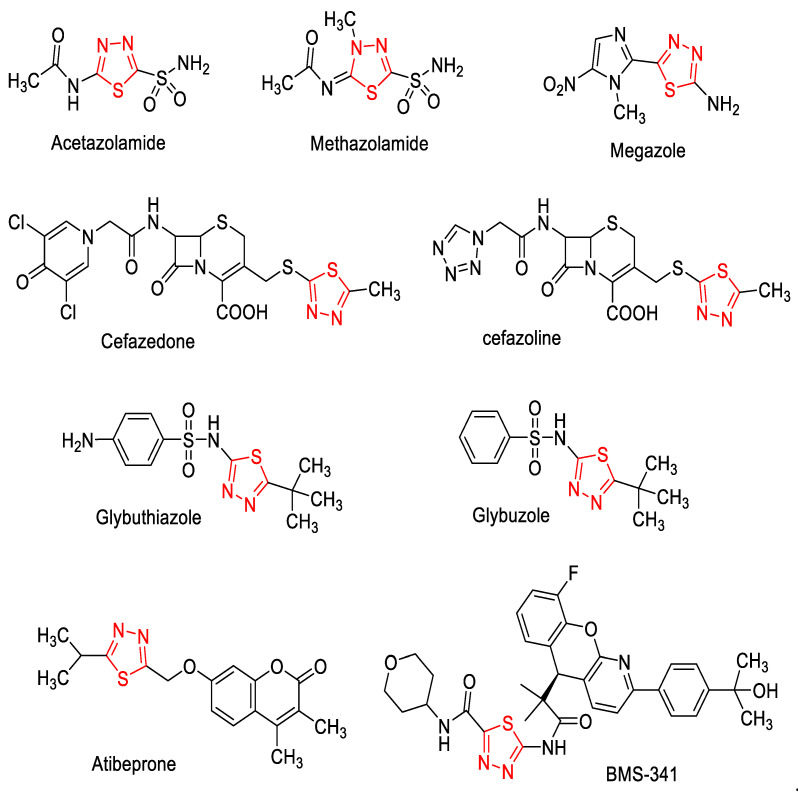
Structures of biologically active 1,3,4-thiadiazoles.

Compared to 1,3,4-oxadiazole, the sulfur atom of 1,3,4-thiadiazole imparts improved lipid solubility, and the mesoionic nature of 1,3,4-thiadiazole makes this class of compounds show good tissue permeability [6]. Thus, the 1,3,4-thiadiazole fragment appears in many clinically used drugs (Figure 2), such as the diuretic drugs Acetazolamide and Methazolamide; Cefazolin CFZL and Cefazedone CFZD, which are first-generation cephalosporins; Atibeprone, an anti-depressant drug; antidiabetic agents Glybuthiazole and Glybuzole; antiprotozoal drug Megazol [31]; and BMS341, a glucocorticoid receptor modulator [32]. Moreover, 1,3,4-thiadiazole derivatives are reported to possess anticancer [33], antioxidant [34], antimicrobial [35], antidepressant [36], anticonvulsant [37], analgesic, and anti-inflammatory activities [38].

**Figure 3:**
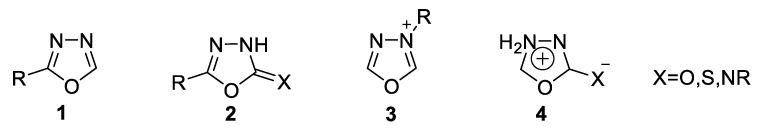
Aromatic systems of 1,3,4-oxadiazole.

At the molecular level, 1,3,4-oxadiazole **1** is a thermally stable neutral aromatic molecule. It has four possible aromatic systems, **1–4**: 1,3,4-oxadiazole **1**, which is widely exploited for various applications; 1,3,4-oxadiazolines **2**; 1,3,4-oxadiazolium cations **3**; and the exocyclic-conjugated mesoionic 1,3,4-oxadiazole **4** (Figure 3) [39]. 

**Figure 4:**
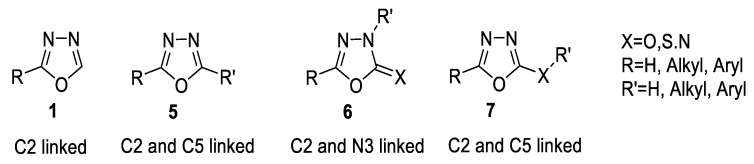
Positions of substitution at the 1,3,4-oxadiazole ring.

The linked substituents to 1,3,4-oxadiazole aromatic moieties normally appearing at positions C2, C5, and N3 may be represented by the following structural features **1**,**5–7** (Figure 4) [39].

The electronic distribution in 1,3,4-oxadiazole has been calculated by various Self-consistent Field Molecular Orbital (SCF-MO) methods [40]. Other structural parameters, the dipole moment, and data on its ultraviolet–visible (λ max calculated to be in the region 193–203 nm), NMR, NQR, and microwave spectra have been derived. Studies on 1,3,4-oxadiazole indicate a maximum positive charge at the 2-position [41]. Molecular diagrams for 1,3,4-oxadiazole, 2- phenyl- and 2,5-diphenyl-1,3,4-oxadiazole, and oligomeric oxadiazoles have been derived, and conjugation between the rings was found to be similar to that in polyphenyls [42]. 

**Figure 5:**
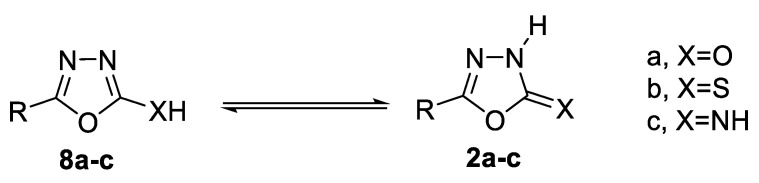
Tautomerism of 1,3,4-oxadiazole.

Tautomerism in 2-hydroxy **8a**, 2-mercapto **8b**, and 2-amino-1,3,4-oxadiazole **8c** is in equilibrium with the tautomeric oxadiazolines **2a**, **2b**, and **2c**, respectively (Figure 5) [43]. Evidence from UV, IR, ^1^H-NMR, and ^13^C-NMR spectra supports structure **2b** for 1,3,4-oxadiazoline-5-thione [44]. 

The development of 1,3,4-thiadiazole chemistry is linked to the discovery of phenylhydrazines and hydrazine in the late 19th century. The first 1,3,4-thiadiazole was described by Fischer in 1882 and further developed by Bush and coworkers [45], but the true nature of the ring system was demonstrated first in 1956 by Goerdler et al. [46].

**Figure 6:**
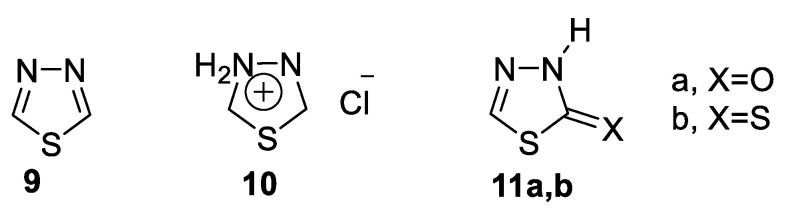
1,3,4-thiadiazole ring systems.

Similar to 1,3,4-oxadiazoles, the 1,3,4-thiadiazole derivatives are conveniently divided into three subclasses (Figure 6):

- Aromatic systems that include the neutral thiadiazoles **9** [45].
- Mesoionic systems that are defined as a poly-heteroatomic system containing a five-membered heterocyclic ring associated with conjugated p and π electrons and distinct regions of positive and negative charges **10**. Mesoionic systems are dense and highly polarizable, with a net neutral electron charge; these characteristics allow mesoionic compounds to cross cellular membranes and interact with biological targets with distinct affinities [31].
- 1,3,4-thiadiazol-2-one(thione) **11a,b** [47].

**Figure 7:**
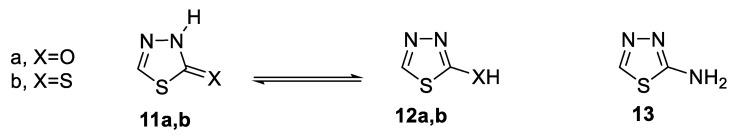
Tautomeric forms of 2-substituted 1,3,4-thiadiazoles.

Further, the 1,3,4-thiadiazole ring system, with three heteroatoms, does not exhibit tautomerism in its fully conjugated form **9**. However, when certain substituents are present, tautomerism is possible. 1,3,4-Thiadiazolin-2-ones **11a** and -2-thione **11b** exist in the oxo **12a** and thione **12b** forms, respectively [47]. 2-Amino-1,3,4-thiadiazoles exist in the amino form **13** in solution and in the solid state (Figure 7) [48].

**Figure 8:**
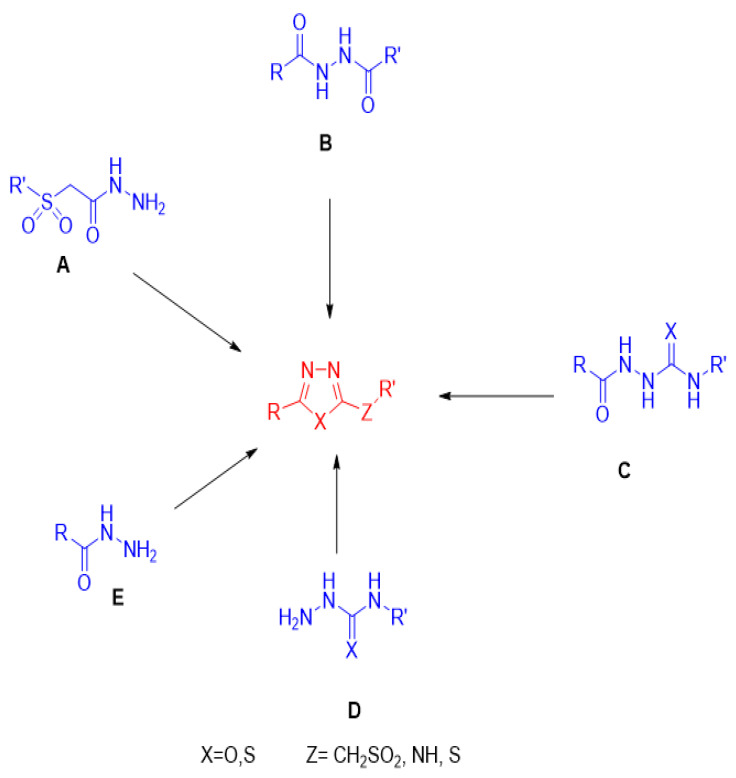
Synthetic routes for the synthesis of different classes 1,3,4-oxadiazoles and 1,3,4-thiadiazoles from diverse starting materials. (**A**): Sulphonyl acetic acid hydrazide; (**B**): 1,2-diacyl hydrazine; (**C**): Acyl semi/thiosemicarbazide; (**D**): Semi/thiosemicarbazide; (**E**): Acid hydrazide.

Various synthetic routes for the synthesis of different classes of 1,3,4-oxadiazoles and 1,3,4-thiadiazoles from diverse starting materials are illustrated in this collective chart (Figure 8) and are discussed in detail in this review. The biological activities of different 1,3,4-oxadiazole and 1,3,4-thiadiazole classes are also summarized herein.

## 2. Synthetic Approaches for the Preparation of Different Classes of 1,3,4-Oxadiazole and 1,3,4-Thiadiazole Derivatives

The synthetic approaches adopted for the preparation of 1,3,4-oxadiazole and 1,3,4-thiadiazole derivatives can be classified according to the starting material as follows: 

### 2.1. Synthesis from Sulfonyl Acetic Acid Hydrazide

**Figure 9:**
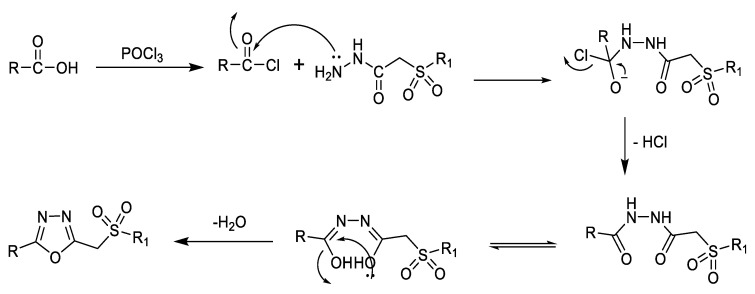
Proposed mechanism for the synthesis of 1,3,4-oxadiazole derivatives using sulfonyl acetic acid hydrazide based on the reported mechanism of formation of oxadiazole from hydrazide acid and carboxylic acid [49].

Sulphonylmethyl-1,3,4-oxadiazole derivatives are synthesized by cyclocondensation of sulfonyl acetic acid hydrazide with derivatives of carboxylic acids or sulfonyl acetic acids. The most common reagent used for cyclocondensation is POCl_3_ (Figure 9). The 1,3,4-oxadiazole can then be interconverted to the 1,3,4-thiadiazole analog using thiourea and tetrahydrofuran (THF) [6].

**Scheme 1:**
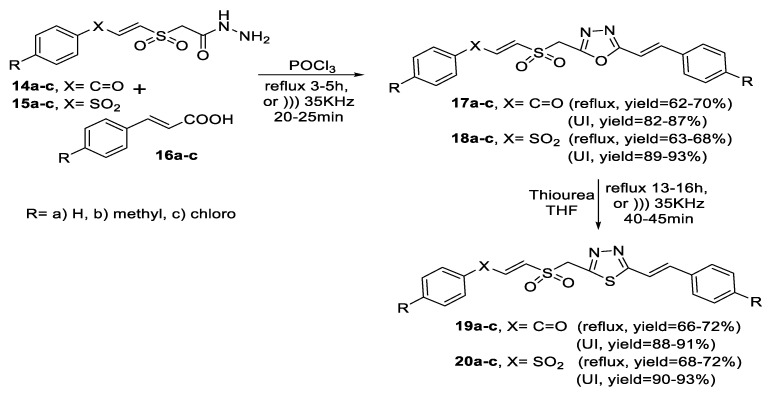
Synthetic pathway for compounds **17a–c**–**20a–c**.

In 2019, Sekhar et al. [6] reported the cyclocondensation of *E*-aroylethene (**14a–c**) or *E*-arylsulfonylethene (**15a–c**) sulfonylacetic acid hydrazides with substituted cinnamic acid (**16a–c**) using the conventional method by reflux in POCl_3_ to produce the 5-styryl-1,3,4-oxadiazole derivatives (**17a–c**, **18a–c**) in moderate yields. Interconversion of oxadiazole to thiadiazole was affected by the reaction of (**17a–c**, **18a–c**) with thiourea in refluxing tetrahydrofuran to obtain 5-styryl-1,3,4-thiadiazole (**19a–c**, **20a–c**) in moderate yields. However, all synthesized compounds were obtained in higher yields and in shorter reaction times by the ultrasound irradiation method when compared to the conventional method (Scheme 1).

**Scheme 2:**
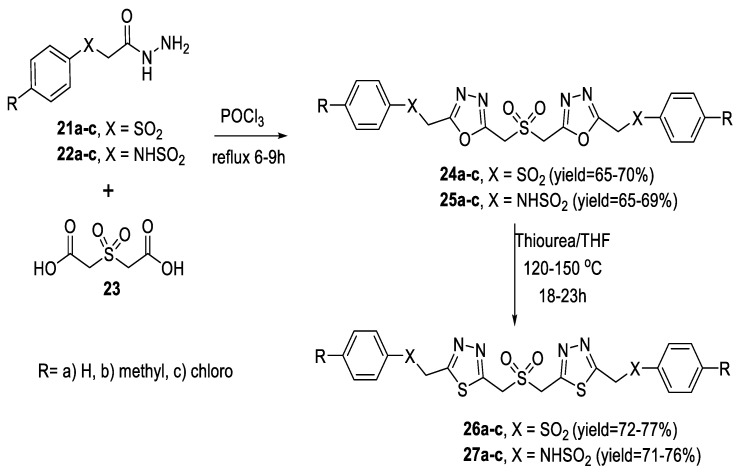
Synthetic pathway for compounds **24–27**.

Similarly, a new class of sulfone/sulfonamide-linked bis(1,3,4-oxadiazoles) (**24a–c**, **25a–c**) was prepared by the cyclocondensation of arylsulfonylacetic acid hydrazide (**21a–c**) or arylaminosulfonylacetic acid hydrazide (**22a–c**) with sulfonyldiacetic acid **23** in the presence of POCl_3_. Then, the bis(1,3,4-oxadiazoles) (**24a–c**, **25a–c**) were converted to bis(1,3,4-thiadiazoles) (**26a–c**, **27a–c**) by heating with thiourea in THF (Scheme 2) [2]. 

**Scheme 3:**
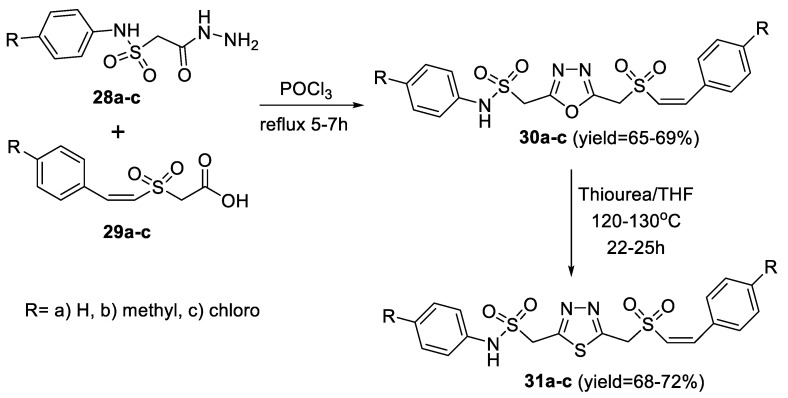
Synthetic pathway for compounds **30a–c** and **31a–c**.

The same methodology was used by Padmaja et al., in the preparation of styrylsulfonylmethyl 1,3,4-oxadiazoles (**30a–c**) through cyclocondensation of the intermediate arylaminosulfonylacetic acid hydrazides (**28a–c**) with *Z*-styrylsulfonylacetic acid (**29a–c**). The obtained oxadiazoles were then treated with thiourea in THF to undergo interconversion to the corresponding thiadiazoles (**31a–c**) (Scheme 3) [49].

**Scheme 4:**
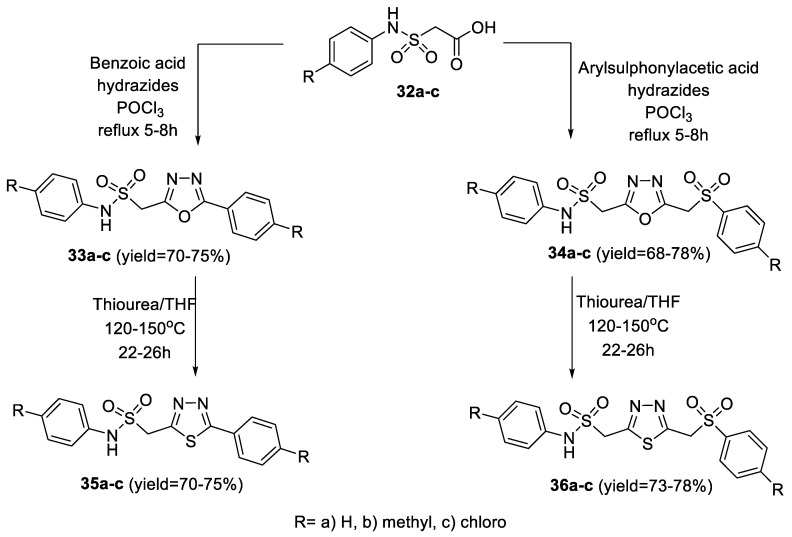
Synthetic pathway for compounds **33a–c**–**36a–c**.

In 2010, Padmavathi et al., synthesized a series of 2-arylaminosulfonylmethyl-5-aryl-1,3,4-oxadiazoles (**33a–c**) and 2-arylaminosulfonylmethyl-5-arylsulfonylmethyl-1,3,4-oxadiazoles (**34a–c**) by the cyclocondensation of arylaminosulfonylacetic acids (**32a–c**) with different aryl acid hydrazides and arylsulfonylacetic acid hydrazides in the presence of POCl_3_, respectively. Interconversion of (**33a–c**, **34a–c**) to thiadiazoles (**35a–c**, **36a–c**) was affected by treatment with thiourea in THF (Scheme 4) [50].

**Scheme 5:**
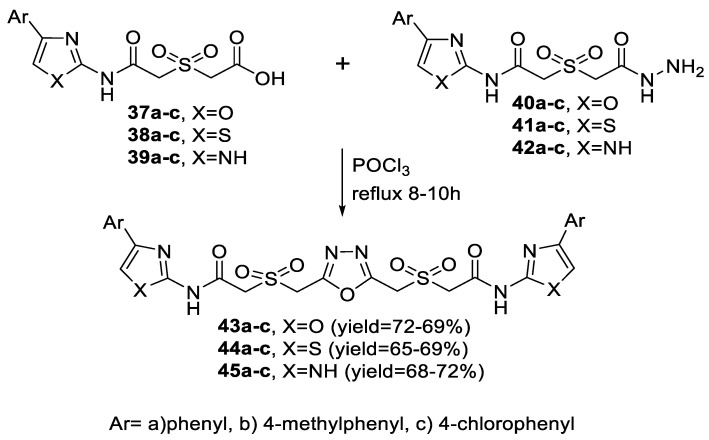
Synthetic pathway for compounds **43a–c**–**45a–c**.

A variety of symmetrical 2,4-bis-oxazolyl/thiazolyl/imidazolylacetamido-sulfonylmethyl 1,3,4-oxadiazoles (**43a–c**, **44a–c**, **45a–c**) were prepared via a one-step route through the reaction of different 2-((4-aryl(oxazole or thiazole or 1*H*-imidazole)-2-ylcarbamoyl)methylsulfonyl)-acetic acid derivatives (**37a–c**, **38a–c**, **39a–c**) with the respective acetohydrazides (**40a–c**, **41a–c**, **42a–c**) upon heating under reflux in POCl_3_ (Scheme 5).

**Scheme 6:**
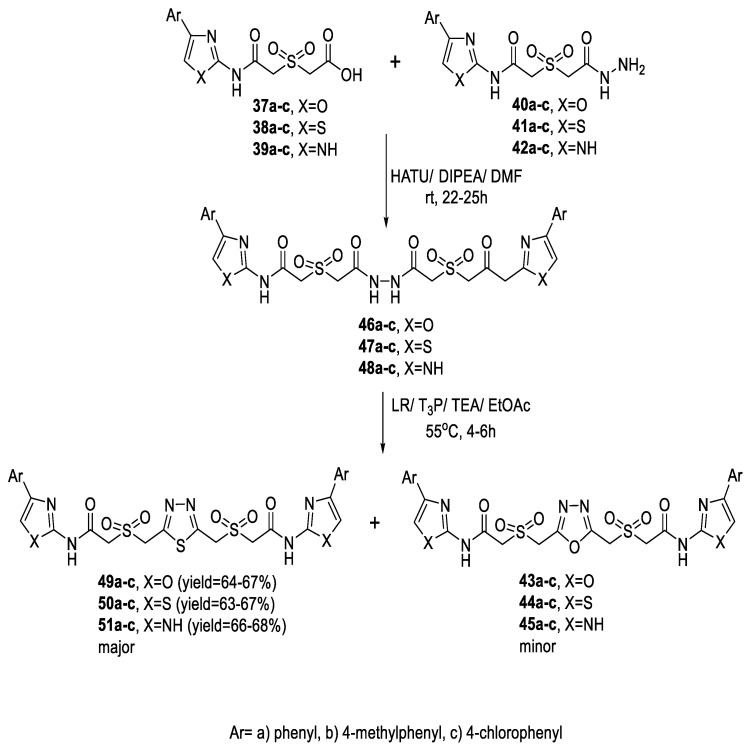
Synthetic pathway for compounds **49a–c**–**51a–c**.

For the thiadiazole analogs, a two-step route that involved the synthesis of the bis-hydrazide intermediate followed by cyclization was adopted. The bis-hydrazides (**46a–c**, **47a–c**, **48a–c**) were prepared by coupling the carboxylic acid derivatives (**37a–c**, **38a–c**, **39a–c**) with the acetohydrazide derivatives (**40a–c**, **41a–c**, **42a–c**) in the presence of 1-[bis(dimethylamino)methylene]-1*H*1,2,3-triazolo [4,5-b]pyridinium-3-oxid hexafluorophosphate (HATU) and *N*,*N*-diisopropyl ethylamine (DIPEA) in DMF. Then, cyclocondensation of (**46a–c**, **47a–c**, **48a–c**) with Lawesson’s reagent (LR), propylphosphonic anhydride (T_3_P) and TEA produced the corresponding 1,3,4-thiadiazole (**49a–c**, **50a–c**, **51a–c**) (Scheme 6) [51].

### 2.2. Synthesis from 1,2-Diacylhydrazines

**Figure 10:**
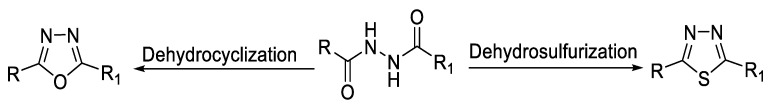
Types of reactions of 1,2-diacylhydrazines forming the 1,3,4-oxa-/thiadiazole ring.

**Figure 11:**
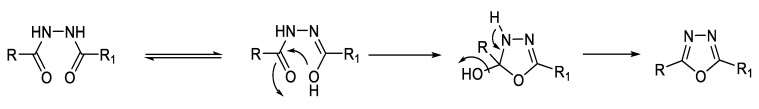
Mechanism of dehydrocyclization of 1,2-diacylhydrazines to 1,3,4-oxadiazoles [53].

**Figure 12:**
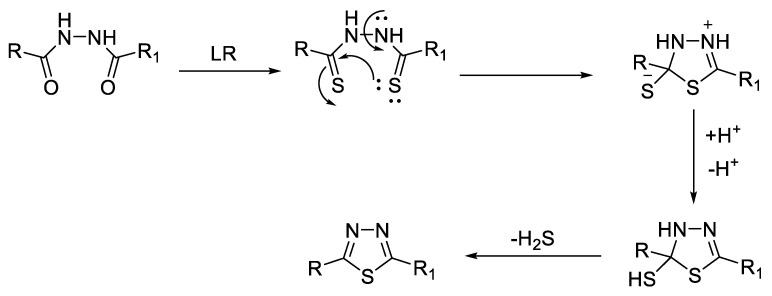
Mechanism of dehydrosulfurization of 1,2-diacylhydrazines to 1,3,4-thiadiazoles [57].

1,2-Diacylhydrazines (Figure 10) can undergo dehydrocyclization reaction, forming 1,3,4-oxadiazoles using cyclodehydrating agents such as POCl_3_, P_2_O_5_, polyphosphoric acid (PPA) [52], *N*,*N′*-dicyclohexylcarbodiimide (DCC) [53], or ethyl-3-(3-dimethylaminopropyl) carbodiimide (EDC) [54] (Figure 11). They can also be converted to the 1,3,4-thiadiazole ring by dehydrosulfurization reaction using Lawesson’s reagent (LR) [55] or P_2_S_5_ [56] (Figure 12).

**Scheme 7:**
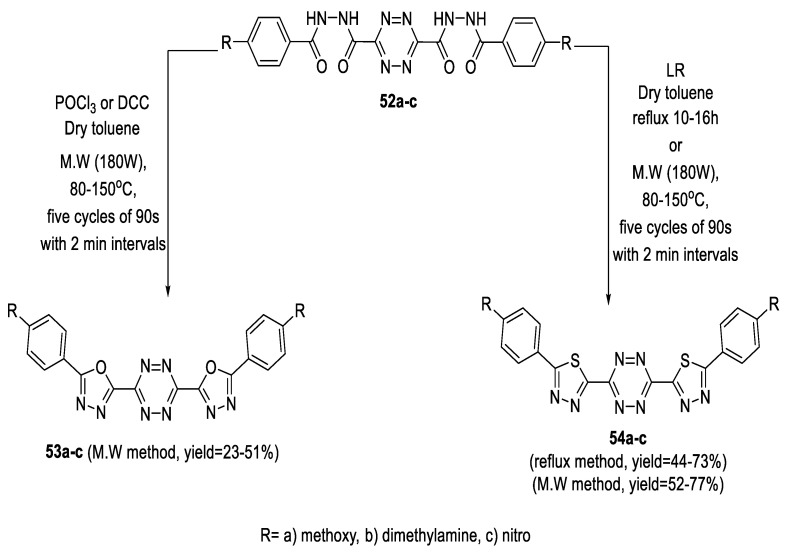
Synthetic pathway for compounds **53a–c** and **54a–c**.

*s*-Tetrazine-3,6-dicarbohydrazides (**52a–c**) can be reacted at high temperature with cyclodehydrating reagents such as POCl_3_ or DCC in dry toluene to obtain conjugated *s*-tetrazine-1,3,4-oxadiazole hybrids. This reaction was unsuccessful under conventional conditions; however, employing microwave irradiation led to the desired 1,3,4-oxadiazoles (**53a–c**) in good yields. *S*-Tetrazine-3,6-dicarbohydrazides (**52a–c**) were also treated with LR in dry toluene under conventional heating and microwave irradiation, with both methods resulting in the formation of s-tetrazine-1,3,4-thiadiazole derivatives (**54a–c**) in satisfactory yields (Scheme 7) [53]. 

**Scheme 8:**
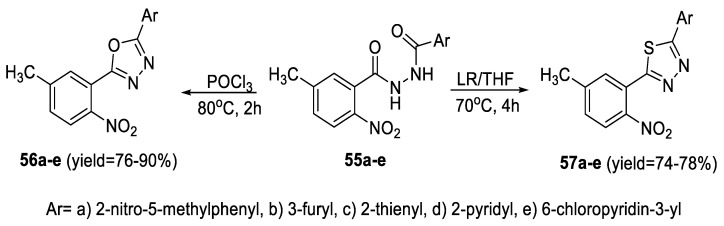
Synthetic pathway for compounds **56a–e** and **57a–e**.

The key intermediate *N*-(5-methyl-2-nitrobenzoyl)-2-substituted carbohydrazides (**55a–e**) were treated separately with POCl_3_ and LR to obtain the desired 2-aryl-5-substituted-1,3,4-oxadiazoles (**56a–e**) and thiadiazoles (**57a–e**), respectively. The yields of 1,3,4- thiadiazoles were low compared to those of oxadiazoles (Scheme 8) [55]. 

**Scheme 9:**
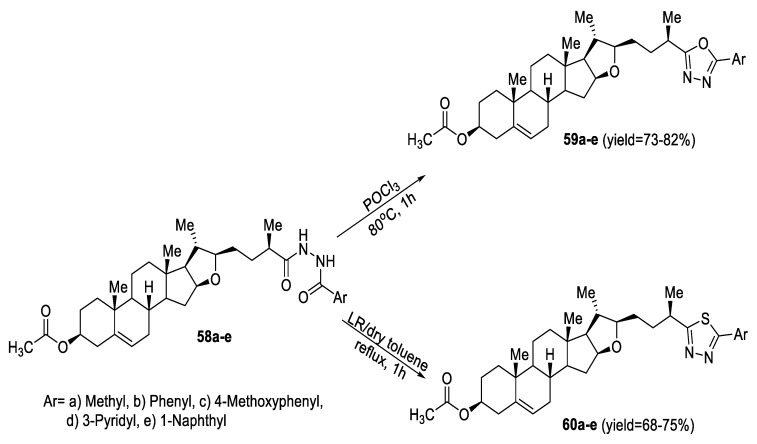
Synthetic pathway for compounds **59a–e** and **60a–e**.

Two series of novel diosgenin derivatives bearing 1,3,4-oxadiazole (**59a–e**) or 1,3,4-thiadiazole (**60a–e**) moieties were designed and synthesized. The cyclodehydration of the N,N′-disubstituted hydrazine intermediates (**58a–e**) with POCl_3_ furnished the corresponding 1,3,4-oxadiazoles (**59a–e**) in high yields. On the other hand, heating intermediates (**58a–e**) with LR in dry toluene under reflux conditions induced dehydrosulfurization into the 1,3,4-thiadiazole counterparts (**60a–e**) in moderate yields (Scheme 9) [58].

**Scheme 10:**
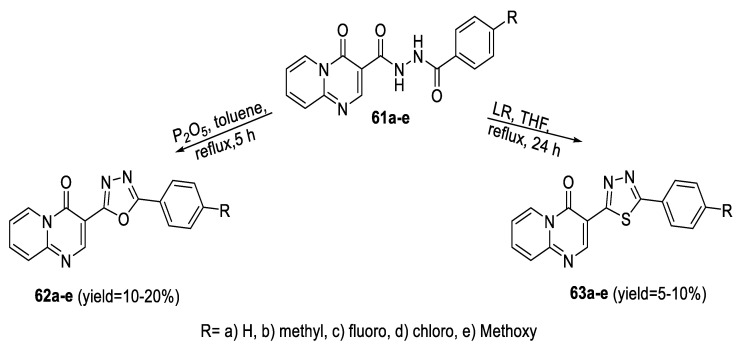
Synthetic pathway for compounds **62a–e** and **63a–e**.

Similarly, a series of 4-oxo-4*H*-pyrido [1,2-a]pyrimidine derivatives containing the 1,3,4-oxadiazole (**62a–c**) or 1,3,4- thiadiazole (**63a–c**) ring were synthesized from the corresponding diacylhydrazines (**61a–e**) by dehydrocyclization using P_2_O_5_ in toluene and dehydrosulfurization by LR in THF (Scheme 10) [59].

**Scheme 11:**
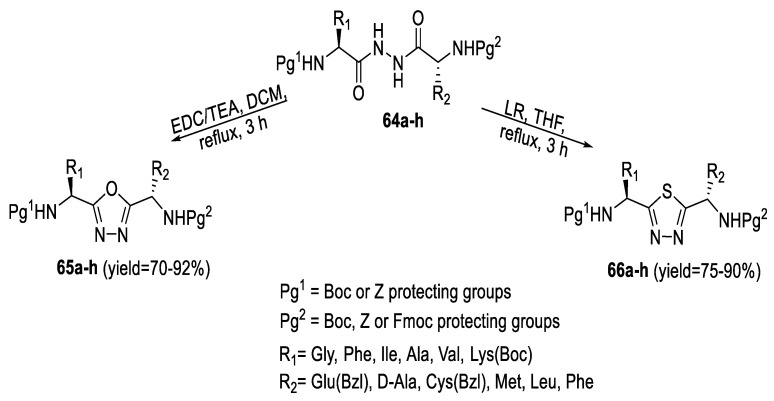
Synthetic pathway for compounds **65a–h** and **66a–h**.

Orthogonally protected 1,3,4-thiadiazole and 1,3,4-oxadiazole-tethered dipeptidomimetics were synthesized from a set of protected diacylhydrazines (**64a–h**) derived from amino acids in good to high yield with good purities and with no detectable enantiomerization. The 1,3,4-thiadiazolo-dipeptides (**66a–h**) were formed by refluxing (**64a–h**) with LR in THF. Furthermore, refluxing (**64a–h**) in dry CH_2_Cl_2_ in the presence of EDC and TEA resulted in the formation of 1,3,4-oxadiazolo-peptides (**65a–h**) by cyclodehydration (Scheme 11) [54].

**Scheme 12:**
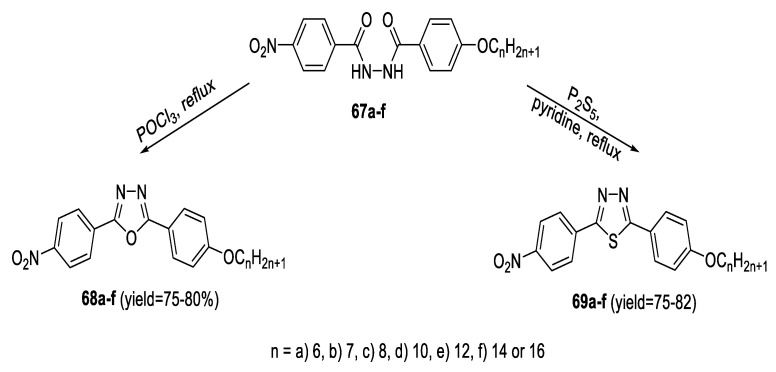
Synthetic pathway for compounds **68a–f** and **69a–f**.

A series of mesogenic bent-shaped 2-(4-nitrophenyl)-5-(4-n-alkoxyphenyl)-1,3,4-oxadiazoles (**68a–f**) was synthesized by refluxing the analogous diacylhydrazine intermediates (**67a–f**) with POCl_3._ The 1,3,4-thiadiazole analogs (**69a–f**) were obtained by dehydrosulfurization of the same intermediates through heating under reflux with P_2_S_5_ in dry pyridine (Scheme 12) [56].

**Scheme 13:**
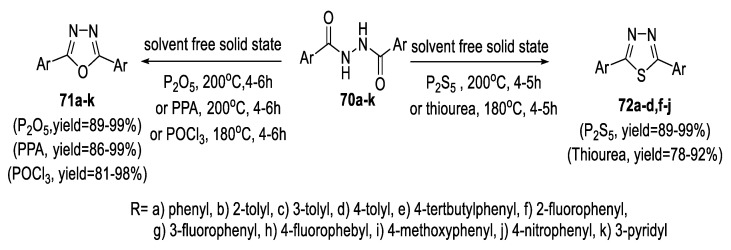
Synthetic pathway for compounds 7**1a–k** and **72a–d**,**f–j**.

A solvent-free solid-state method was developed to synthesize a series of substituted 1,3,4-oxadiazoles (**71a–k**) and 1,3,4-thiadiazoles (**72a–d**,**f-j**) derivatives in high yields by cyclodehydration of *N*,*N’*-bishydrazide derivatives (**70a–k**). For cyclization to 1,3,4-oxadiazoles (**71a–k**), three kinds of cyclizing agents, P_2_O_5_, PPA, and POCl_3_, were used. It was found that the three reagents worked well and provided the products with high yields. On the other hand, P_2_S_5_ and thiourea were used as cyclizing agents to promote the cyclization to 1,3,4-thiadiazoles (**72a–d**,**f-j**). Due to the decomposition of thiourea at high temperatures, the reaction yield using thiourea was lower than that of P_2_S_5_. Generally, P_2_O_5_ and P_2_S_5_ act not only as cyclizing agents but also as dehydrating agents, which further promote cyclodehydration (Scheme 13) [52].

### 2.3. Synthesis from Acyl Semi/Thiosemicarbazides

**Figure 13:**
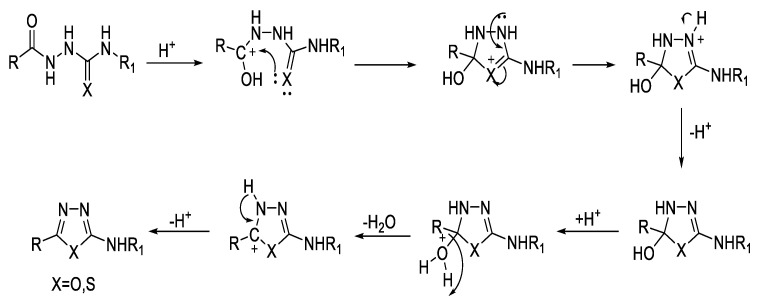
Proposed mechanism for the synthesis of 2,5-disubstituted-1,3,4-oxa-/thiadiazoles using acyl semi/thiosemicarbazides based on the reported mechanism for synthesis of 2,5-disubstituted-1,3,4-thiadiazoles from acyl thiosemicarbazide [65].

Aryl amino-1,3,4-oxadiazoles can be cyclized from acyl semicarbazides under the effect of acid through treatment with POCl_3_ [60]; likewise, acyl thiosemicarbazides can be dehydrocyclized to aryl amino 1,3,4-thiadiazoles under acidic conditions using conc. H_2_SO_4_ [61], H_3_PO_4_ [60], POCl_3_ [62], p-TsCl in TEA [63], or acetic anhydride [64] (Figure 13).

**Figure 14:**
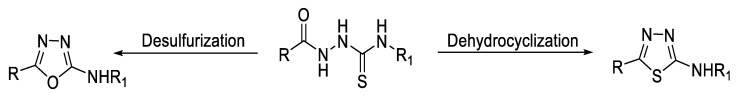
Types of reactions of thiosemicarbazides forming 1,3,4-oxa-/thiadiazole rings.

1,3,4-Oxadiazoles can be also synthesized from acyl thiosemicarbazides through oxidative desulfurization reaction using one of the following reagents: I_2_/KI in NaOH [61], HgO in ethanol [62], Hg(OAc)_2_ in ethanol [66], HgCl_2_ in triethylamine (TEA) [67], or EDC.HCl in DMSO (Figure 14) [63].

**Scheme 14:**
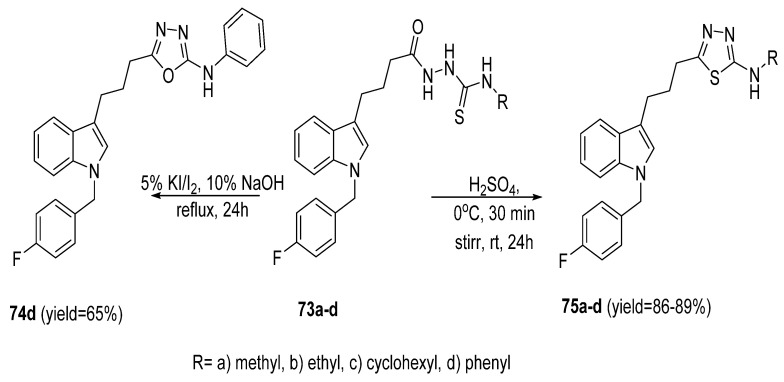
Synthetic pathway for compounds **74d** and **75a–d**.

A novel series of 5-[*N*-(*p*-fluorobenzyl)indolyl]propyl-1,3,4-thiadiazoles (**75a–d**) was prepared via acid-catalyzed dehydrocyclization of thiosemicarbazides (**73a–d**) in the presence of H_2_SO_4_ at 0 °C in 86–89% yields. Treatment of 4-*N*-phenyl thiosemicarbazide **73d** with 5% (I_2_/KI) in the presence of 10% NaOH produced the corresponding 2-*N*-phenylamine-1,3,4-oxadiazole **74d** (Scheme 14) [61].

**Scheme 15:**
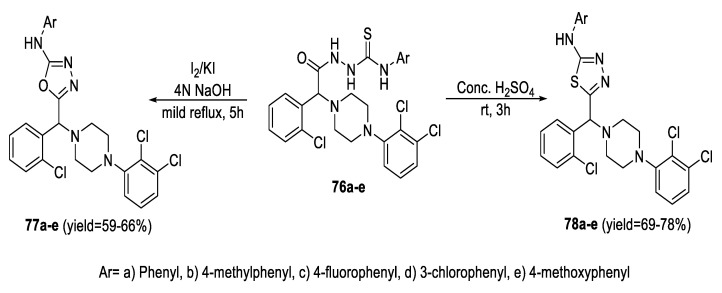
Synthetic pathway for compounds **77a–e** and **78a–e**.

Similarly, cyclization of 4-substituted 1-{2-(2-chlorophenyl)-2-[4-(2,3-dichlorophenyl)piperazin-1-yl]acetyl}thiosemicarbazide (**76a–e**) under acidic conditions yielded 1,3,4-thiadiazoles (**78a–e**), whereas 1,3,4-oxadiazoles (**77a–e**) were prepared by treating (**76a–e**) with iodine and KI in 4 N NaOH (Scheme 15) [68].

**Scheme 16:**
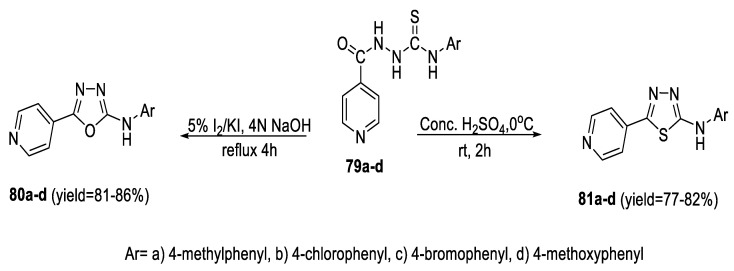
Synthetic pathway for compounds **80a–d** and **81a–d**.

Oxidative cyclization of 2-isonicotinyl hydrazinecarbothioamide derivatives (**79a–d**) in 4 N NaOH using a mixture of I_2_/KI (5%) furnished the corresponding 5-(pyridin-4-yl)-*N*-substituted-1,3,4-oxadiazol-2-amines (**80a–d**) via elimination of H_2_S. On the other hand, 5-(pyridin-4-yl)-*N*-substituted-1,3,4-thiadiazol-2-amines (**81a–d**) were obtained by cyclization of (**79a–d**) upon reaction with cold conc. H_2_SO_4_ (Scheme 16) [69].

**Scheme 17:**
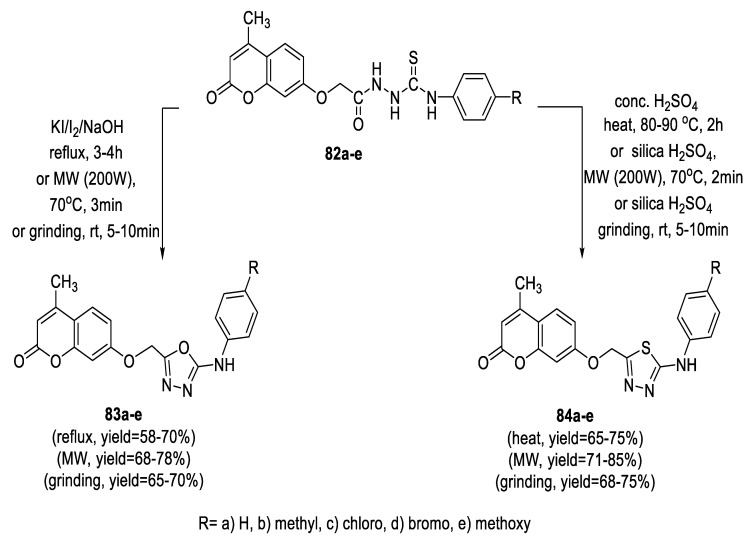
Synthetic pathway for compounds **83a–e** and **84a–e**.

Novel coumarin derivatives possessing 2-arylamino-1,3,4-thiadiazole (**84a–e**) and 1,3,4- oxadiazole (**83a–e**) moieties were efficiently synthesized through cyclization of thiosemicarbazides (**82a–e**) using conventional methods. The intramolecular cyclocondensation of (**83a–e**) in the presence of concentrated H_2_SO_4_ formed the corresponding 1,3,4-thiadiazoles (**84a–e**) in good yields, whereas the cyclocondensation of (**82a–e**) in the presence of NaOH/KI/I_2_ in ethanol formed the corresponding 1,3,4-oxadiazoles (**83a–e**) in lower yields. Furthermore, the same compounds were also prepared by the microwave irradiation method and grinding method, with higher yields and shorter reaction times (Scheme 17) [70].

**Scheme 18:**
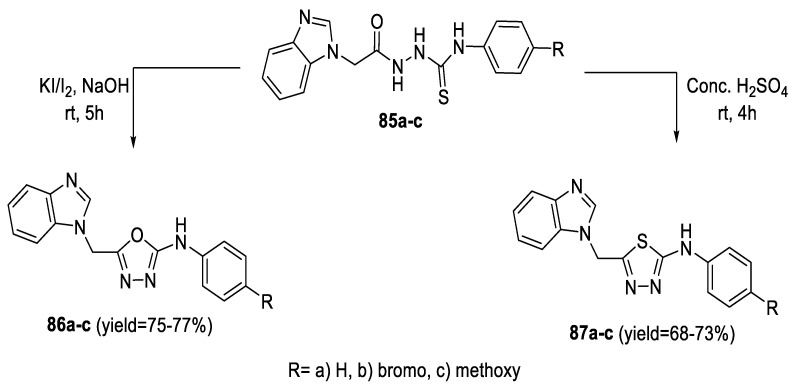
Synthetic pathway for compounds **86a–c** and **87a–c**.

Similarly, benzimidazole derivatives featuring 1,3,4-oxadiazol-2-amine (**86a–c**) and 1,3,4-thiadiazol-2-amine (**87a–c**) heterocyclic moieties were prepared from acyl thiosemicarbazide intermediates (**85a–c**) via intramolecular cyclization with I_2_/KI/4N NaOH and conc. H_2_SO_4_, respectively (Scheme 18) [71].

**Scheme 19:**
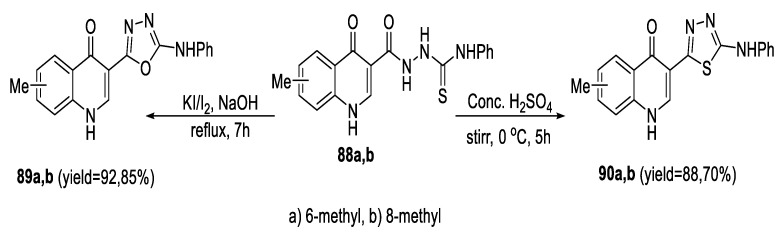
Synthetic pathway for compounds **89a,b**, and **90a,b**.

Series of substituted 1,4-dihydro-4-oxoquinoline bearing 2-arylamino-1,3,4-oxadiazole (**89a,b**) or 1,3,4-thiadiazole (**90a,b**) were synthesized by El-Essawy et al. [72]. The 1,3,4-oxadiazole derivatives (**89a,b**) were obtained through oxidative cyclization of the acyl thiosemicarbazide intermediates (**88a,b**) using I_2_/KI in ethanolic NaOH. Intramolecular dehydration of (**88a,b**) with cold-concentrated H_2_SO_4_ furnished the 1,3,4-thiadiazole counterparts (**90a,b**) (Scheme 19).

**Scheme 20:**
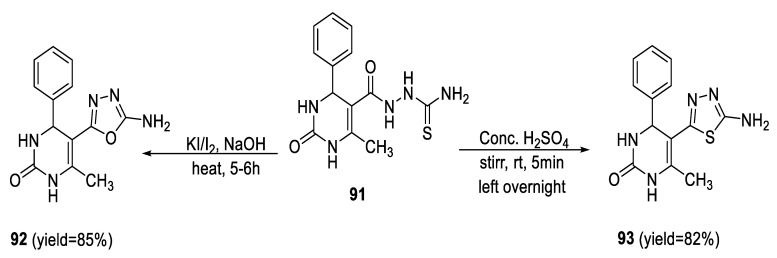
Synthetic pathway for compounds **92** and **93**.

In a different study, the tetrahydropyrimidinone derivative bearing a 2-amino-1,3,4- thiadiazole motif at position 5 **93** was synthesized by treatment of the key intermediate tetrahydro pyrimidine-(5)-3-carbothioamide **91** with conc. H_2_SO_4_. Furthermore, the oxadiazole derivative **92** was prepared by heterocyclization of **91** when treated with I_2_/KI in a basic medium (10% NaOH) (Scheme 20) [73].

**Scheme 21:**
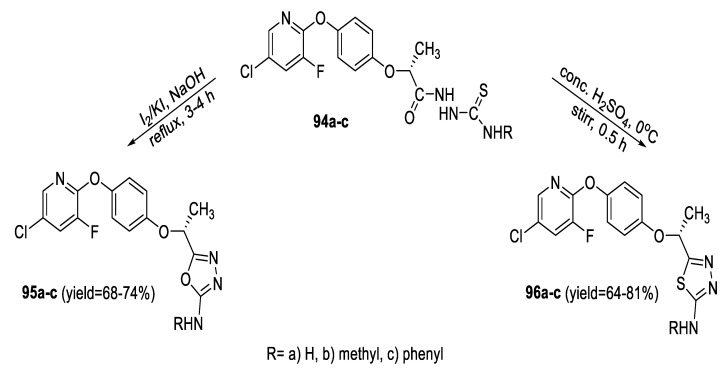
Synthetic pathway for compounds **95a–c** and **96a–c**.

A series of *N*-substituted 2-amino-1,3,4-thiadiazoles (**96a–c**) and 1,3,4-oxadiazoles (**95a–c**) carrying an (R) 5-(1-(4-(5- chloro-3-fluoropyridin-2-yloxy)phenoxy)ethyl) moiety were synthesized from acyl thiosemicarbazide intermediates (**94a–c**) by cyclodehydration using conc. H_2_SO_4_ and cyclodesulfurization with alkaline iodine, respectively (Scheme 21) [74].

**Scheme 22:**
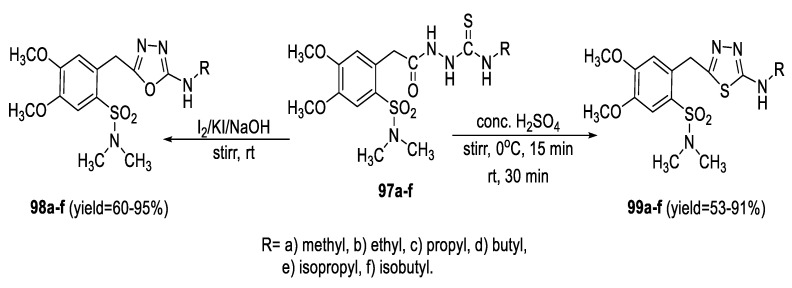
Synthetic pathway for compounds **98a–f** and **99a–f**.

In a similar approach, Zoumpoulakis et al., synthesized a series of *N*,*N*-dimethylsulphonamide-based 1,3,4-thiadiazoles (**99a–f**) and 1,3,4-oxadiazoles (**98a–f**) utilizing the acylthiosemicarbazide intermediates (**97a–f**) through dehydrative cyclization in concentrated H_2_SO_4_ and desulfurative cyclization with I_2_/NaOH, respectively (Scheme 22) [75].

**Scheme 23:**
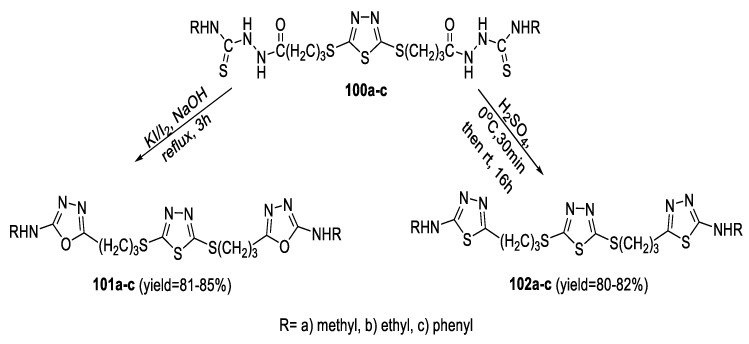
Synthetic pathway for compounds **101a–c** and **102a–c**.

Thermal intramolecular cyclodehydration of the bis- acyl thiosemicarbazide intermediates (**100a–c**) was performed by the action of H_2_SO_4_ at 0 °C to furnish the corresponding 2-aminoalkyl/aryl-1,3,4-thiadiazoles (**102a–c**). Alternatively, compounds (**100a–c**) were oxidatively cyclized to the corresponding 2-alkyl/arylamino-1,3,4-oxadiazoles (**101a–c**) in the presence of I_2_/KI in refluxing (4%) NaOH (Scheme 23) [76].

**Scheme 24:**
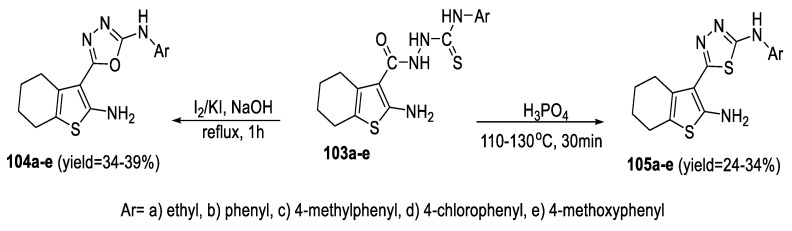
Synthetic pathway for compounds **104a–e** and **105a–e**.

A series of 2-aminotetrahydrobenzothiophene linked to arylamino 1,3,4-oxadiazole (**104a–e**) and 1,3,4-thiadiazole (**105a–e**) moieties were synthesized from the precursor acylthiosemicarbazides (**103a–e**) by cyclization using I_2_/KI in NaOH and orthophosphoric acid, respectively (Scheme 24) [77].

**Scheme 25:**
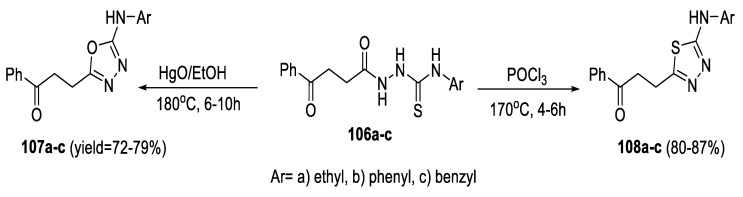
Synthetic pathway for compounds **107a–c** and **108a–c**.

In 2020, Abu-Hashem synthesized a variety of heterocyclic compounds through acyl thiosemicarbazide intermediates (**106a–c**). Desulfurization of (**106a–c**) through refluxing with HgO in ethanol afforded 1,3,4-oxadiazoles (**107a–c**) in moderate yields. The treatment of (**106a–c**) with POCl_3_, led to the formation of 1,3,4-thiadiazoles (**108a–c**) in higher yields than those of oxadiazoles (Scheme 25) [62].

**Scheme 26:**
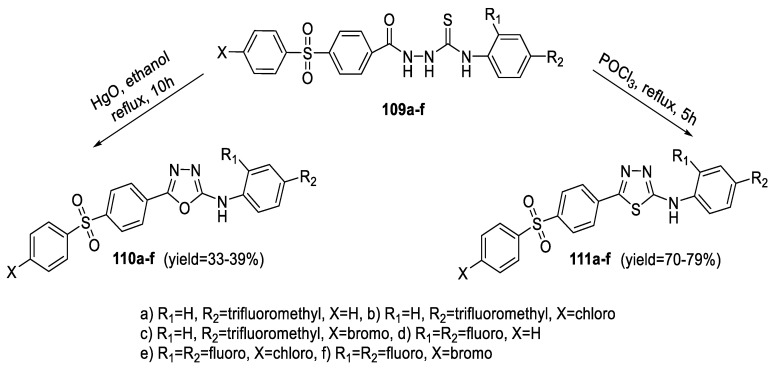
Synthetic pathway for compounds **110a–f** and **111a–f**.

The synthesis of some 1,3,4-thia/oxadiazole derivatives containing arylsulphonylphenyl and 4- trifluoromethylphenyl or 2,4-difluorophenyl moieties was achieved through cyclization of hydrazinecarbothioamides (**109a–f**). Treatment of (**109a–f**) with POCl_3_ resulted in dehydrative cyclization obtaining the 2-arylamino-1,3,4-thiadiazoles (**111a–f**) in moderate yields, while on refluxing of the same intermediates with HgO, desulfurative cyclization took place, resulting in the corresponding 2-arylamino-1,3,4-oxadiazoles (**110a–f**) being obtained in fair yields (Scheme 26) [78,79].

**Scheme 27:**
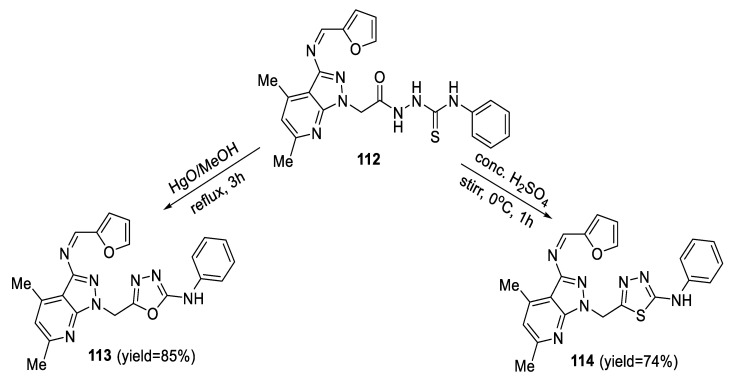
Synthetic pathway for compounds **113** and **114**.

Treatment of *N*-(furan-2-ylmethylidene)-4,6-dimethyl-1-*H*-prazolo [3,4,b]pyridine-1-yl acetyl thiosemicarbazide **112** with ice-cooled concentrated H_2_SO_4_ resulted in the formation of the respective 1,3,4-thiadiazole derivative **114**. On the other hand, heating intermediate **112** with HgO in methanol resulted in desulfurative cyclization, which afforded the 1,3,4-oxadiazole derivative **113** (Scheme 27) [80].

**Scheme 28:**
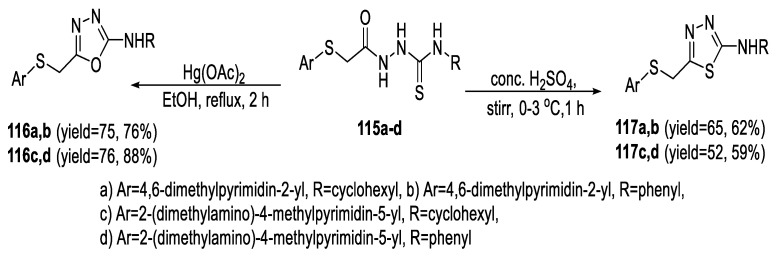
Synthetic pathway for compounds **116a–d** and **117a–d**.

Burbuliene et al., developed concise and efficient procedures for the synthesis of 1,3,4-oxadiazoles and 1,3,4-thiadiazoles bearing 4-pyrimidinylthio- and 2-pyrimidinylthio- moieties by intramolecular cyclization of substituted acyl thiosemicarbazide precursors (**115a–d**). The cyclodesulfurization to form 1,3,4-oxadiazoles (**116a–d**) was conducted by treating (**115a–d**) with Hg(OAc)_2_ in ethanol. On the other hand, cyclodehydration of (**115a–d**) in acidic media using concentrated H_2_SO_4_ at ∼0 °C followed by neutralization with NH_4_OH provided the thiadiazoles (**117a–d**) in lower yields than those of the oxadiazole analogs (Scheme 28) [66].

**Scheme 29:**
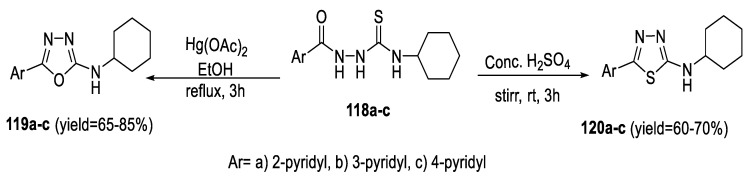
Synthetic pathway for compounds **119a–c** and **120a–c**.

Series of 1,3,4-thiadiazoles (**120a–c**) and 1,3,4-oxadiazoles (**119a–c**) containing isomeric pyridyl and cyclohexyl moieties were synthesized by intramolecular cyclization of 1,4-disubstituted acyl thiosemicarbazides (**118a–c**). The latter underwent cyclodehydration in the acid medium of conc. H_2_SO_4_ to form 1,3,4-thiadiazoles (**120a–c**). The 1,3,4-oxadiazoles (**119a–c**) were obtained from the corresponding thiosemicarbazides in the presence of Hg(OAc)_2_ as an oxidizing agent (Scheme 29) [81].

**Scheme 30:**
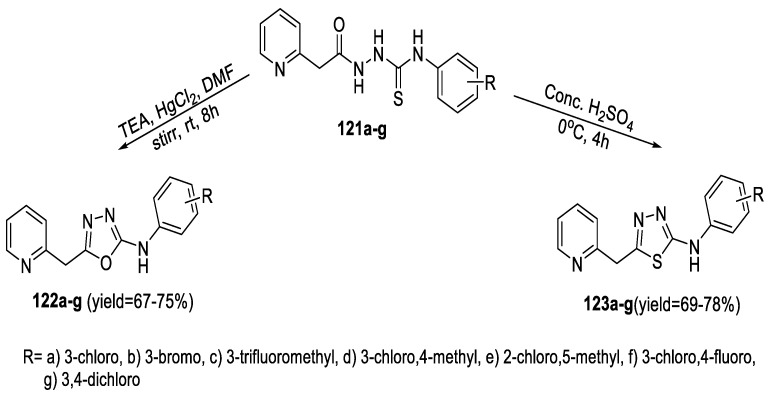
Synthetic pathway for compounds **122a–g** and **123a–g**.

Novel heterocyclic derivatives of 2-arylamino-5-(pyridin-2-ylmethyl)-1,3,4-oxadiazoles (**122a–g**) and thiadiazoles (**123a–g**) were efficiently synthesized through (pyridin-2-yl-acetyl)hydrazinecarbothioamide derivatives (**121a–g**). The method for obtaining 1,3,4-thiadiazole derivatives (**123a–g**) required cyclization under acidic conditions (conc. H_2_SO_4_), while oxadiazoles (**122a–g**) were prepared by a desulfurization/cyclization reaction of (**121a–g**) using HgCl_2_ with optional catalytic amounts of TEA (Scheme 30) [67].

**Scheme 31:**
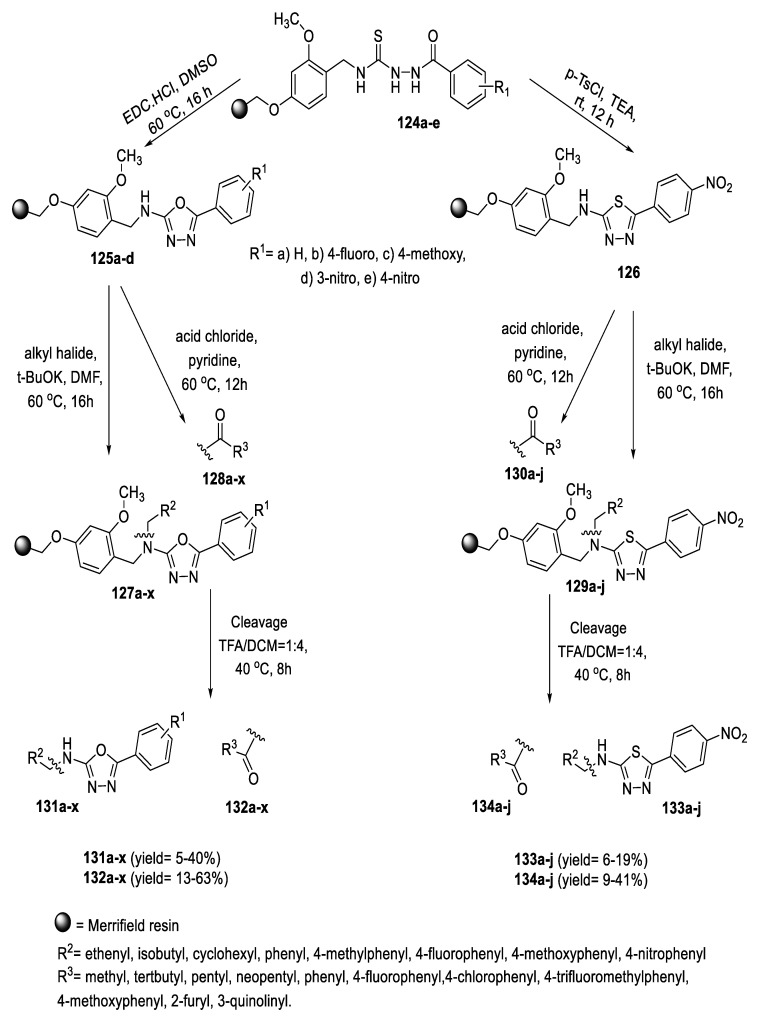
Synthetic pathway for compounds **125a–d**–**134a–j**.

Polymer-bound 2-amino-1,3,4-oxadiazoles (**125a–d**) and 1,3,4-thiadiazole **126** core skeleton resin were synthesized by solid-phase cyclization of acyl thiosemicarbazides (**124a–e**) with EDC·HCl and *p*-TsCl, respectively, followed by a functionalization reaction of the resulting core skeleton with various electrophiles such as alkyl halides and acid chlorides to generate *N*-alkylamino and *N*-acylamino-1,3,4-oxadiazole (**127a–x** and **128a–x**) and 1,3,4-thiadiazole (**129a–j** and **130a–j**) resin, respectively. Finally, the desired 2-amino and 2-amido-1,3,4-oxadiazole (**131a–x** and **132a–x**) and 1,3,4-thiadiazole (**133a–j** and **134a–j**) were then generated in high purities by cleavage of the resin under trifluoroacetic acid (TFA) in dichloromethane (DCM) (Scheme 31) [63].

**Scheme 32:**
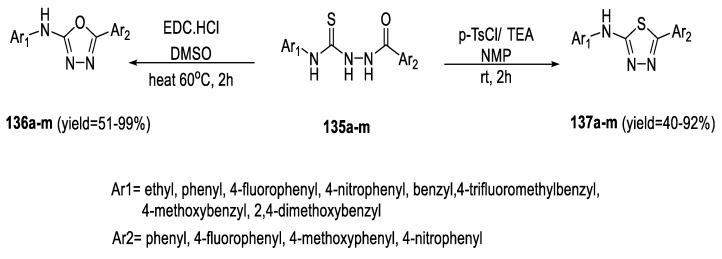
Synthetic pathway for compounds **136a–m** and **137a–m**.

In 2013, a regioselective, reagent-based method for the cyclization reaction of acyl thiosemicarbazide (**135a–m**) into 2-amino-1,3,4-oxadiazole (**136a–m**) and 2-amino-1,3,4- thiadiazole (**137a–m**) core skeletons was described. The study showed that the use of EDC·HCl in DMSO as a desulfurizing agent led to 2-amino-1,3,4-oxadiazoles (**136a–m**) in quantitative yields (regioselectivity ratio = 100:0). However, the use of *p*-TsCl as a dehydrating agent and triethylamine in *N*-methyl-2-pyrrolidone as a polar solvent gave 2-amino-1,3,4-thiadiazoles (**137a–m**) as a major product with regioselectivity of 96:4 and a high yield (Scheme 32) [82].

**Scheme 33:**
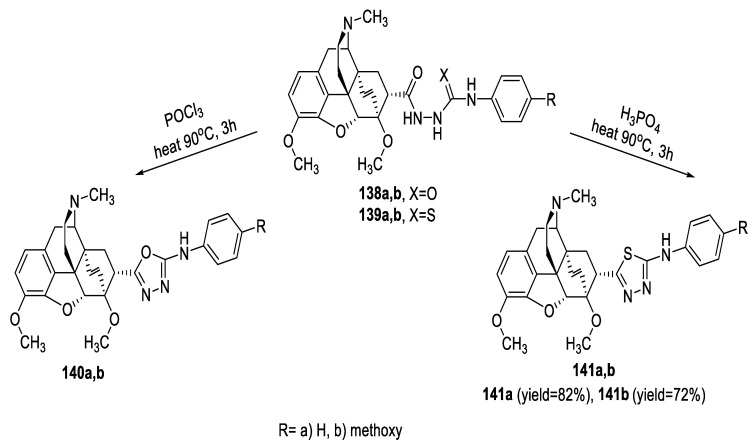
Synthetic pathway for compounds **140a,b** and **141a,b**.

Yavus et al. synthesized some endoethenotetrahydrothebaine derivatives incorporating 1,3,4-oxadiazole (**140a,b**) or 1,3,4-thiadiazole moiety (**141a,b**) by cyclodehydration of the intermediate acylsemicarbazides (**138a,b**) and acylthiosemicarbazides (**139a,b**) using the cyclizing agents POCl_3_ and H_3_PO_4_, respectively (Scheme 33) [60].

**Scheme 34:**
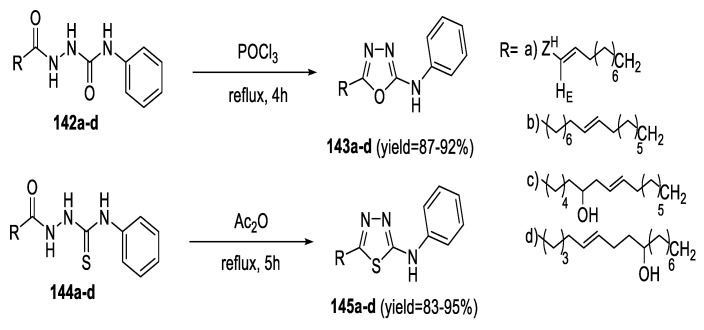
Synthetic pathway for compounds **143a–d**, **145a–d**.

Long-chain 5-alkenyl/hydroxyalkenyl-2-phenylamine-1,3,4-oxadiazoles and 1,3,4-thiadiazoles were synthesized from acyl semicarbazides and thiosemicarbazides, respectively. Refluxing semicarbazide (**142a–d**) with POCl_3_ yielded 2,5-disubstituted- 1,3,4-oxadiazoles (**143a–d**) in good yields. The dehydrative cyclization of thiosemicarbazides (**144a–d**) by Ac_2_O produced 2,5-disubstituted- 1,3,4-thiadiazoles (**145a–d**) in excellent yields (Scheme 34) [64].

### 2.4. Synthesis from Semi-/Thiosemicarbazides

**Figure 15:**
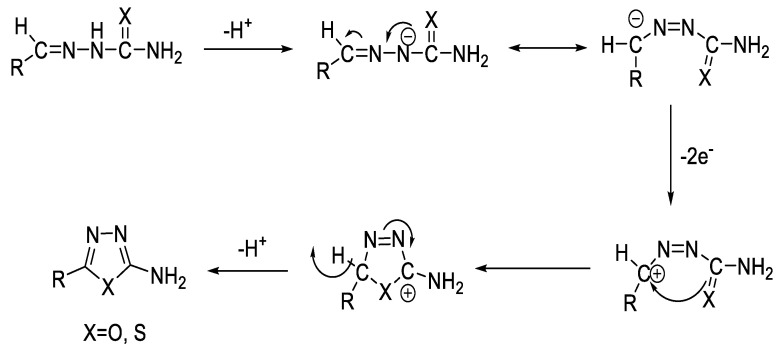
Proposed mechanism of electroxidative cyclization of semi-/thiosemicarbazones into 1,3,4-oxa-/thiadiazoles based on the reported mechanism in literature [86].

Semi-/thiosemicarbazides are reacted with aldehydes in the presence of Na acetate in ethanol, forming the corresponding semi-/thiosemicarbazones, which can be used for the synthesis of 1,3,4-oxa-/thiadiazoles by oxidative cyclization using I_2_/K_2_CO_3_ [83], Br_2_/CH_3_COOH [84], or nitroalkanes in PPA [85] (Figure 15).

**Scheme 35:**
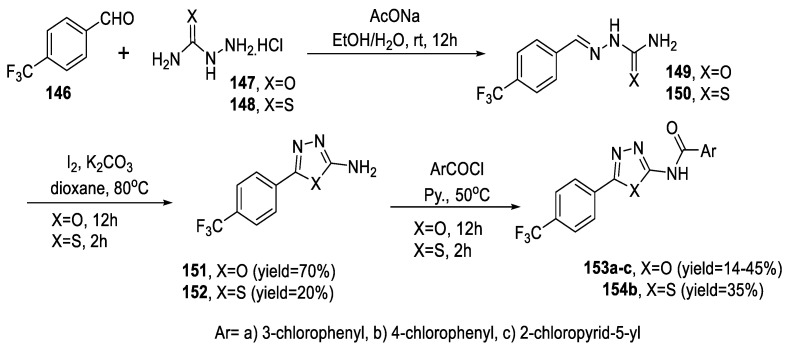
Synthetic pathway for compounds **153a–c** and **154b**.

The reaction of trifluoromethyl benzaldehyde **146** with semi- or thiosemicarbazide hydrochloride **147** and **148** formed the corresponding semi- and thiosemicarbazone **149** and **150**. Cyclization using I_2_/dioxane produced the 1,3,4-oxadiazol-2-amine **151** and 1,3,4-thiadiazol-2-amino **152**, respectively, which were further reacted with aroyl chlorides to give the desired 1,3,4-oxadiazol-2-yl/1,3,4-thiadiazol-2-yl benzamides (**153a–c**) and **154b**. Oxadiazoles were produced in higher yields than thiadiazoles (Scheme 35) [87].

**Scheme 36:**
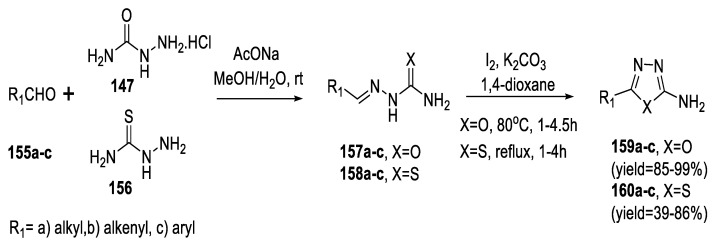
Synthetic pathway for compounds **159a–c** and **160a–c**.

In 2015, 2-amino-substituted 1,3,4-oxadiazoles (**159a–c**) and 1,3,4-thiadiazoles (**160a–c**) were smoothly produced via condensation of semicarbazide hydrochloride/thiosemicarbazide **147** and **156** and the corresponding aldehydes (**155a–c**), followed by I_2_-mediated oxidative C−O/C−S bond formation using a mixture of I_2_ and K_2_CO_3_ in 1,4-dioxane. The 1,3,4-oxadiazoles were produced in higher yields than the 1,3,4-thiadiazoles (Scheme 36) [83].

**Scheme 37:**
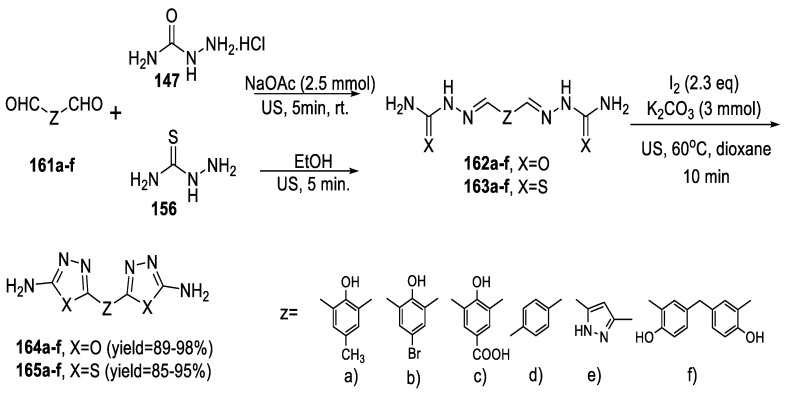
Synthetic pathway for compounds **164a–f** and **165a–f**.

In 2017, Arafa and Abdelmagied reported that a rapid ultrasound accelerated the conversion of bis-semicarbazone derivatives (**162a–f**) into diverse novel substituted bis-2-amino-1,3,4-oxadiazoles (**164a–f**) in quantitative yields using molecular iodine and potassium carbonate in dioxane as a solvent. Products (**164a–f**) could also be obtained in one-pot synthesis without the isolation of intermediates (**162a–f**). Replacing semicarbazide hydrochloride **147** with thiosemicarbazide **156** produced the desired bis-2-amino-1,3,4-thiadiazoles (**165a–f**) in excellent yields (Scheme 37) [88].

**Scheme 38:**
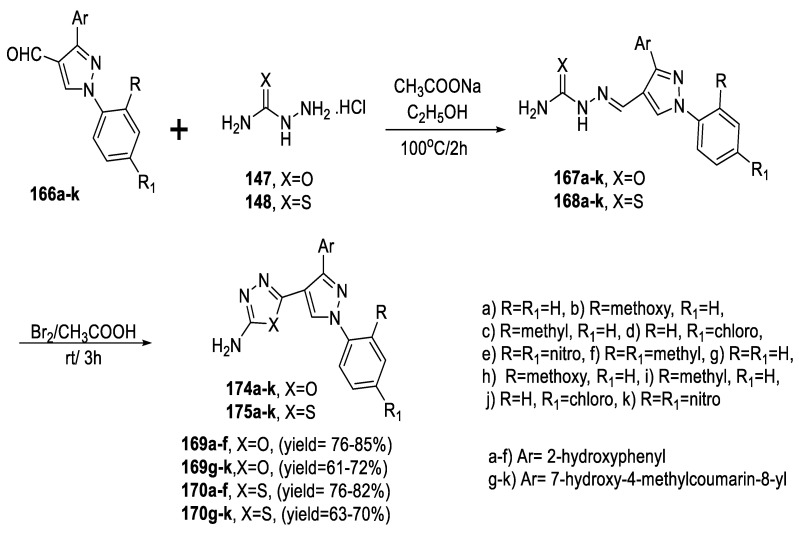
Synthetic pathway for compounds **169a–k** and **170a–k**.

The reaction of pyrazole-4-carboxaldehyde derivatives bearing 2-hydroxyphenyl or 7-hydroxycoumarin-8-yl moiety (**166a–k**) with (semi)thiosemicarbazide hydrochloride **147** or **148** in the presence of sodium acetate in ethyl alcohol under reflux conditions afforded the corresponding semicarbazones (**167a–k**) and thiosemicarbazones (**168a–k**). Their oxidative cyclization using bromine as an oxidant in acetic acid at room temperature yielded the corresponding 1,3,4-oxadiazoles (**169a–k**) and 1,3,4-thiadiazoles (**170a–k**) (Scheme 38) [84,89].

**Scheme 39:**
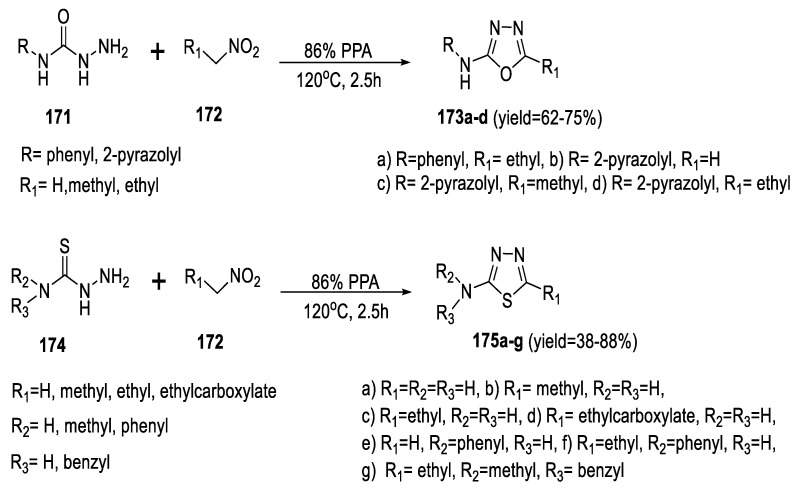
Synthetic pathway for compounds **173a–d** and **175a–g**.

**Figure 16:**
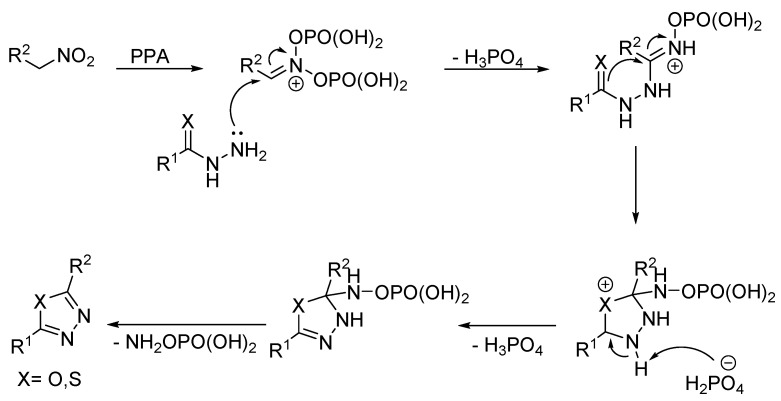
Mechanistic rationale for the formation of compounds **173a–d** and **175a–g** [85].

In 2020, Askenov et al., discovered an unusual reaction (Figure 16) for the synthesis of 2-amino-1,3,4-oxa-/thiadiazoles via the electrophilic activation of nitroalkanes in the presence of polyphosphoric acid followed by a subsequent nucleophilic attack with semi/thiosemicarbazides. It was found that nitroalkanes **172** in PPA converted into phosphorylated nitronates showing strong electrophilic properties. Semicarbazides **171** and thiosemicarbazides **174** were employed as nucleophilic components in this cyclization reaction to access 2-amino-1,3,4-oxadiazoles (**173a–d**) and 2-amino-1,3,4-thiadiazoles (**175a–g**), respectively (Scheme 39) [85].

**Scheme 40:**
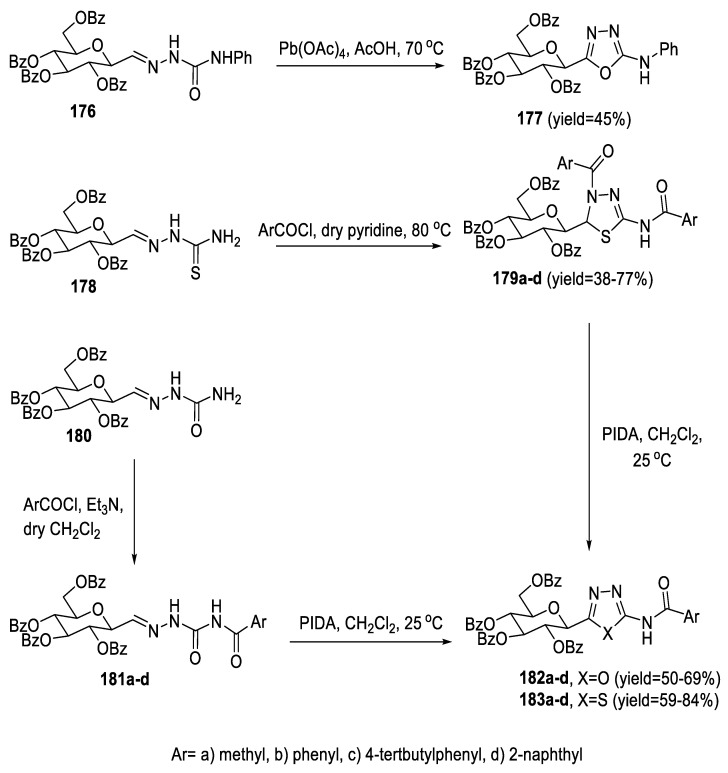
Synthetic pathway for compounds **177**, **182a–d**, and **183a–d**.

The 2-(β-D-glucopyranosyl)-5-(substituted-amino)-1,3,4-oxa-/thiadiazoles were formed by the oxidative ring closure of (semi)thiosemicarbazones. Semicarbazone **176** was reacted with Pb(OAc)_4_ in glacial AcOH to furnish 1,3,4-oxadiazole **177**. On the other hand, acylation of thiosemicarbazone **178** in dry pyridine resulted in the formation of 1,3,4-thiadiazolines (**179a–d**) as inseparable mixtures of two diastereoisomers in moderate yields. Furthermore, semicarbazone **180** was treated by acid chlorides in CH_2_Cl_2_ in the presence of Et_3_N to give intermediates (**181a–d**), which were then reacted with phenyliodine (III) diacetate (PIDA) in CH_2_Cl_2_ to obtain 1,3,4-oxadiazoles (**182a–d**) in good yields. Under these conditions, no reaction took place with thiosemicarbazone **178.** However, 1,3,4-thiadiazoles (**183a–d**) were directly derived in moderate yields from thiadiazolines (**179a–d**) by oxidation (Scheme 40) [90].

### 2.5. Synthesis from Acid/Thionyl Hydrazide

**Figure 17:**
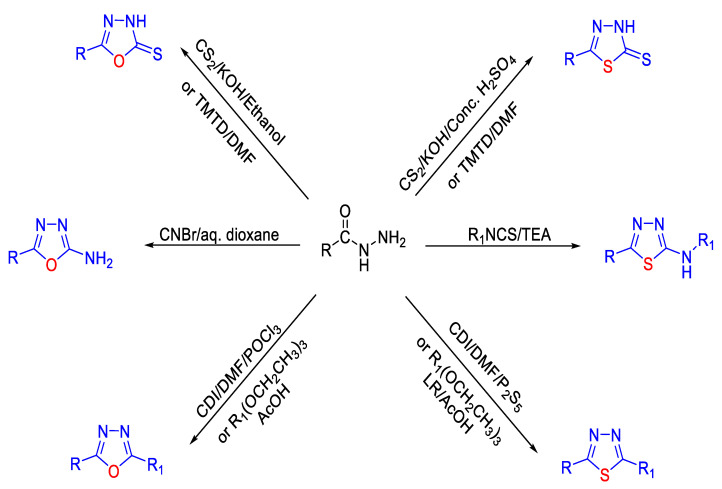
Different methods used for the synthesis of 1,3,4-oxa-/thiadiazoles from acid hydrazides.

To date, various methods have been reported in the literature for generating 1,3,4-oxa-/thiadiazoles from acid hydrazides (Figure 17). 1,3,4-Oxadiazoles can be synthesized from acid hydrazides through treatment with tetramethylthiuram disulfide (TMTD) in DMF [91], cyanogen bromide (CNBr) in aq. Dioxane [91], coupling agent (CDI)/THF/POCl_3_ [92], CS_2_/KOH in ethanol [93], or by reaction with triethyl orthoesters in glacial acetic acid [53]. On the other hand, 1,3,4-thiadiazoles can be synthesized through treatment of acid hydrazide with TMTD/DMF [91], isothiocyanates/TEA [91], CDI/DMF/P_2_S_5_ [92], by treatment with conc. H_2_SO_4_ through the formation of acyl thiosemicarbazide intermediate [93], or by reaction with triethylorthoesters/LR in glacial acetic acid [53]. Furthermore, TiCl_4_-mediated facile synthesis [94] and the palladium-catalyzed one-pot method [95] are used for the synthesis of 1,3,4-oxa-/thiadiazoles from acid/thionyl hydrazide.

**Scheme 41:**
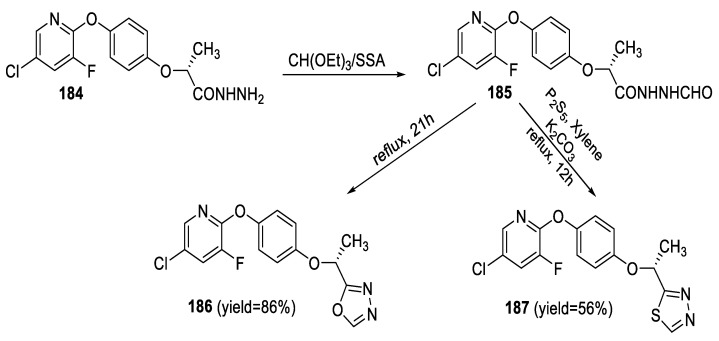
Synthetic pathway for compounds **186** and **187**.

A series of 1,3,4-oxadiazole and 1,3,4-thiadiazole bearing an (R) 5-(1-(4-(5- chloro-3-fluoropyridin-2-yloxy)phenoxy)ethyl) unit were synthesized from the corresponding acid hydrazide through the formation of the intermediate formylhydrazide**.** Prolonged reflux of hydrazide **184** in ethylorthoformate generated formylhydrazide **185** with subsequent cyclization to oxadiazole **186.** Alternatively, sulfurative cyclization of formylhydrazide **185** to thiadiazole **187** was affected by heating with P_2_S_5_ in xylene under reflux conditions. Oxadiazole was formed in a higher yield than thiadiazole (Scheme 41) [74].

**Scheme 42:**
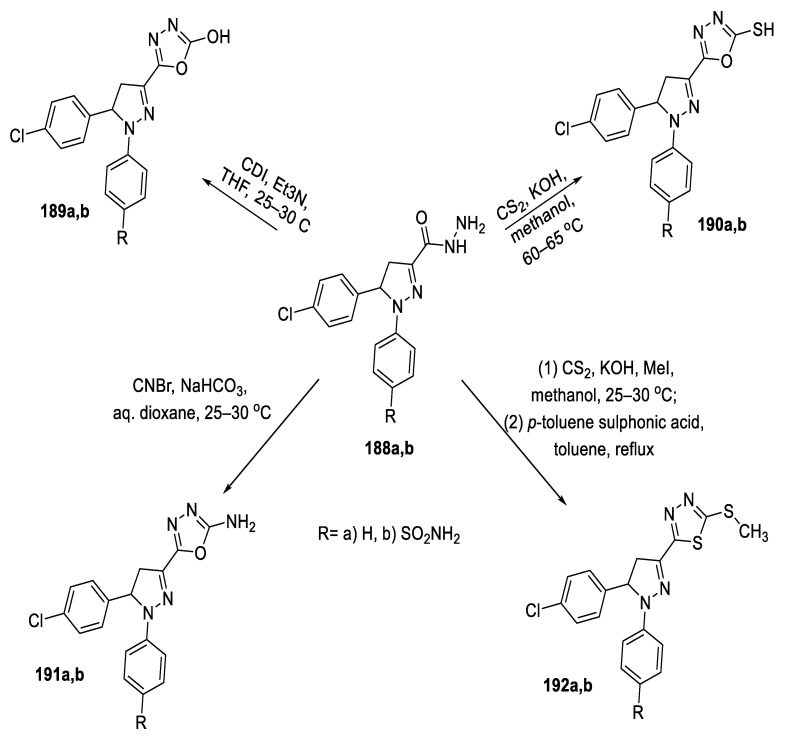
Synthetic pathway for compounds **189a,b**–**192a,b**.

A series of 4,5-dihydro-1,5-diaryl-1*H*-pyrazole substituted at position 3 with functionalized 1,3,4-oxadiazole or 1,3,4-thiadiazole ring was synthesized from carbohydrazide precursors (**188a,b**) using variable reagents and conditions. The 2-hydroxy-1,3,4-oxadiazoles (**189a,b**) were prepared by reaction of carbonyldiimidazole with (**188a,b**) in tetrahydrofuran using triethylamine as a base. The 1,3,4-oxadiazole-2-thiols (**190a,b**) were obtained by reaction of (**188a,b**) with carbon disulfide employing KOH as a base catalyst. The l,3,4-oxadiazole-2-amines (**191a,b**) resulted from the reaction of cyanogen bromide and NaHCO_3_ with precursors (**188a,b**). The 5-methylthio-1,3,4-thiadiazoles (**192a,b**) were synthesized by reaction of hydrazide (**188a,b**) with carbon disulfide and KOH followed by methylation using methyl iodide. These compounds were further cyclized in refluxing toluene using *p*-toluene sulfonic acid (Scheme 42) [96].

**Scheme 43:**
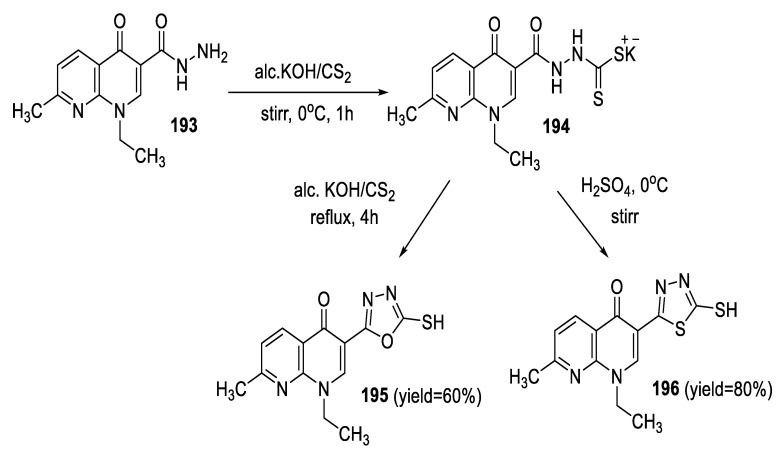
Synthetic pathway for compounds **195** and **196**.

Aggarwal et al., synthesized nalidixic acid-based 1,3,4-(oxa)thiadiazoles through the reaction of nalidixic acid hydrazide **193** with ice-cooled carbon disulfide in the presence of potassium hydroxide to produce potassium dithiocarbazinate intermediate **194.** Dithiocarbazinate intermediate **194** was then cyclized in acidic or in basic medium forming 1,3,4-thiadiazole **196** or 1,3,4-oxadiazole **195**, respectively. Thiadiazole was formed in a higher yield than its oxadiazole analog (Scheme 43) [93].

**Scheme 44:**
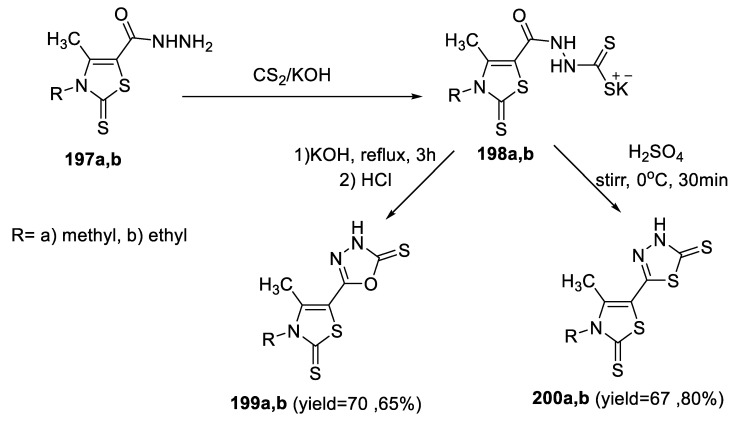
Synthetic pathway for compounds **199a,b** and **200a,b**.

Similarly, Knyazyan et al., synthesized 3*H*[1,3,4] (oxa)thiadiazole-2-thiones (**199a,b** and **200a,b**) under base- and acid-catalyzed heterocyclization of 2-(3-alkyl-4- methyl-2-thioxo-2,3-dihydro-thiazole-5-carbonyl)-hydrazinecarbodithioic acid potassium salts (**198a,b**) (Scheme 44) [97].

**Scheme 45:**
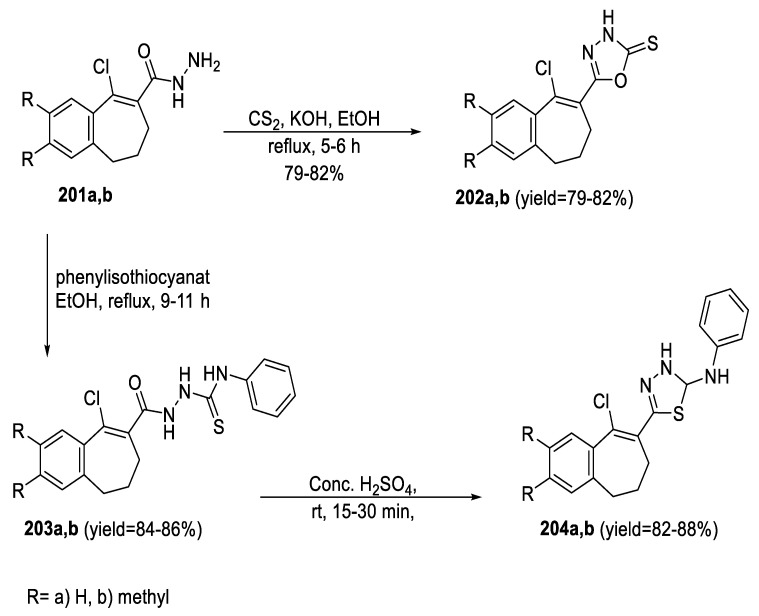
Synthetic pathway for compounds **202a,b** and **204a,b**.

A series of benzosuberone analogs embedded with 1,3,4-oxadiazole or 1,3,4-thiadiazole moieties were synthesized in high yields. The reaction of the hydrazides (**201a,b**) with phenyl isothiocyanate in ethanol gave the corresponding acylthiosemicarbazide (**203a,b**), which on treatment with conc. sulphuric acid yielded the required 1,3,4-thiadiazoles (**204a,b**), while oxadiazole analogs (**202a,b**) were obtained from (**201a,b**) directly by refluxing with carbon disulfide in the presence of alcoholic potassium hydroxide (Scheme 45) [98].

**Scheme 46:**
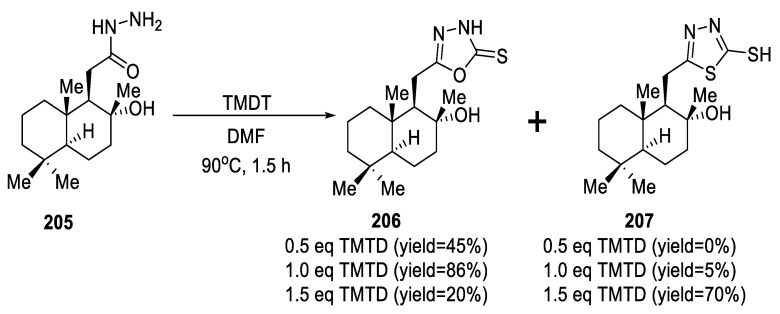
Synthetic pathway for compounds **206** and **207**.

In 2020, (+)-sclareolide-based homodrimane sesquiterpenoids bearing 1,3,4-oxa-/thiadiazole-2-thiols (**206**,**207**) were synthesized from acetohydrazide **205** through treatment with TMTD in DMF. Products were obtained selectively or in a mixture where the yield ratio between oxadiazole **206** and thiadiazole **207** was dependent on the amount of TMTD used in the reaction (Scheme 46) [91].

**Scheme 47:**
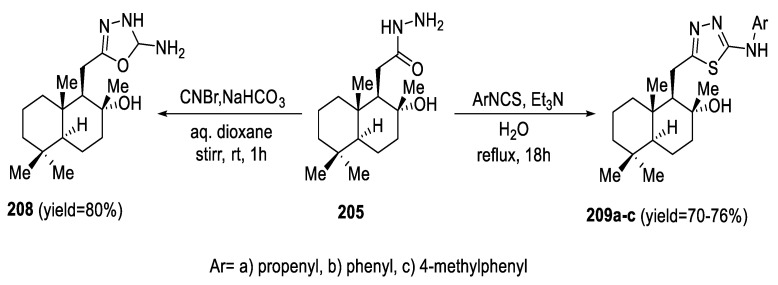
Synthetic pathway for compounds **208** and **209a–c**.

To obtain 2-aminosubstituted 1,3,4-thiadiazole and 1,3,4-oxadiazole units, homodrimane hydrazide **205** was reacted with isothiocyanate derivatives, without any isolations of the intermediate compounds, in the presence of Et_3_N in H_2_O, giving substituted 2-amino-1,3,4-thiadiazoles (**209a–c**) in moderate yields. Additionally, acetohydrazide **205** was treated with CNBr in aqueous dioxane to form unsubstituted 1,3,4-oxadiazol-2-amine **208** in an 80% yield (Scheme 47) [91].

**Scheme 48:**
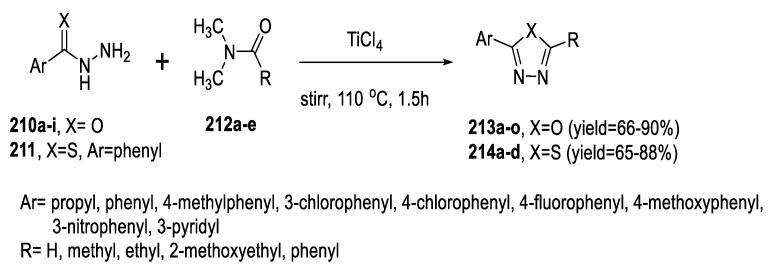
Synthetic pathway for compounds **213a–o** and **214a–d**.

In 2019, Zhang et al. developed an efficient synthesis of 2,5-disubstituted 1,3,4-oxadiazoles (**213a–o**) and 1,3,4-thiadiazoles (**214a–d**) from the reaction of acid hydrazides (**210a–i**) or thionyl hydrazide **211** with *N*,*N*-dimethyl amide derivatives (**212a–e**) utilizing TiCl_4_ as a single dehydration reagent for both coupling and cyclodehydration events. This protocol is a facile and economical method that provides the target oxadiazoles/thiadiazoles in good yields and with a wide substrate scope (Scheme 48) [94].

**Scheme 49:**
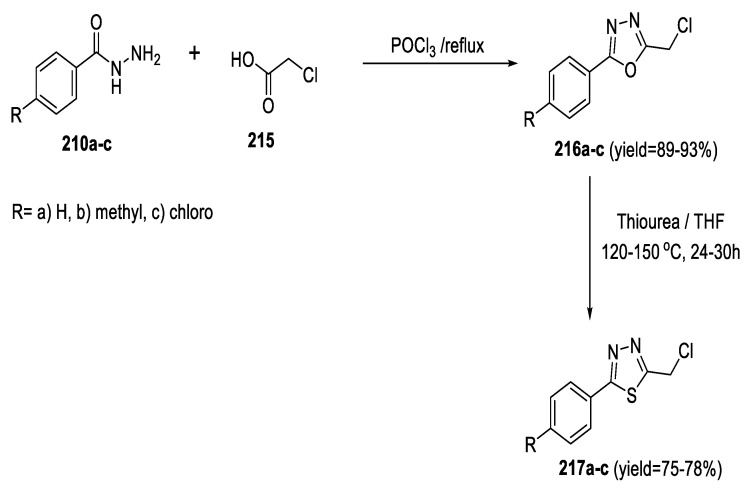
Synthetic pathway for compounds **216a–c** and **217a–c**.

In 2011, Padmavathi et al., prepared 5-aryl-2-(chloromethyl)-1,3,4-oxadiazoles (**216a–c**) by the reaction of chloroacetic acid **215** with benzoic hydrazides (**210a–c**) in the presence of POCl_3_. However, they obtained the 5-aryl-2-(chloromethyl)-1,3,4-thiadiazoles (**217a–c**) by the interconversion of compounds (**216a–c**) with thiourea in THF (Scheme 49) [99].

**Scheme 50:**
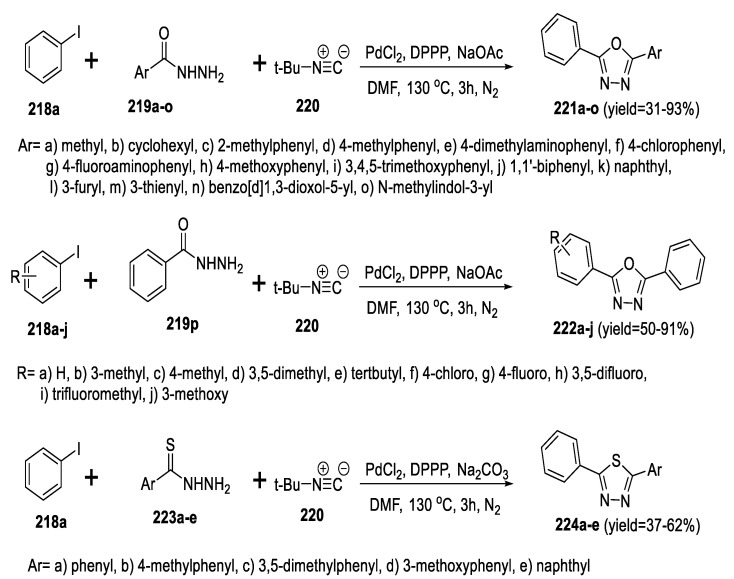
Synthetic pathway for compounds **221a–o**, **222a–j** and **224a–e**.

Xiang et al., reported an efficient one-pot Pd-catalyzed carbonylative coupling reaction for the synthesis of 1,3,4-oxadiazoles (**221a–o**, **222a–j**) from the reaction of benzohydrazides (**219a–p**) and iodobenzene (**218a–j**) with tert-butyl isocyanide **220** via isocyanide insertion/cyclization sequential reaction. The optimal reaction conditions were PdCl_2_ and DPPP as the catalyst systems, with NaOAc as the base and DMF as the solvent, which furnished the products in moderate yields. Obtaining 1,3,4-thiadiazoles (**224a–e**) required replacement of benzohydrazides (**219a–o**) with benzothiohydrazides (**223a–e**), as well as changing the base from NaOAc to Na_2_CO_3_ (Scheme 50) [95].

**Scheme 51:**
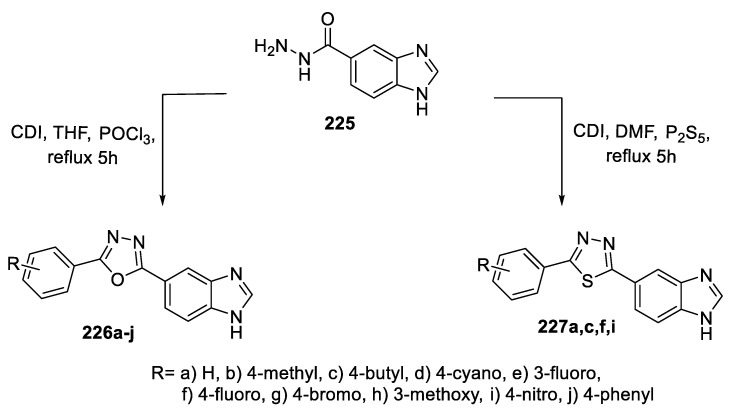
Synthetic pathway for compounds **226a–j** and **227a,c**,**f**,**i**.

A series of benzimidazole derivatives bearing a 1,3,4-oxa-/thiadiazole core were synthesized by the precursor carbohydrazide **225** with substituted benzoic acid using the coupling agent CDI and POCl_3_ and P_2_S_5_ as cyclizing agents to yield the oxadiazole (**226a–j**) and thiadiazole (**227a,c**,**f**,**i**) cores, respectively (Scheme 51) [92].

**Scheme 52:**
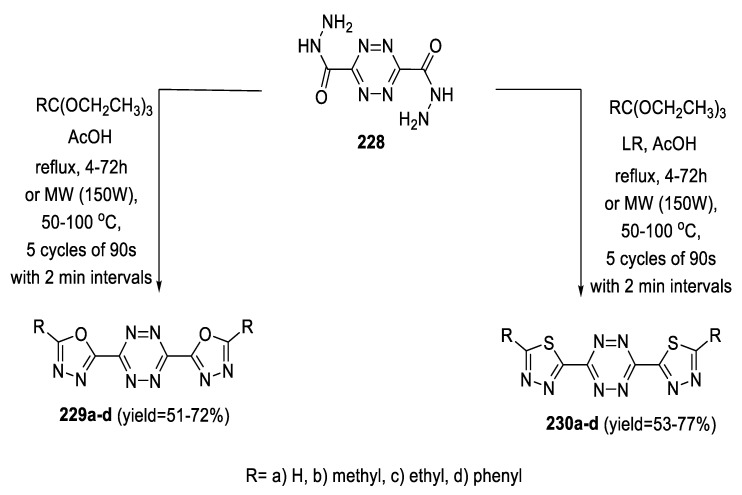
Synthetic pathway for compounds **229a–d** and **230a–d**.

In 2020, s-tetrazine-1,3,4-oxadiazole hybrids (**229a–d**) were obtained by reaction of s-tetrazine-3,6-dicarbohydrazide **228** and triethylorthoesters in glacial acetic acid via conventional heating. S-tetrazine-1,3,4-thiadiazoles (**230a–d**) were synthesized by the same method with the addition of LR. The products were also obtained by microwave irradiation in similar yields, but in a very short reaction time (Scheme 52) [53].

## 3. Biological Activities

**Figure 18:**
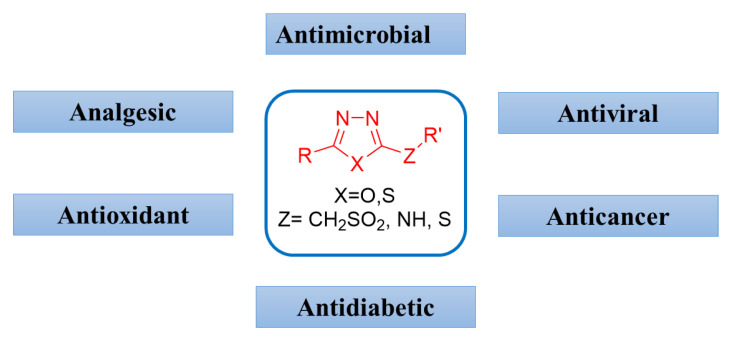
Some biological activities for 1,3,4-oxa-/thiadiazoles.

Over the past few decades, many 1,3,4-oxadiazoles and 1,3,4- thiadiazoles have been intensively studied for their broad biomedical applications, such as antimicrobial, antiviral, anticancer, antidiabetic, antioxidant, and analgesic activities (Figure 18).

### 3.1. Antimicrobial Activity

In 2020, thirteen novel sclareolide-based homodrimane sesquiterpenoids bearing 1,3,4-oxadiazole or 1,3,4-thiadiazole moieties were synthesized and demonstrated promising antifungal and antibacterial activities toward fungal species *Aspergillus niger*, *Fusarium solani*, *Penicillium chrysogenum*, *Penicillium frequentans*, and *Alternaria alternata* and Gram-positive *Bacillus* species and Gram-negative *Pseudomonas aeruginosa* bacteria strains. On the basis of the SAR correlations, it was noticed that compounds having a 1,3,4-thiadiazole moiety were more active compared with those having a 1,3,4-oxadiazole moiety.

**Figure 19:**
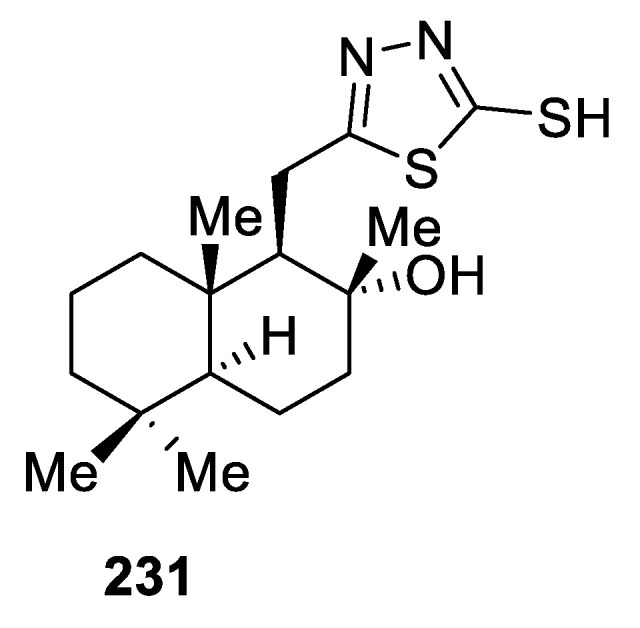
Structure of compound **231**.

Among the thiadiazole derivatives, compound **231** (Figure 19), featuring the 2-mercapto group, was the most potent, with promising broad-spectrum antifungal (MIC 0.032 µg/mL) and antibacterial (MIC 0.094 µg/mL) activities [91].

**Figure 20:**
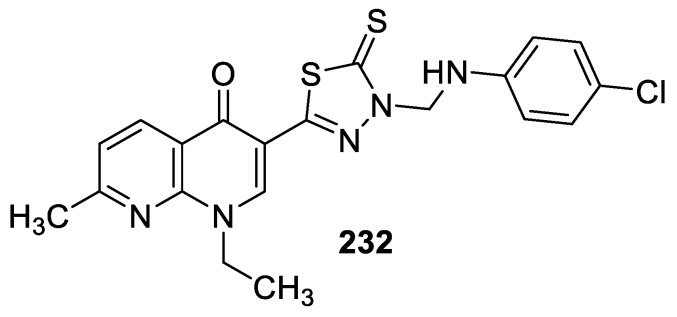
Structure of compound **232**.

In 2012, nalidixic acid-based 1,3,4-(oxa)thiadiazol-2-thione, their Mannich bases, S-alkylated/arylated sulfides, and sulfones were synthesized and screened against Gram-positive bacteria (*S. aureus* and *B. subtilis*) and Gram-negative bacteria (*E. coli*, *P. aeruginosa*, and *K. pneumonia*) using Streptomycin as the reference standard drug. According to evaluation results, the Mannich base **232** (Figure 20) emerged as a potent antibacterial agent against all test microorganisms except *E. coli*, with an MIC range of 6.25–12.5 μg/mL [93].

A series of 1,3,4-oxadiazoles/thiadiazoles bearing pyrazole scaffolds were screened for their in vitro antimicrobial activity against *S. aureus*, *E. coli*, *P. aeruginosa*, *A. niger*, and *A. flavus* bacteria, and *C. albicans* fungal species using Ciprofloxacin and Fluconazole as reference drugs. The tested compounds showed moderate to excellent antibacterial and antifungal activities. The chloro-substituted oxadiazole **169d** was equipotent to reference drugs Ciprofloxacin and Fluconazole MIC 12.5–25 μg/mL, while the chloro-substituted 1,3,4-oxadiazole **169j** showed greater activity against Gram-negative *E. coli* and *C. albicans* fungi (MIC 12.5 μg/mL) than Ciprofloxacin and Fluconazole (MIC 25 and 50 μg/mL, respectively). On the other hand, the chloro-substituted thiadiazoles (**170d**,**j**) showed a higher activity against Gram-positive *S. aureus* (MIC 12.5 μg/mL) compared with standard Ciprofloxacin (MIC 25 μg/mL). Moreover, **170j** was more potent against Gram-negative *E. coli* (MIC 12.5 μg/mL) than Ciprofloxacin (MIC 25 μg/mL).

**Figure 21:**
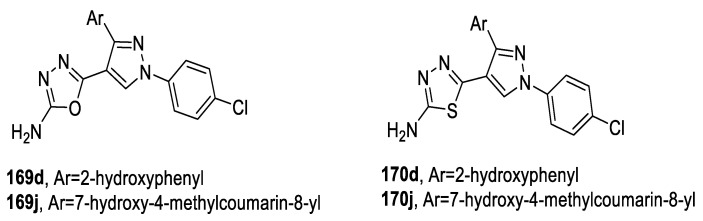
Structures of compounds **169d**,**j** and **170d**,**j**.

The comparative analysis illustrated that, among oxadiazoles and thiadiazoles, the sulfur moiety in 1, 3, 4- thiadiazoles acts as a potent inhibitor, while compounds containing moderately electronegative elements such as sulfur and chlorine (**170d**,**j**) showed better in vitro activity than compounds with more electronegative elements, such as oxygen (**169d**,**j**) (Figure 21) [84,89].

**Figure 22:**
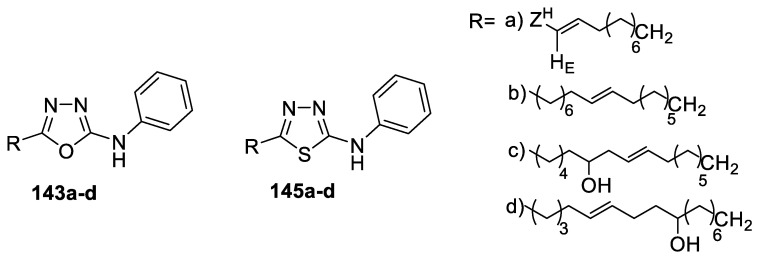
Structures of compounds **143a–d** and **145a–d**.

Long-chain 5-alkenyl/hydroxyalkenyl-2-phenylamine-1,3,4-oxadiazoles and thiadiazoles were synthesized and screened as antimicrobial agents against *S. pyogenes*, *S. aureus*, *P. aeruginosa*, *K. pneumoniae*, and *E. coli* bacterial strains and *C. albicans*, *A. fumigatus*, *T. mentagrophytes*, and *P. marneffei* fungal strains. The study results showed no difference in activity between the bioisosteres of oxadiazoles and thiadiazoles. Examination of SAR indicated that the presence of a hydroxyalkyl chain at the fifth position of the oxa-/thiadiazole ring increased the antibacterial activity in compounds **143c**, **143d**, **145c**, and **145d**, as they were equally as potent as the reference drug chloramphenicol (MIC = 6.25 μg/mL). Moreover, the compounds with an internal double bond in the long alkenyl substituent of synthesized oxa-/thiadiazoles **143b** and **145b** were found to be potent antifungal agents (Figure 22) [64].

**Figure 23:**
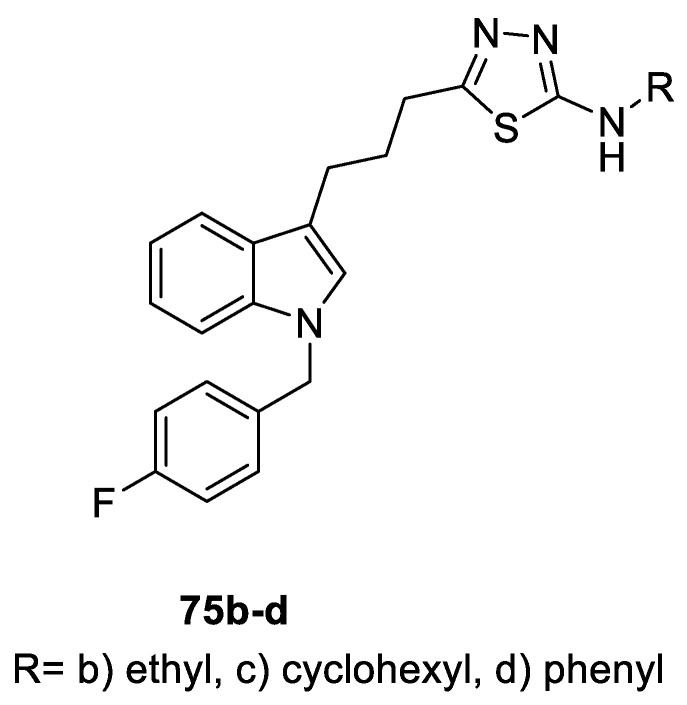
Structure of compounds **75b-d**.

A series of 5-[*N*-(*p*-flurobenzyl)indolyl]propyl-1,3,4-(oxa)thiadiazoles were tested in vitro for their antibacterial and antifungal activities. Only the thiadiazole derivatives showed promising results. The 2-phenylamino derivative **75d** was more potent than the reference drug Gentamicin against Gram-negative *E. coli*, with MIC = 0.24 and 3.9 μg/mL, respectively. The 2-ethylamino analog **75b** was equipotent to Gentamicin against *E. coli* (MIC = 3.9 μg/mL), and was four times more potent than Gentamicin against *P. aeruginosa* (MIC 3.9 μg/mL and 15.6 μg/mL, respectively). The 2-cyclohexyl amino counterpart **75c** showed promising results against *E. coli* (MIC 0.49 μg/mL) compared to Gentamicin and displayed equivalent potency to Amphotericin B against the *S. racemosum* fungus (MIC = 3.9 μg/mL) (Figure 23) [61].

**Figure 24:**
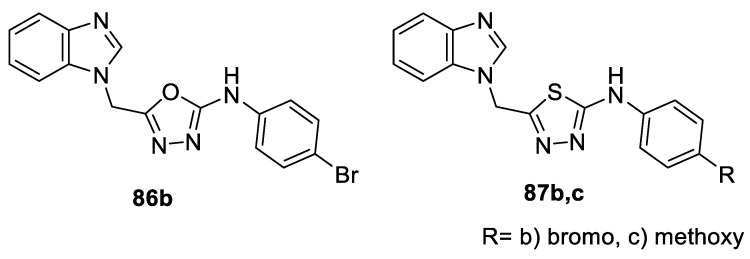
Structures of compounds **86b** and **87b**,**c**.

The potential antimicrobial effect of 5-(benzimidazol-1-yl methyl)-2-arylamino-1,3,4-(oxa)thiadiazoles was investigated against microorganisms representing Gram-positive bacteria (*S. aureus* and *S. epidermidis*), Gram-negative bacteria (*E. coli* and *P. aerugnosa*), and fungi (*C. albicans*). The oxadiazole derivative **86b** (inhibition zone diameter (IZD) = 26–28 mm), as well as the thiadiazole derivative **87b** (IZD =25–27 mm), carrying bromophenyl amino rest, and the methoxyphenyl amino thiadiazole **87c** (IZD = 26–28 mm) showed comparable antibacterial activity to the reference drug Cefotaxime (IZD = 28–35 mm) against the tested strains. Additionally, they elicited superior antifungal activity (IZD = 28–30 mm) compared to Nystatin (IZD = 24 mm) (Figure 24) [71].

**Figure 25:**
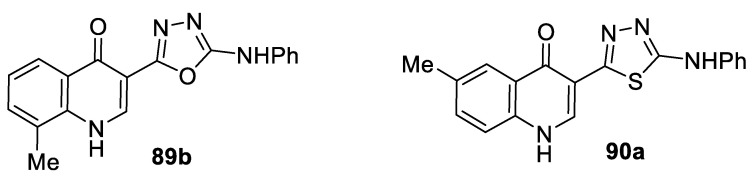
Structures of compounds **89b** and **90a**.

Screening the antibacterial activity of 5-(arylamino)-1,3,4-(oxa)thiadiazol-2-yl)quinolin-4-one derivatives against three different bacterial species, namely, Gram-negative *P. aerugnosa*, Gram-positive *B. subtilis*, and actinomycete *Streptomyces* sp., revealed variable degrees of inhibitory actions. The highest degrees of inhibition were recorded for the oxadiazole compound **89b** and the thiadiazole compound **90a**, and they were equipotent. The most susceptible organism was *B. subtilis* (IZD = 20–22 mm), followed by *Streptomyces* sp. (IZD = 12–18 mm), whereas the lowest inhibitory effect was encountered in the case of *Pseudomonas* sp. (IZD = 4 mm) (Figure 25) [72].

**Figure 26:**
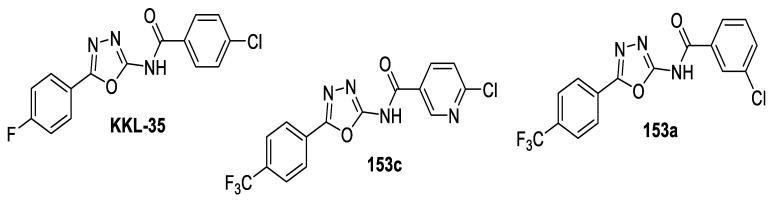
Structures of compounds **KKL-35** and **153a,c**.

Several 1,3,4-oxa-/thiadiazole derivatives were designed and synthesized as analogs for 4-chloro-*N*-(5-(4-fluorophenyl)-1,3,4-oxadiazol-2-yl)benzamide **KKL-35**, which is one of the most active oxadiazole compounds, showing potent bactericidal activity against various non-related bacteria [100]. The designed compounds were tested for antibacterial activity against a selection of 24 bacterial species, using **KKL-35** as a reference. 6-Chloro-*N*-(5-(4-(trifluoromethyl)phenyl)-1,3,4-oxadiazol-2-yl)nicotinamide **153c**, and its 3-chlorobenzamide analog **153a** exhibited a wide spectrum of action and strong antimicrobial activity. They were active against almost all of the tested Gram-positive strains except *C. perfringens* and *S. pyogenes*. However, the only Gram-negative strain affected was *B. fragilis* (by **153c**). They also affected the growth of the mycobacterium, *M. fortuitum*, and **153c** had an effect against *M. abscessus*. Compared to **KKL-35**, **153c** displayed lower MICs for *E. faecalis* (MIC 2–4 mg/L), *E. faecium* (MIC 2 mg/L), and *M. fortuitum* (MIC 8 mg/L). In addition, **153a** showed a superior effect against *E. faecalis*, *E. faecium*, and *S. epidermidis* (MICs = 0.0625–1 mg/L). Furthermore, these compounds show synergistic activity with conventional antibiotics and have low toxicities (Figure 26) [87].

**Figure 27:**
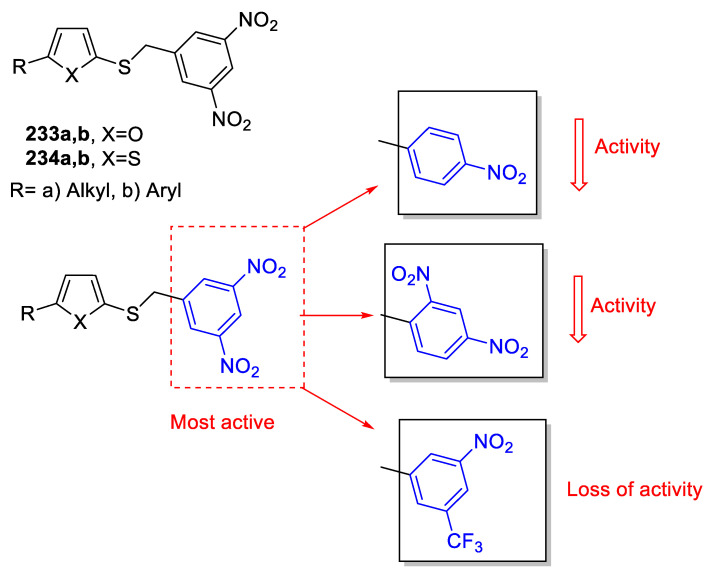
SAR for compounds **233** and **234**.

Karabanovich et al., evaluated the activity of a series of substituted benzylsulfanyl-1,3,4-oxadiazoles and 1,3,4-thiadiazoles as antitubercular agents. The majority of these compounds exhibited outstanding in vitro activity against *Mycobacterium tuberculosis*. The activity results revealed that there was no difference in activity between oxadiazoles and thiadiazole analogs. Most of the compounds bearing the 3,5-dinitrobenzylsulfanyl moiety (**233** and **234** series) exhibited excellent activity against drug-susceptible and multidrug-resistant M.tb. strains, with no cross-resistance with first- and second-line anti-TB drugs. An SAR study indicated that removal or substitution of one nitro group or changing the positions of both nitro groups resulted in a decrease or loss of activity (Figure 27). The anti-TB effect of the compounds was selectivity with low toxicity, genotoxicity, and mutagenicity [101].

**Figure 28:**
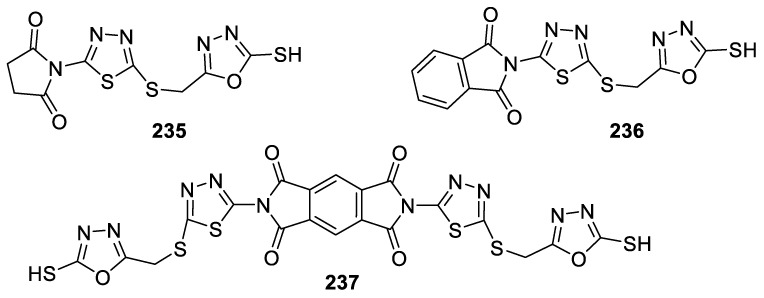
Structures of compounds **235**–**237**.

According to the theory that the incorporation of different pharmacophores into a single molecule can change the activity of the newly obtained compound, a series of cyclic imides containing both 1,3,4-oxadiazole and 1,3,4-thiadiazole heterocycles was prepared and tested for their antimicrobial activity against four strains of bacteria (*S. aureus*, *S. pyogenes*, *E. coli*, and *P. aeruginosa*) and *C. albicans* fungi. The results indicated that compounds **235** (IZD = 14–20.5 mm), **236** (IZD = 12.6–21 mm), and **237** (IZD = 12.9–20.1 mm) were highly active against all types of tested bacteria compared to Ampicillin (IZD = 12–17 mm). Only compounds **235** (IZD = 18.6 mm) and **237** (IZD = 22.2 mm) were also highly active against *C. albicans* fungi (Fluconazole IZD = 18 mm) (Figure 28) [102].

**Figure 29:**
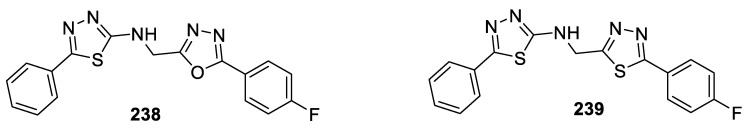
Structures of compounds **238** and **239**.

This study supports the same theory, whereby the potential antibacterial activity of a series of 1,3,4-thiadiazole derivatives tagged to oxadiazole and thiadiazole units were evaluated against *E. coli*, *S. typhimurium*, *L. monocytogenes*, *K. pneumonia*, *S. typhi*, *S. aureus*, and *B. subtilis* species. The assay results revealed that compounds **238** and **239**, with a strong electron-withdrawing fluorine atom at the para position, were more potent than Ampicillin on all tested strains (MIC = 62.5–100 μg/mL and 100–250 μg/mL, respectively). It was also noticed that the 1,3,4-thiadiazoles linked with 1,3,4-thiadiazole units **239** were more active than 1,3,4-thiadiazoles linked with 1,3,4-oxadiazole **238** (Figure 29) [103].

**Figure 30:**
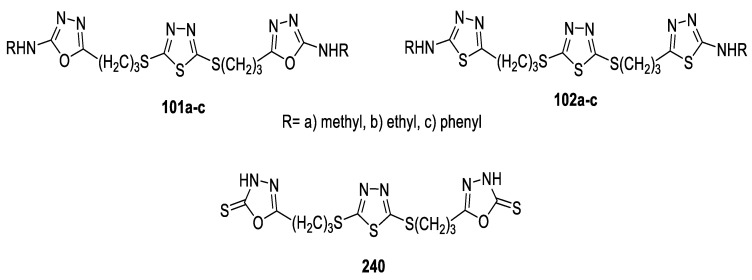
Structures of compounds **101a–c**, **102a–c** and **240**.

A series of 2,5-disubstituted-1,3,4-thiadiazole tethered 1,3,4-(oxa)thiadiazole derivatives were synthesized as antimicrobial agents. Their activities were evaluated against a panel of standard strains of pathogenic microorganisms, including Gram-positive bacteria (*S. pneumonia*, *B. subtilis*, and *S. aureus*), Gram-negative bacteria (*P. aeruginosa*, *E. coli*, and *K. pneumonia*), and fungi (*A. fumigatus*, *C. albicans*, and *G. candidum*), and against reference drugs Ciprofloxacin and Fluconazole. The antimicrobial activity of the thiadiazoles (**102a–c**) revealed that the tested compounds showed promising activity against all bacterial and fungal strains at 8–31.25 μg/mL. However, the oxadiazole isosteres (**101a–c**) showed greater antibacterial activity against Gram-positive bacteria, at MIC = 8–16 μg/mL, but they exhibited lower antifungal activity (MIC = 31.25–62.5 μg/mL). Oxadiazole **240** functionalized with a thiol group at position 2 exhibited excellent antibacterial activities against all bacterial strains at MIC = 4–8 μg/mL and good activity towards fungal strains at MIC =16–31.28 µg/mL (Figure 30) [76].

**Figure 31:**
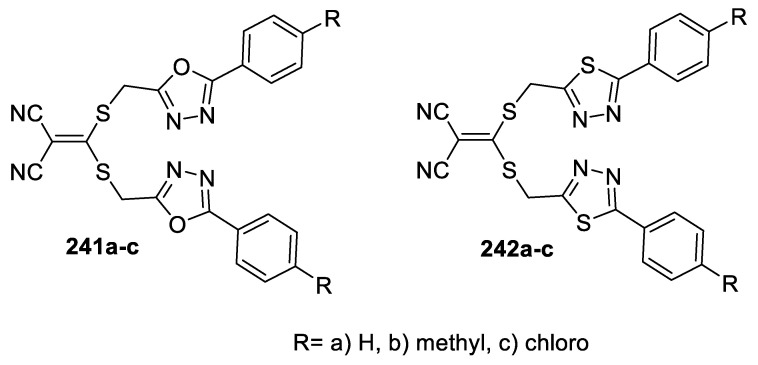
Structures of compounds **241a–c** and **242a–c**.

Antimicrobial activity screening of 2-(bis((1,3,4-oxadiazolyl/1,3,4-thiadiazolyl) methylthio)methylene)malononitrile derivatives (**241a–c**, **242a–c**) against *S. aureus*, *B. subtilis* (Gram-positive bacteria) and *E. coli*, *K. pneumoniae* (Gram-negative bacteria) revealed that compounds having a thiadiazole moiety (**242a–c**) exhibited high activity against both Gram-positive and Gram-negative bacteria (IZD = 17–35 mm). However, the oxadiazole compounds (**241a–c**) displayed moderate activity towards Gram-positive bacteria (IZD = 10–20 mm). Similarly, compounds having thiadiazole rings (**242a–c**, IZD = 18–34 mm) exhibited higher antifungal activity than those with an oxadiazole moiety (**241a–c**, IZD = 12–22 mm) against *C. albicans*, *A. niger*, and *F. solani* (Figure 31) [99].

**Figure 32:**
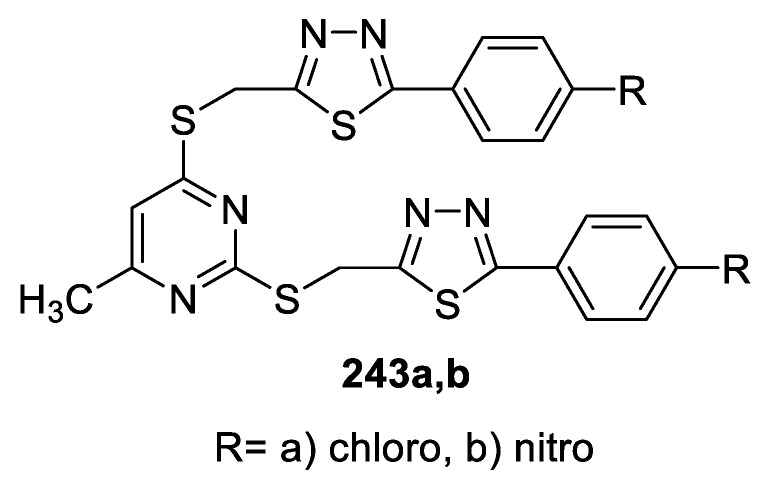
Structure of compounds **243a,b**.

Sekhar et al., prepared new classes of methylthio-linked pyrimidinyl 1,3,4-oxadiazoles and 1,3,4-thiadiazoles. Screening the antibacterial activity indicated that the title compounds were more effective against Gram-negative bacteria than Gram-positive ones. The pyrimidinyl bis-thiadiazoles **243** exhibited greater activity than pyrimidinyl bis-oxadiazoles. The presence of an electron-withdrawing group on the aromatic ring increased the activity. The *p*-chlorophenyl **243a** and *p*-nitrophenyl **243b** derivatives (MIC 6.25 μg/well) were effective, particularly against *P. aeruginosa*, and were equipotent to Chloramphenicol (MIC 6.25 μg/well) (Figure 32) [104].

**Figure 33:**
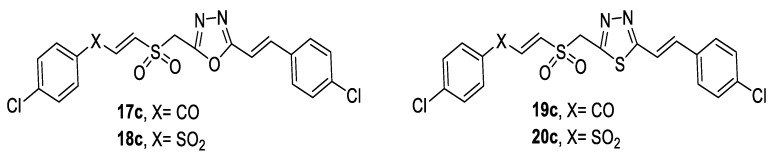
Structures of compounds **17c**–**20c**.

In 2019, a new class of aroylethenesulfonylmethyl/arylsulfonylethenesulfonylmethyl styryl 1,3,4-oxadiazoles, 1,3,4-thiadiazoles (Figure 33), was synthesized and evaluated for antimicrobial activity. It was observed that compounds having thiadiazole (**19c**, **20c**) moiety exhibited higher antimicrobial activity than those with the oxadiazole moiety (**17c**, **18c**). Additionally, arylsulfonylethenesulfonylmethyl styryl azoles (**18c**, **20c**) exhibited greater antimicrobial activity than aroylethenesulfonylmethyl styrylazoles (**17c**, **19c**). Moreover, compounds with an electron-withdrawing chloro substituent on the phenyl ring possessed enhanced activity [6].

**Figure 34:**
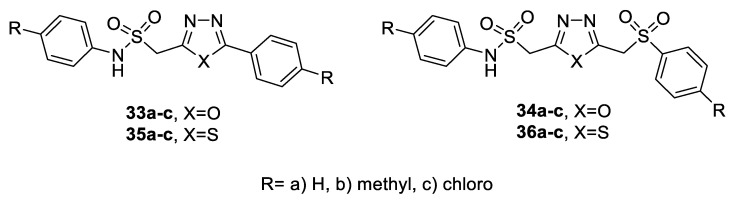
Structures of compounds **33a–c**–**36a–c**.

The potential activity of 2-(arylaminosulfonyl methyl)-1,3,4-oxa(thia)diazole derivatives (compounds **33a–c** to **36a–c**) as antimicrobial agents was screened in vitro against a panel of Gram-positive bacteria—*S. aureus*, *B. subtilis*—and Gram-negative bacteria—*K. pneumoniae* and *P. vulgaris* and fungi *F. solani*, *C. lunata*, and *A. niger*. The results showed that compounds with the thiadiazole moiety (**35a–c**, **36a–c**) exhibited higher antimicrobial activity than those with oxadiazole units (**33a–c**, **34a–c**). In particular, compounds (**7a–c**) carrying the thiadiazole moiety displayed potent activity against the tested Gram-positive bacteria and fungi (IZD > 26 mm) and good activity against Gram-negative bacteria (IZD > 20 mm). Thiadiazole derivatives featuring the 5-arylsulfonylmethane moiety (**36a–c**) displayed higher activity than their respective 5-aryl counterparts (**35a–c**) against all of the tested microorganisms. Additionally, the antimicrobial effect was enhanced upon grafting a Cl-substituent to the phenyl ring (Figure 34) [50].

**Figure 35:**
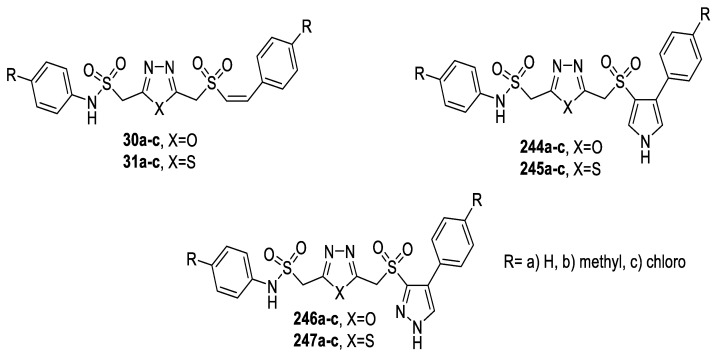
Structures of compounds **30a–c**, **31a–c** and **244a–c**–**247a–c**.

A series of 5-(styryl/pyrrolyl/pyrazolylsulfonylmethyl)-2-(arylaminosulfonylmethyl) 1,3,4-oxa(thia)diazoles (Figure 35) were prepared and tested for antimicrobial activity against *S. aureus*, *B. subtilis* (Gram-positive bacteria), *P. aeruginosa* and *K. pneumoniae* (Gram-negative bacteria). The data showed that the presence of a chloro substituent on phenyl rings of thiadiazoles (**31c**, **245c**, **247c**) enhanced the activity against the tested bacteria. The corresponding oxadiazole analogs (**30a–c**, **244a–c**, **246a–c**) proved to be less potent antimicrobial agents. It was also observed that the styryl derivatives (**30a–c**, **31a–c**) displayed higher potency than their pyrrolyl/pyrazolyl counterparts (**244a–c**–**247a–c**) [49].

**Figure 36:**
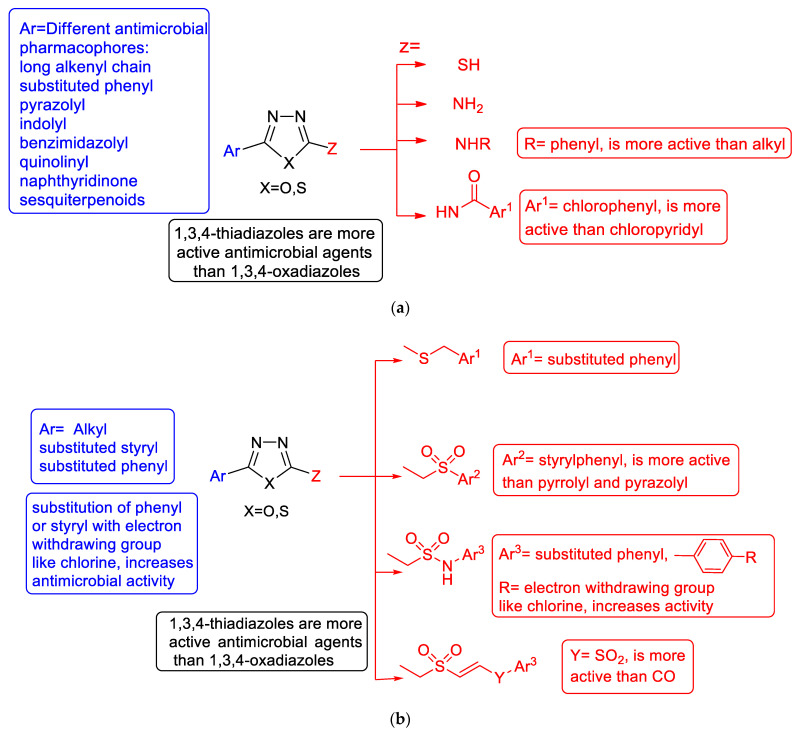
(**a**) Collective antimicrobial SAR for thioxo-, amino-, and amido-substituted 1,3,4-oxa-/thiadiazoles. (**b**) Collective antimicrobial SAR for arylsulfide-, sulfone-, and sulfonamide-substituted 1,3,4-oxa-/thiadiazoles.

An extract from the antimicrobial structure–activity relationship of substituted 1,3,4-oxa-/thiadiazoles is illustrated in (Figure 36a,b.)

### 3.2. Antiviral Activity

**Figure 37:**
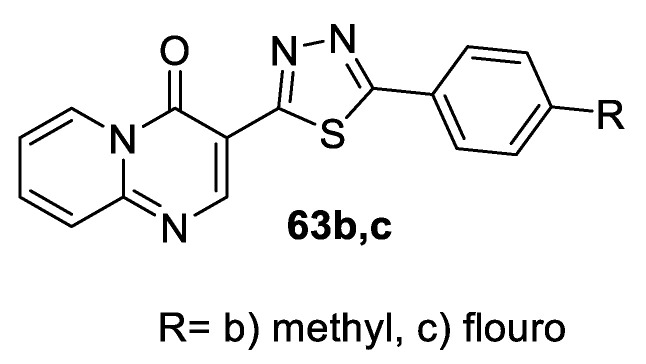
Structure of compounds **63b**,**c**.

A series of 4-oxo-4H-pyrido[1,2-a]pyrimidine derivatives containing 1,3,4-oxa(thia)diazole rings as a part of the metal chelation motif were screened for their in vitro anti-HIV-1 activity by determining their ability to inhibit the replication of HIV-1 in Hela cell cultures. All of the tested compounds were safe with no cytotoxicity at a concentration of 100 μM. Compounds containing thiadiazole rings were more potent than the corresponding oxadiazole analogs. The highest degrees of inhibition against HIV-1 (NL4- 3) were reported for the thiadiazoles **63b** and **63c**: 51% and 48%, respectively. The docking study revealed that the anti-HIV activity of these compounds might involve a metal chelating mechanism (Figure 37) [59].

**Figure 38:**
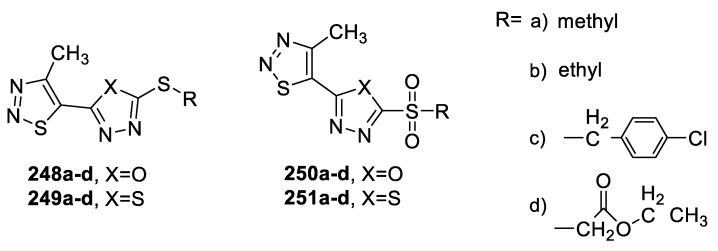
Structures of compounds **248a–d**–**251a–d**.

The antiviral activity of a series of thioether/sulfone compounds containing 1,2,3-thiadiazole and 1,3,4-oxa(thia)diazole rings was tested against tobacco mosaic virus TMV using Ningnanmycin as a reference drug. The data results revealed that the compounds containing 1,2,3-thiadiazole incorporating 1,3,4-oxadiazole ring **248a**, **248b**, **248c**, and **250a** showed equipotent activities to Ningnanmycin with curative effects ranging from 46.8% to 54.1%. However, a slight lowering of activity was noticed with the 1,3,4-thiadiazole isosteres (**249**,**251**). Oxidation of thioethers (**248**,**249**) to their corresponding sulfones (**250**,**251**) slightly reduced the values of antiviral activities (Figure 38) [105].

### 3.3. Anticancer Activity

**Figure 39:**
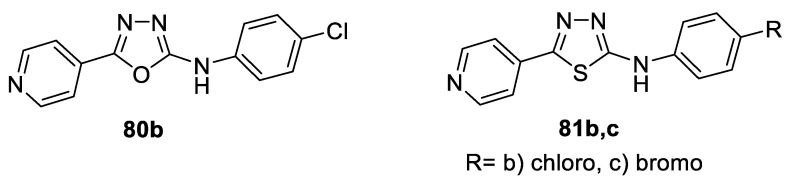
Structures of compounds **80b** and **81b**,**c**.

A series of N-(4-substitutedphenyl)-5-(pyridin-4-yl)-1,3,4-oxa(thia)diazol-2-amines were synthesized and evaluated with respect to their in vitro cytotoxicity against six human cancer cell lines, including cells derived from human gastric cancer (NUGC), human colon cancer (DLD1), human liver cancer (HA22T and HEPG2), nasopharyngeal carcinoma (HONE1), human breast cancer (MCF), and normal fibroblast cells (WI38) using CHS 828, a pyridyl cyanoguanidine, as a standard antitumor drug. The overall results revealed superior activity of 1,3,4-thiadiazoles compared to that of their 1,3,4-oxadiazoles bioisosteres, where only one oxadiazole compound **80b** showed selective moderate activity against NUGC and DLD1 cell lines. Among 1,3,4-thiadiazoles, only the compounds bearing 4- chlorophenyl (**81b**) and 4-bromophenyl **81c** pharmacophores were found to be active, which proves that the electronegativity of substituents plays an essential role in the activity. Compound (**81c**) showed nearly equipotent activity against the NUGC cell line compared to the standard CHS 828, with IC_50_ = 0.028 μM, while **81c** also showed 4-fold higher activity against HA22T and HEPG2 cell lines compared to its analog **81b** (IC_50_ = 0.180, 0.264 μM and 0.753, 0.970 μM, respectively) (Figure 39) [69].

**Figure 40:**
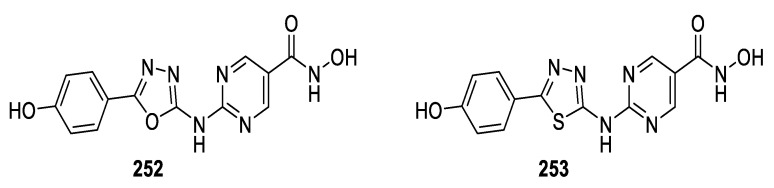
Structures of compounds **252** and **253**.

Rajak et al., prepared two series of hydroxamic acid-based 1,3,4-oxa-/thiadiazoles as histone deacetylase inhibitors and examined their antiproliferative activities in vitro using histone deacetylase inhibitory assay and MTT assay. They were also tested for antitumor activity against Ehrlich ascites carcinoma cells in Swiss albino mice. The results showed that among the oxadiazole series, compound **252** was the most potent, displaying the maximum HDAC inhibitory activity with an IC_50_ = 0.017 μM against HDAC-1 and an IC_50_ = 0.28 μM in HCT-116 cell proliferation assay, as well as %TWI = 85.7 and %TCI = 77.7 against Ehrlich ascites carcinoma cells in Swiss albino mice. In the thiadiazole series, compound **253** was the most potent, displaying the maximum HDAC inhibitory activity, with an IC_50_ = 0.018 μM against HDAC-1 and an IC_50_ = 0.31 μM in HCT-116 cell proliferation assay, and %TWI = 75.0; %TCI = 76.8 against Ehrlich ascites carcinoma cells. An overview of the results shows that thiadiazoles were found to be more active than the corresponding oxadiazole (Figure 40) [106].

**Figure 41:**
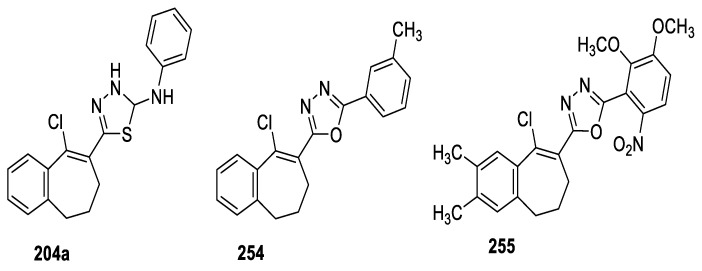
Structures of compounds **204a**, **254** and **255**.

The in vitro antiproliferative activity of a series of benzosuberones embedded with 1,3,4-oxa(thia)diazole moieties was examined against four human cancer cell lines (cervical He La, breast MDa–mB-231, pancreatic PANC1, and alveolar A549), where Paclitaxel, Nocodazole, Colchicine, Combretostatin, and Doxorubicin were used as standard drugs. The reported data showed that compounds **204a**, **254**, and **255** showed potent activity, with GI_50_ values ranging from 0.079 µM to 0.957 µM against the four human cancer cell lines. However, compound **254** was nearly equipotent to Colchicine against the HeLa cell line with GI_50_ = 0.079 µM. Based on the overall results, it was concluded that benzosuberones attached to oxadiazoles were more active than the corresponding thiadiazoles (Figure 41) [98].

**Figure 42:**
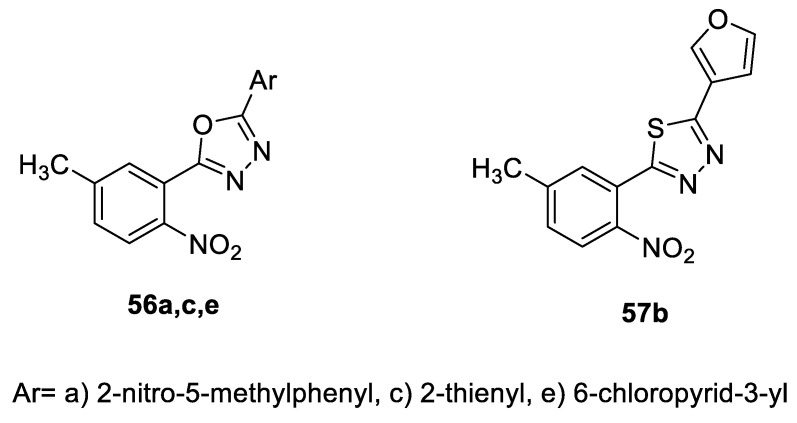
Structures of compounds **56a,c**,**e**, and **57b**.

The cytotoxic activities of a series of 2-(5-methyl-2-nitrophenyl)- 5-(substituted)-1,3,4-oxa(thia)diazoles were tested using Podophyllotoxin as a standard drug. The unsymmetrical 1,3,4-oxadiazole compounds with thiophene or chloropyridine substitution at the 5th position (**56c**, **56e**) and the 1,3,4-thiadiazole compound with furan substitution at the 5th position **57b** showed significant activity (IC_50_ = 10–19.45 µg/mL) compared to the standard podophyllotoxin (IC_50_ of 3.64 µg/mL), in contrast to the inactive symmetrical oxadiazole **56a** (Figure 42) [55].

**Figure 43:**
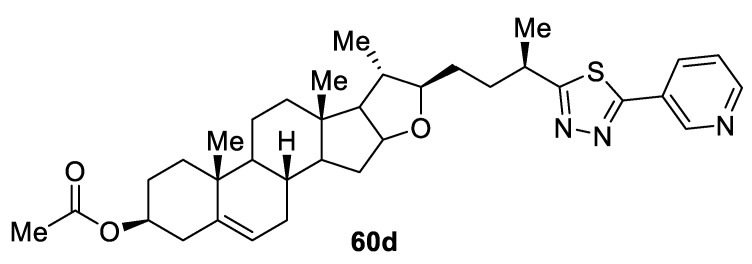
Structure of compound **60d**.

In the same year, two series of novel diosgenin derivatives bearing 1,3,4-oxadiazole or 1,3,4-thiadiazole moieties were evaluated for their cytotoxicity in four human cancer cell lines (HepG2, A549, MCF-7, and HCT-116) and normal human gastric epithelial cells (GES-1) using the MTT assay in vitro. The data revealed that the 1,3,4-thiadiazole series were more active than the 1,3,4-oxadiazole series against HepG2 and A549 cells. Among the 1,3,4-thiadiazoles, compound **60d** (Figure 43) with a 3-pyridyl group at the C5 position was the most potent. It was 6.7-fold more potent than the parent drug diosgenin (DG) against the A549 cell line (IC_50_ = 3.93 µM and 26.41 µM, respectively, as well as being 2.9-fold more potent than DG against the HepG2 cell line (MIC = 11.73 μM and 33.87 μM, respectively). Moreover, compound **60d** displayed low toxicity against non-cancer cells GES-1 cells (IC_50_ = 420.4 µM), showing high selectivity [58].

**Figure 44:**
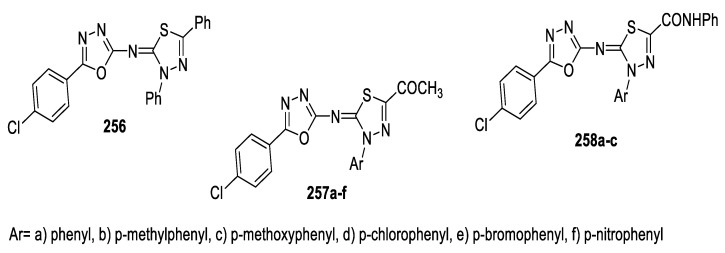
Structures of compounds **256**, **257a–f**, and **258a–c**.

On the basis that combining different heterocyclic nuclei may show synergistic effects, Kamal and Sobhy decided to merge the two bioisosteres, 1,3,4- oxadiazole and 1,3,4-thiadiazole, in one system to enhance the biological activity of the synthesized compounds and evaluated their cytotoxicity against a human colon carcinoma cell line (HCT-116) using Doxorubicin as a reference drug. Among all the tested compounds, the most active derivatives were **257d** and **258c** (IC_50_ = 0.73 and 0.86 μg/mL, respectively), while the other compounds showed moderate to poor activity compared to Doxorubicin IC_50_ = 0.42 μg/mL. The assay results and an examination of the structure–activity relationship revealed that, as a substituent at position 2 of 1,3,4-thiadiazole, the acetyl group (COCH_3_) (**257a–f**) resulted in higher activity than the anilide group (CONHPh) (**258a–c**), which was better than the phenyl moiety (Ph) **256**. Additionally, for substituents at position 4 of 1,3,4-thiadiazole, 4-ClC_6_H_4_ > 4-NO_2_C6H_4_ > 4-OCH_3_C_6_H_4_ > 4-CH_3_C_6_ H_4_ > 4-BrC_6_H_4_ > C_6_H_5_ (Figure 44) [107].

**Figure 45:**
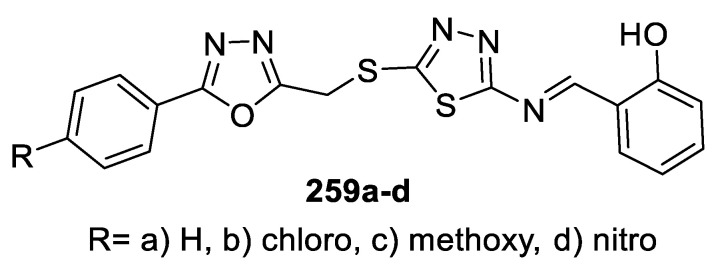
Structure of compounds **259a–d**.

Based on the same idea, a series of novel hybrid molecules containing 1,3,4-oxadiazole and 1,3,4-thiadiazole bearing the Schiff base moiety were designed and evaluated with respect to their in vitro antitumor activities against SMMC7721, MCF-7, and A549 human tumor cell lines by CCK-8 assay. The data revealed that some compounds were more potent than the positive control, 5-Fluorouracil (5-FU), against various cell lines. Among these compounds, compound **259b** (4-chloro) was the most potent against SMMC-7721 cells, with IC_50_ = 2.84 μM. Compounds **259c** (4-methoxy) and **259d** (4-nitro) displayed highly effective antitumor activities against MCF-7 cells, with IC_50_ = 4.56 and 4.25 μM, respectively. The unsubstituted phenyl derivative **259a** and the 4-nitro-substituted derivative **259d** showed significant activity against A549 cells, with IC_50_ = 4.11 and 4.13 μM, respectively. The pharmacological results suggest that the substituents of the phenyl ring on the 1,3,4-oxadiazole are important for modulating antiproliferative activities against various tumor cell lines (Figure 45) [108].

**Figure 46:**
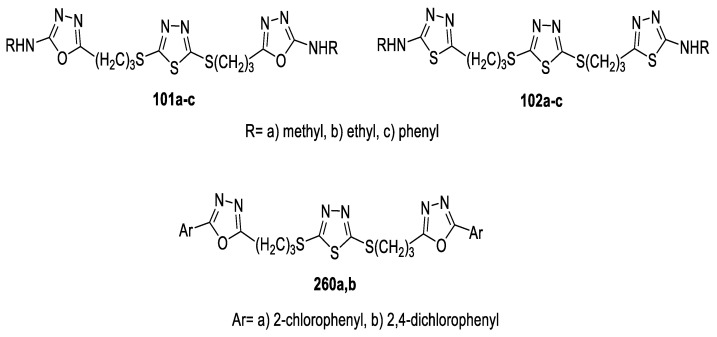
Structures of compounds **101a–c**, **102a–c** and **260a,b**.

In 2015, a series of 2,5-Bis[(2-substituted-1,3,4-oxa(thia)diazol-5-yl)propylthio]-1,3,4-thiadiazoles were tested for their in vitro antiproliferative activities in four different human cancer cell lines (human breast adenocarcinoma, MCF7, human ductal breast epithelial tumor, T47D, human epithelial colorectal adenocarcinoma, Caco-2, and human epithelial carcinoma, He La). All compounds demonstrated relatively high activities against the examined cell lines. There was no difference in activity related to the replacement of oxadiazole with a thiadiazole ring, while changing methyl and phenyl groups in (**101a,c**) and (**102a,c**) into ethyl groups **101b** and **102b** significantly enhanced the cytotoxic activity, with LD_50_ values ranging from 376 ng/µL to 438 ng/µL, which suggests a steric factor mediating either transport or molecular interaction of these compounds with cellular targets. Furthermore, the addition of one more Cl atom into the structure of compound **260a** (LD_50_ = 648–690 ng/µL) gave compound **260b**, with double the activity (LD_50_ = 356–398 ng/µL) (Figure 46) [76].

**Figure 47:**
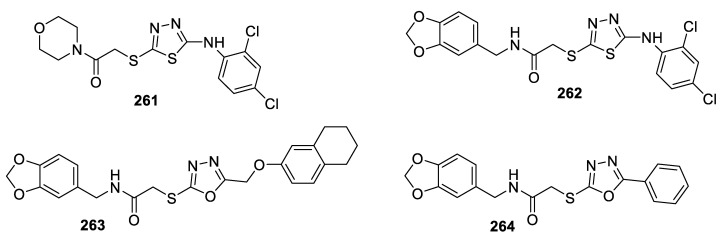
Structures of compounds **261**–**264**.

Ozdemir et al. designed and evaluated several oxadiazole and thiadiazole derivatives with respect to their anticancer effects on A549 human lung adenocarcinoma and C6 rat glioma cell lines and evaluated their inhibitory effects on matrix metalloproteinases (MMPs). Compounds **261**, **263**, and **264** showed high cytotoxic activity against the C6 cell line, with IC_50_ ranging from 0.0128 mM to 0.157 mM, compared to Cisplatin, with IC_50_ = 0.103 mM. Only the benzodioxole-substituted oxadiazoles **263** and **264** had a cytotoxic effect on the A549 cell line, with IC_50_ values of 0.125 and 0.349 mM, without causing any toxicity towards the NIH/3T3 mouse embryonic fibroblast cell line, which suggests that the (1,3-benzodioxol-5-ylmethyl) amino group enhances the antitumor activity against A549 cells. At the same time, the benzodioxole-substituted thiadiazole analog **262** was inactive against A549 cell lines, which proves that the oxadiazole scaffold is essential for activity against A549 cells. Compounds **263** and **264** were also the most effective MMP-9 inhibitors in this series. Moreover, docking studies pointed out that compounds **263** and **264** had a good affinity for the active site of the MMP-9 enzyme (Figure 47) [109].

**Figure 48:**
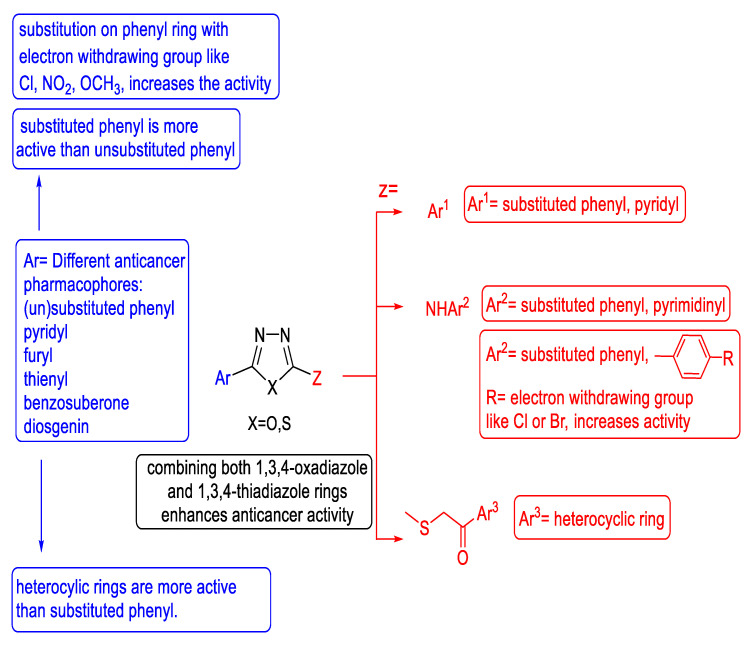
Collective anticancer SAR for substituted 1,3,4-oxa-/thiadiazoles.

The structure–activity relationship of the anticancer activity of substituted 1,3,4-oxa-/thiadiazoles is summarized in Figure 48.

### 3.4. Antidiabetic Activity

**Figure 49:**
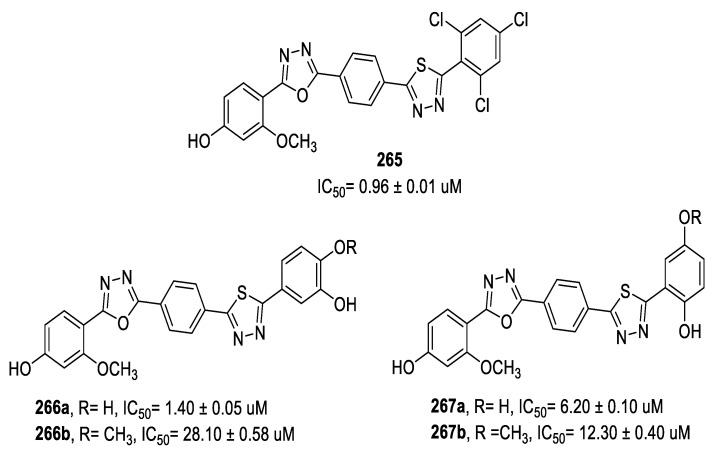
Structures of compounds **265**, **266a,b**, and **267a,b**.

In 2019, Taha et al., reported a series of 5-(4-hydroxy-2-methoxyphenyl)-1,3,4-oxadiazoles coupled to 5-aryl-1,3,4-thiadiazole motifs through a phenyl ring as potent β-glucuronidase inhibitors. Several analogs were more potent enzyme inhibitors (IC_50_ = 0.96–28.10 μM) than the standard D-saccharic acid 1,4-lactone (IC_50_ = 48.40 μM). Study of the structure–activity relationships indicated that the nature and relative position of substituents on the phenyl ring attached to thiadiazole greatly affected the inhibitory potency. The 2,4,6-trichlorophenyl derivative **265** displayed superior activity (IC_50_ = 0.96 μM) compared to monochloro-substituted analogs, regardless of the position (*o*,*m or p-*). Moreover, compound **266a** with the 3,4-dihydroxyphenyl moiety (IC_50_ = 1.40 μM) was four times more potent than the 2,5-dihydroxyphenyl analog **267a** (IC_50_ = 6.20 μM). A docking study revealed that the hydroxyl groups in 6 had higher potential to be engaged in proper hydrogen bonding interaction with the enzyme active site than in 7. In contrast, compound **267b** having 2-hydroxy-5-methoxy groups on the phenyl ring was much more potent than the 3-hydroxy-4-methoxy phenyl derivative **266b**, with IC_50_ = 12.30 and 28.10 μM, respectively. The steric effect of the methoxy group next to the hydroxy function in **266b** decreased the ability of the hydroxyl group to be involved in H-bonding with the enzyme and reduced the activity (Figure 49) [109].

### 3.5. Antioxidant Activity

**Figure 50:**
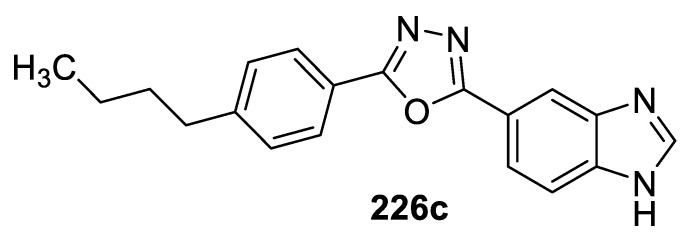
Structure of compound **226c**.

A series of nuclear factor erythroid 2-related factor 2 (Nrf2) activators with 1,3,4-(oxa)thiadiazole cores were designed as neuroprotective agents against oxidative stress. 2-(1*H*-benzo[d]imidazol-5-yl)-5-(4-butylphenyl)-1,3,4-oxadiazole **226c** (Figure 50) exhibited the highest cytoprotective and Nrf2 activation effects in a neuron-like PC-12 cells. It also showed good potential to penetrate the BBB and reach the CNS as a neuroprotective agent. Thus, compound **226c** represents a new chemotype against oxidative stress and provides a new therapeutic strategy for different neurodegenerative diseases, including Alzheimer’s disease. An SAR study on this series of Nrf2 activators revealed that the 1,3,4-oxadiazole derivatives showed higher Nrf2 inductivity than the 1,3,4-thiadiazole analogs as a result of their improved water solubility and lower lipophilicity [92].

In addition to the reported [99] antimicrobial activity of 2-(bis((1,3,4-oxadiazolyl/1,3,4-thiadiazolyl) methylthio)methylene)malononitrile derivatives (**241a–c**, **242a–c**) (Figure 31), the compounds were tested for the antioxidant property by NO and DPPH methods. The results revealed that only the compounds with an oxadiazole unit (**241a–c**) showed high antioxidant activity in both methods.

**Figure 51:**
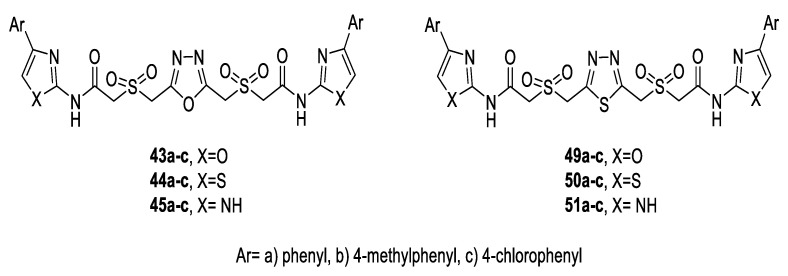
Structures of compounds **43a–c**–**45a–c** and **49a–c**–**51a–c**.

Nabhubygari et al. tested the antioxidant activity of a variety of symmetrical 2,4-bis-oxazolyl/thiazolyl/imidazolylacetamido-sulfonylmethyl 1,3,4-oxadiazoles (**43a–c**, **44a–c**, **45a–c**) and 1,3,4-thiadiazoles (**49a–c**, **50a–c**, **51a–c**) (Figure 51) using 2,2,-diphenyl-1-picrylhydrazyl (DPPH) and nitric oxide (NO) methods. The results showed that oxadiazole derivatives (**43a–c**, **44a–c**, **45a–c**) exhibited higher activity than the corresponding thiadiazoles (**49a–c**, **50a–c**, **51a–c**). Among the tested compounds, the bis-(4-methylphenyl oxazolyl)oxadiazole **43b** and bis-(4-methylphenyl oxazolyl) thiadiazole **49b** were the most potent antioxidants, and they demonstrated higher activity than the standard ascorbic acid in both methods. These results suggest that the presence of an electron-donating methyl substituent on the aromatic ring enhanced the antioxidant activity [51].

**Figure 52:**
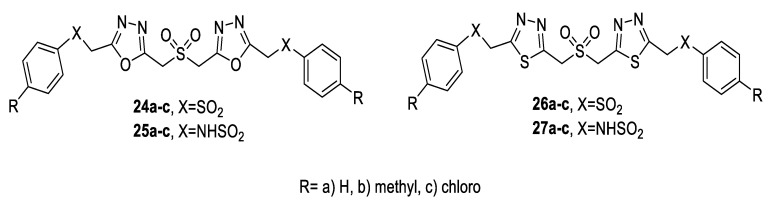
Structures of compounds **24a–c**–**27a–c**.

Similar results were observed upon evaluating the antioxidant potential of sulfone-/sulfonamide-linked bis(oxadiazoles) and bis(thiadiazoles) using the DPPH and NO methods. The 4-methylphenyl sulfone/sulfonamide-linked bis(oxadiazoles) **24b** and **25b** presented higher antioxidant activity than Ascorbic acid. The SAR of the tested compounds revealed that compounds with oxadiazole rings (**24a–c** and **25a–c**) showed higher radical scavenging activity than the corresponding thiadiazoles (**26a–c** and **27a–c**). It was also observed that sulfonamide-linked bis(oxadiazoles) and bis(thiadiazoles) (**25a–c** and **27a–c**) showed comparatively higher antioxidant activity than sulfone-linked bis(oxadiazoles) and bis(thiadiazoles) (**24a–c** and **26a–c**) (Figure 52) [2].

NO and DPPH scavenging activities were assayed for a series of 2-(arylaminosulfonyl methyl)-1,3,4-oxa(thia)diazole derivatives (**33-36**) (Figure 34). The results highlighted that the compounds with an oxadiazole unit (**33a–c**, **34a–c**) exhibited better antioxidant activity than did those with a thiadiazole unit (**35a–c**, **36a–c**), where compounds **33a**, **33c**, **34a** and **34c** showed high potency in both methods at a concentration of 100 μM [50].

**Figure 53:**
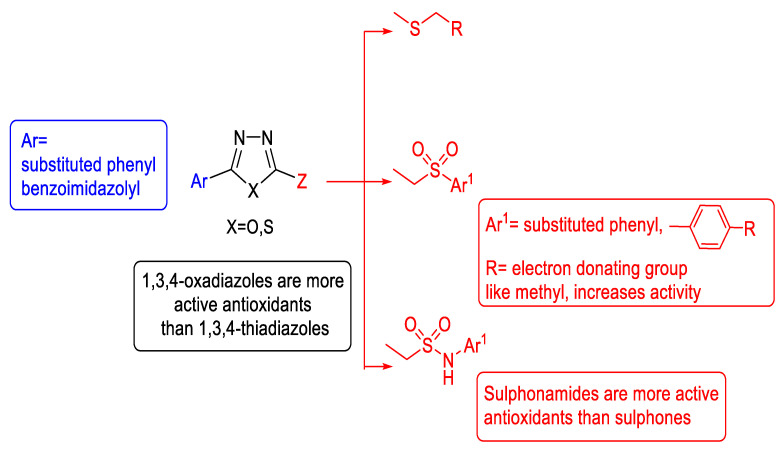
Collective antioxidant SAR for arylsulfide-, sulfone-, and sulfonamide-substituted 1,3,4-oxa-/thiadiazoles.

A summary of the antioxidant structure–activity relationship for substituted 1,3,4-oxa-/thiadiazoles is shown in Figure 53.

### 3.6. Analgesic and Anti-inflammatory Activities

**Figure 54:**
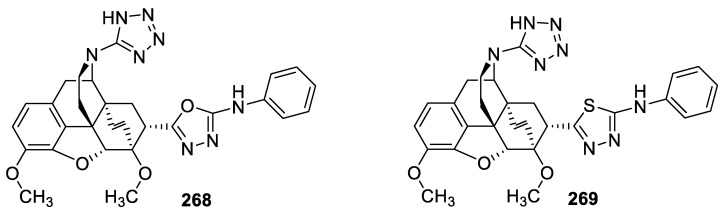
Structures of compounds **268** and **269**.

In 2013, some *N*-(tetrazol-1*H*-5-yl)-6,14-endoethenotetrahydrothebaine 7α-substituted 1,3,4-(oxa)thiadiazoles were prepared and screened for analgesic activity in rats, applying hot-plate and tail-flick tests and employing Morphine as a reference drug. At 0.5 and 1 h after administration, the phenylamino-oxadiazole derivative **268** (reaction time in hot plate test = 9.78 s, tail-flick test = 21.45 s) was found to be more potent than Morphine (reaction time in hot plate test = 6.28 s, tail-flick test = 15.90 s). Conversely, the thiadiazole analog **269** was moderately active. The results demonstrated that the oxadiazole group on the 7α position of thebaine increased the analgesic activity (Figure 54) [60].

**Figure 55:**
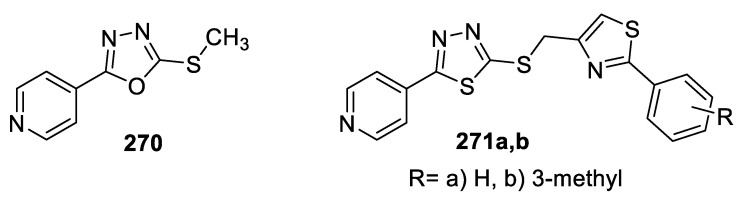
Structures of compounds **270** and **271a,b**.

A series of polyheterocyclic thioethers containing 1,3,4-(oxa)thiadiazoles were examined for anti-inflammatory activity using carrageenan-induced rat paw edema assay and Diclofenac as a reference drug. Compounds **270**, **271a**, and **271b** were more effective than Diclofenac in alleviating carrageenan-induced edema after 2, 3, and 4 h of treatment, with inhibition percentages of 37.5–55%, 30–35%, and 38–48%, respectively. In this study, oxadiazole and thiadiazole isosteres were presented as potent anti-inflammatory agents with no difference in their activities (Figure 55) [110].

## 4. Conclusions

This study is a review article summarizing most of the published work about the medicinally important pharmacophores 1,3,4-oxadiazoles and 1,3,4-thiadiazoles throughout the past 10 years. The review presents different synthetic approaches to the two bioisosteres using different starting compounds, comparing the differences in the reagents used, the conditions, and the yields of the two classes. This comparative study also identified their pharmacological activities, as well as introducing deduced collective structure–activity relationship charts for antimicrobial, anticancer, and antioxidant activities. In most cases, it was concluded that 1,3,4-thiadiazoles are more active antimicrobial agents than 1,3,4-oxadiazoles, in contrast to antioxidant activity, where 1,3,4-oxadiazoles are more active agents than 1,3,4-thiadiazoles. Concerning anticancer activity, it was noticed that combining the 1,3,4-oxadiazole ring with the 1,3,4-thiadiazole ring enhanced the activity.

## Data Availability

Not applicable.

## References

[1] Swapna M., Premakumari C., Reddy S.N., Padmaja A., Padmavathi V. (2013). Synthesis and antioxidant activity of a variety of sulfonamidomethane linked 1,3,4-oxadiazoles and thiadiazoles. Chem. Pharm. Bull..

[2] Padmaja A., Pedamalakondaiah D., Sravya G., Reddy G.M., Kumar M.V.J. (2015). Synthesis and antioxidant activity of a new class of sulfone/sulfonamide-linked Bis(Oxadiazoles), Bis(Thiadiazoles), and Bis(Triazoles). Med. Chem. Res..

[3] Meanwell N.A. (2011). Synopsis of some recent tactical application of bioisosteres in drug design. J. Med. Chem..

[4] Siddiqui S.M., Salahuddin A., Azam A. (2013). Synthesis of some 1,3,4-thiadiazole derivatives as inhibitors of entamoeba histolytica. Med. Chem. Res..

[5] Boström J., Hogner A., Llinàs A., Wellner E., Plowright A.T. (2012). Oxadiazoles in medicinal chemistry. J. Med. Chem..

[6] Madhu Sekhar M., Yamini G., Divya K.R.G., Padmavathi V., Padmaja A. (2019). Synthesis and bioassay of a new class of disubstituted 1,3,4-oxadiazoles, 1,3,4-thiadiazoles and 1,2,4-triazoles. Med. Chem. Res..

[7] Wang P.Y., Zhou L., Zhou J., Wu Z.B., Xue W., Song B.A., Yang S. (2016). Synthesis and antibacterial activity of pyridinium-tailored 2,5-substituted-1,3,4-oxadiazole thioether/sulfoxide/sulfone derivatives. Bioorg. Med. Chem. Lett..

[8] Hannoun M.H., Hagras M., Kotb A., El-Attar A.A.M.M., Abulkhair H.S. (2020). Synthesis and antibacterial evaluation of a novel library of 2-(Thiazol-5-Yl)-1,3,4-oxadiazole derivatives against Methicillin-Resistant Staphylococcus Aureus (MRSA). Bioorg. Chem..

[9] Karabanovich G., Němeček J., Valášková L., Carazo A., Konečná K., Stolaříková J., Hrabálek A., Pavliš O., Pávek P., Vávrová K. (2017). S-Substituted 3,5-dinitrophenyl 1,3,4-oxadiazole-2-thiols and tetrazole-5-thiols as highly efficient antitubercular agents. Eur. J. Med. Chem..

[10] Verma G., Chashoo G., Ali A., Khan M.F., Akhtar W., Ali I., Akhtar M., Alam M.M., Shaquiquzzaman M. (2018). Synthesis of pyrazole acrylic acid based oxadiazole and amide derivatives as antimalarial and anticancer agents. Bioorg. Chem..

[11] Almasirad A., Shafiee A., Abdollahi M., Noeparast A., Shahrokhinejad N., Vousooghi N., Tabatabai S.A., Khorasani R. (2011). Synthesis and analgesic activity of new 1,3,4-oxadiazoles and 1,2,4-triazoles. Med. Chem. Res..

[12] Nazar S., Siddiqui N., Alam O. (2020). Recent progress of 1,3,4-oxadiazoles as anticonvulsants: Future horizons. Arch. Pharm..

[13] Kumar Singh A., Lohani M., Parthsarthy R. (2013). Synthesis, characterization and anti-inflammatory activity of various isatin derivatives. Iran. J. Pharm. Res..

[14] El-Essawy F.A., El-Sayed W.A., El-Kafrawy S.A., Morshedy A.S., Abdel-Rahman A.H. (2008). Anti-hepatitis B virus activity of new 1,2,4-triazol-2-Yl- and 1,3,4-oxadiazol-2-Yl-2-pyridinone derivatives. Z. Naturforsch. Sect. C J. Biosci..

[15] El-Masry R.M., Al-Karmalawy A.A., Alnajjar R., Mahmoud S.H., Mostafa A., Kadry H.H., Abou-Seri S.M., Taher A.T. (2022). Newly synthesized series of oxoindole–oxadiazole conjugates as potential anti-SARS-CoV-2 agents: In silico and in vitro studies. New J. Chem..

[16] Ahsan M.J., Choupra A., Sharma R.K., Jadav S.S., Padmaja P., Hassan M.Z., Al-Tamimi A.B.S., Geesi M.H., Bakht M.A. (2017). Rationale design, synthesis, cytotoxicity evaluation, and molecular docking studies of 1,3,4-oxadiazole analogues. Anticancer Agents Med. Chem..

[17] Hamdi N., Passarelli V., Romerosa A. (2011). Synthesis, spectroscopy and electrochemistry of new 4-(4-acetyl-5-substituted-4,5-dihydro-1,3,4-oxodiazol-2-yl)methoxy)-2H-chromen-2-ones as a novel class of potential antibacterial and antioxidant derivatives. Comptes Rendus Chim..

[18] Siwach A., Verma P.K. (2020). Therapeutic potential of oxadiazole or furadiazole containing compounds. BMC Chem..

[19] Vaidya A., Pathak D., Shah K. (2021). 1,3,4-Oxadiazole and its derivatives: A review on recent progress in anticancer activities. Chem. Biol. Drug Des..

[20] Pouliot M., Angers L., Hamel J. (2012). Synthesis of 1,3,4-oxadiazoles from 1,2-diacylhydrazines using [Et2NSF2]BF4 as a practical cyclodehydration agent. Org. Biomol. Chem..

[21] Ogata M., Atobe H., Kushida H. (1971). In vitro sensitivity of mycoplasmas isolated from various animals and sewage to antibiotics and nitrofurans. J. Antibiot..

[22] Schlecker R., Thieme P. (1988). The synthesis of antihypertensive 3-(1,3,4-oxadiazol-2-Yl)phenoxypropanolahines. Tetrahedron.

[23] Serrao E., Odde S., Ramkumar K., Neamati N. (2009). Raltegravir, elvitegravir, and metoogravir: The birth of “Me-Too” HIV-1 integrase inhibitors. Retrovirology.

[24] Partyka R.A., Crenshaw R.R. (1977). 1,3,4-Oxadiazole Amide 1977.

[25] Vardan S., Smulyan H., Mookherjee S., Eich R. (1983). Effects of tiodazosin, a new antihypertensive, hemodynamics and clinical variables. Clin. Pharmacol. Ther..

[26] Brandenberger H., Maes R., Curtius H.C., Roth M. (1997). Analytical toxicity for clinical, forensic and pharmaceutical chemists. Clinical Biochemistry.

[27] Yarmohammadi E., Beyzaei H., Aryan R., Moradi A. (2021). Ultrasound-assisted, low-solvent and acid/base-free synthesis of 5-substituted 1,3,4-oxadiazole-2-thiols as potent antimicrobial and antioxidant agents. Mol. Divers..

[28] Haque S.U., Dashwood M.R., Heetun M., Shiwen X., Farooqui N., Ramesh B., Welch H., Savage F.J., Ogunbiyi O., Abraham D.J. (2013). Efficacy of the specific endothelin a receptor antagonist Zibotentan (ZD4054) in colorectal cancer: A preclinical study. Mol. Cancer Ther..

[29] Glomb T., Szymankiewicz K., Świątek P. (2018). Anti-cancer activity of derivatives of 1,3,4-oxadiazole. Molecules.

[30] Johansson A., Löfberg C., Antonsson M., Von Unge S., Hayes M.A., Judkins R., Ploj K., Benthem L., Lindén D., Brodin P. (2016). Discovery of (3-(4-(2-Oxa-6-Azaspiro[3.3]Heptan-6- Ylmethyl)Phenoxy)Azetidin-1-Yl)(5-(4-Methoxyphenyl)-1,3,4-Oxadiazol-2- Yl)Methanone (AZD1979), a melanin concentrating hormone receptor 1 (MCHr1) antagonist with favourable physicochemical properties. J. Med. Chem..

[31] Li Y., Geng J., Liu Y., Yu S., Zhao G. (2013). Thiadiazole-a promising structure in medicinal chemistry. ChemMedChem.

[32] Yan L., Deng M., Chen A., Li Y., Zhang W., Du Z.Y., Dong C.Z., Meunier B., Chen H. (2019). Synthesis of N-pyrimidin[1,3,4]oxadiazoles and N-Pyrimidin[1,3,4]-thiadiazoles from 1,3,4-oxadiazol-2-amines and 1,3,4-thiadiazol-2-amines via Pd-catalyzed heteroarylamination. Tetrahedron Lett..

[33] Gomha S.M., Kheder N.A., Abdelhamid A.O., Mabkhot Y.N. (2016). One pot single step synthesis and biological evaluation of some novel Bis(1,3,4-Thiadiazole) derivatives as potential cytotoxic agents. Molecules.

[34] Hamama W.S., Gouda M.A., Badr M.H., Zoorob H.H. (2013). Synthesis, antioxidant, and antitumor evaluation of certain new n-substituted-2-amino-1,3,4-thiadiazoles. Med. Chem. Res..

[35] Chandrakantha B., Isloor A.M., Shetty P., Fun H.K., Hegde G. (2014). Synthesis and biological evaluation of novel substituted 1,3,4-thiadiazole and 2,6-di aryl substituted imidazo [2,1-b] [1,3,4] thiadiazole derivatives. Eur. J. Med. Chem..

[36] Khan I., Tantray M.A., Hamid H., Alam M.S., Kalam A., Dhulap A. (2016). Synthesis of benzimidazole based thiadiazole and carbohydrazide conjugates as glycogen synthase kinase-3β inhibitors with anti-depressant activity. Bioorg. Med. Chem. Lett..

[37] Siddiqui N., Ahuja P., Malik S., Arya S.K. (2013). Design of benzothiazole-1,3,4-thiadiazole conjugates: Synthesis and anticonvulsant evaluation. Arch. Pharm..

[38] Shkair M.H.A., Shakya A.K., Raghavendra N.M., Naik R.R. (2016). Molecular modeling, synthesis and pharmacological evaluation of 1,3,4- thiadiazoles as anti-inflammatory and analgesic agents. Med. Chem..

[39] Hill J., Katritzky A.R., Rees C.W. (1984). 4.23—1,3,4-oxadiazoles. Comprehensive Heterocyclic Chemistry.

[40] Kakitani T., Kakitani H. (1977). Application of self-consistent HMO theory to heteroconjugated molecules. Theor. Chim. Acta.

[41] Ha T.-K. (1979). A Theoretical study of the electronic structure and properties of some five-membered heterocyclic compounds: Pyrazole, imidazole, furan, isoxazole, 1,2,5-oxadiazole and 1,3,4-oxadiazole. J. Mol. Struct..

[42] Kosobutskii V.A., Kagan G.I., Betyakov V.K. (1972). Amide-imidol tautomerism in aromatic polyamides. J. Struct. Chem..

[43] Aydogan F., Turgut Z., Ocal N. (2002). Synthesis and electronic structure of new aryl- and alkyl-substituted 1,3,4-oxadiazole-2-thione derivatives. Turk. J. Chem..

[44] Khiati Z., Othman A.A., Guessas B. (2007). Synthesis and antibacterial activity of 1,3,4-oxadiazole and 1,2,4-triazole derivatives of salicylic acid and its synthetic intermediates. S. Afr. J. Chem..

[45] Sharma B., Verma A., Prajapati S., Sharma U.K. (2013). Synthetic methods, chemistry, and the anticonvulsant activity of thiadiazoles. Int. J. Med. Chem..

[46] Goerdler J., Ohm J., Tegmeyer O. (1956). Darstellung und eigenschaften des 1.2.4- und des 1.3.4-thiodiazols. Chem. Ber..

[47] Othman A.A., Kihel M., Amara S. (2019). 1,3,4-oxadiazole, 1,3,4-thiadiazole and 1,2,4-triazole derivatives as potential antibacterial agents. Arab. J. Chem..

[48] Strzemecka L., Maciejewska D., Urbanczyk Z. (2003). The structure of N-allyl derivatives of (5-(2′-pyridyl)- [1,3,4]thiadiazol-2-yl) amine in solution and the solid state studied by the 1H, 13C, 15N NMR spectroscopy, X-ray crystallography and DFT computations. J. Mol. Struct..

[49] Padmaja A., Muralikrishna A., Rajasekhar C., Padmavathi V. (2011). Synthesis and antimicrobial activity of pyrrolyl/pyrazolyl arylaminosulfonylmethyl 1,3,4-oxadiazoles, 1,3,4-thiadiazoles and 1,2,4-triazoles. Chem. Pharm. Bull..

[50] Padmavathi V., Reddy S.N., Reddy G.D., Padmaja A. (2010). Synthesis and bioassay of aminosulfonyl-1,3,4-oxadiazoles and their interconversion to 1,3,4-thiadiazoles. Eur. J. Med. Chem..

[51] Basha N.M., Reddy P.R., Padmaja A., Padmavathi V., Duszynska B., Bojarski A.J., Strekowski L. (2016). Synthesis and antioxidant activity of bis-oxazolyl/thiazolyl/imidazolyl 1,3,4-oxadiazoles and 1,3,4-thiadiazoles. J. Heterocycl. Chem..

[52] Du Y., Wan Z., Chen L., Wu L. (2019). One pot solvent-free solid state synthesis, photophysical properties and crystal structure of substituted azole derivatives. J. Mol. Struct..

[53] Anna K., Kudelko A., Marcin Ś., Kruszynski R. (2020). Microwave-promoted synthesis of highly luminescent s-tetrazine-1,3,4- oxadiazole and s-tetrazine-1,3,4-thiadiazole hybrids. Dye. Pigment..

[54] Nagendra G., Lamani R.S., Narendra N., Sureshbabu V.V. (2010). A Convenient synthesis of 1,3,4-thiadiazole and 1,3,4-oxadiazole based peptidomimetics employing diacylhydrazines derived from amino acids. Tetrahedron Lett..

[55] Mutchu B.R., Kotra V., Onteddu S.R., Boddapati S.N.M., Bollikolla H.B. (2019). Synthesis, cytotoxicity and antimicrobial evaluation of some new 2-aryl,5-substituted 1,3,4-oxadiazoles and 1,3,4-thiadiazoles. Chem. Africa.

[56] Taylor P., Prajapati A.K., Modi V. (2011). Mesogenic bent-shaped nitrooxadiazoles and thiadiazoles. Liq. Cryst..

[57] Schmidt M. (2013). A New Late-Stage Lawesson’s Cyclization Strategy Towards the Synthesis of Aryl 1,3,4-Thiadiazole-2-Carboxylate Esters.

[58] Zhang J., Wang X., Yang J., Guo L., Wang X., Song B., Wang W. (2019). Novel diosgenin derivatives containing 1,3,4-oxadiazole/thiadiazole moieties as potential antitumor agents: Design, synthesis and cytotoxic evaluation. Eur. J. Med. Chem..

[59] Hajimahdi Z., Zarghi A., Zabihollahi R., Aghasadeghi M.R. (2012). Synthesis, biological evaluation, and molecular modeling studies of new 1,3,4-oxadiazole- and 1,3,4-thiadiazole-substituted 4-oxo-4h-pyrido[1,2-a]pyrimidines as anti-HIV-1 agents. Med. Chem. Res..

[60] Yavuz S., Unal Y., Pamir O., Yılmazer D., Kurtipek O., Kavutcu M., Arslan M., Ark M., Yıldırır Y. (2013). Synthesis and pharmacological evaluation of some novel thebaine derivatives: *N*-(tetrazol-1*H*-5-yl)-6,14- endoethenotetrahydrothebaine incorporating the 1,3,4-oxadiazole or the 1,3,4-thiadiazole moiety. Arch. Pharm. Chem. Life Sci..

[61] Ali A.A., Soliman M.A., Aouad M.R., Messali M., Ali A.A., Soliman M.A., Aouad M.R. (2019). Synthesis, characterization, and antimicrobial screening of novel 1,2,4-triazoles, 1,3,4- thiadiazoles, and 1,3,4-oxadiazoles bearing the indole moiety. Org. Prep. Proced. Int..

[62] Abu-Hashem A.A. (2021). Synthesis and antimicrobial activity of new 1,2,4-triazole, 1,3,4-oxadiazole, 1,3,4-thiadiazole, thiopyrane, thiazolidinone, and azepine derivatives. J. Heterocycl. Chem..

[63] Yang S., Choe J., Abdildinova A., Gong Y. (2015). A Highly efficient diversification of 2-amino/amido-1,3,4-oxadiazole and 1,3,4-thiadiazole derivatives via reagent-based cyclization of thiosemicarbazide intermediate on solid-phase. ACS Comb. Sci..

[64] Farshori N.N., Banday M.R., Ahmad A., Khan A.U., Rauf A. (2010). Synthesis, characterization, and in vitro antimicrobial activities of 5-alkenyl/hydroxyalkenyl-2-phenylamine-1,3,4-oxadiazoles and thiadiazoles. Bioorg. Med. Chem. Lett..

[65] Coruh I., Rollas S., Turan S. (2012). Synthesis and evaluation of cytotoxic activities of some 1,4-disubstituted thiosemicarbazides, 2,5-disubstituted-1,3,4- thiadiazoles and 1,2,4-triazole-5-thiones derived from benzilic acid hydrazide. Marmara Pharm. J..

[66] Burbuliene M., Simkus A., Vainilavicius P. (2012). Synthesis of new pyrimidinylthio-substituted 1,3,4-oxa(thia)diazoles and 1,2,4-triazoles. J. Sulfur Chem..

[67] Szulczyk D., Tomaszewski P., Michał J., Kozioł A.E., Lis T., Collu D., Iuliano F., Struga M. (2017). Synthesis and biological activities of ethyl 2-(2-pyridylacetate) derivatives containing thiourea, 1,2,4-triazole, thiadiazole and oxadiazole moieties. Molecules.

[68] Deshmukh R., Karale B., Akolkar H., Randhavane P. (2017). Synthesis and antibacterial screening of 1,3,4-thiadiazoles, 1,2,4-triazoles, and 1,3,4-oxadiazoles containing piperazine nucleus. J. Heterocycl. Chem..

[69] Abdo N.Y.M., Kamel M.M. (2015). Synthesis and anticancer evaluation of 1,3,4-oxadiazoles, 1,3,4-thiadiazoles, 1,2,4-triazoles and mannich bases. Chem. Pharm. Bull..

[70] Reddy K.R., Mamatha R., Babu M.S.S., Kumar K.S., Jayaveera K.N., Narayanaswamy G. (2013). Synthesis and antimicrobial activities of some triazole, thiadiazole, and oxadiazole substituted coumarins. J. Hetercyclic Chem..

[71] Abdel-fattah H.A., El-Etrawy A.S., Gabr N.M.R. (2014). Synthesis and biological evaluation of some new 1,3,4-oxa, thiadiazole and 1,2,4-triazole derivatives attached to benzimidazole. Int. J. Pharm. Chem..

[72] El-essawy F.A., El-sayed W.A. (2013). Synthesis of new 1,3,4-oxadiazol, thiadiazole, 1,2,4-triazole, and arylidene hydrazide derivatives of 4-oxo-1,4-dihydroquinoline with antimicrobial evaluation. J. Heterocycl. Chem..

[73] Andrews B., Ahmed M. (2013). Novel synthesis and characterization of some pyrimidine derivatives of oxadiazoles, triazole and 1,3,4-thiadiazoles. Asian J. Chem..

[74] Kalhor M., Dadras A. (2013). Synthesis, characterization, and herbicidal activities of new 1,3,4-oxadiazoles, 1,3,4-thiadiazoles, and 1,2,4-triazoles derivatives bearing (r)-5-chloro-3-fluoro-2-phenoxypyridin. J. Heterocycl. Chem..

[75] Zoumpoulakis P., Camoutsis C., Pairas G., Sokovic M., Glamoclija J., Potamitis C., Pitsas A. (2012). Synthesis of novel sulfonamide-1,2,4-triazoles, 1,3,4-thiadiazoles and 1,3,4-oxadiazoles, as potential antibacterial and antifungal agents. Biological evaluation and conformational analysis studies. Bioorg. Med. Chem..

[76] Rezki N., Al-Yahyawi A.M., Bardaweel S.K., Al-Blewi F.F., Aouad M.R. (2015). Synthesis of novel 2,5-disubstituted-1,3,4-thiadiazoles clubbed 1,2,4-triazole, 1,3,4-thiadiazole, 1,3,4-oxadiazole and/or schiff base as potential antimicrobial and antiproliferative agents. Molecules.

[77] Upadhyay P.K., Mirsha P. (2015). Design, synthesis and antifungal evaluation of novel substituted 1,3,4-oxadiazoles, and 1,3,4-thiadiazoles. Int. J. Pharm. Pharm. Sci..

[78] Barbuceanu S., Ilies D.C., Radulescu V., Socea L., Draghici C., Saramet G. (2014). Synthesis, Characterization and antioxidant activity evaluation of some 1,3,4-thiadiazole and 1,3,4-oxadiazole compounds. Rev. Chim..

[79] Barbuceanu S., Draghici C., Socea L., Enache C., Andreescu C., Saramet G. (2015). Hydrazinecarbothioamides and 1,3,4-thia/oxadiazoles derivatives with potential biological activity synthesis and spectral characterization. Rev. Chim..

[80] El-Essawy F.A., Rady S.I.M. (2011). Synthesis of some N-alkylated 1,2,4-triazoles, 1,3,4-oxadiazoles, and 1,3,4-thiadiazoles based on n-(furann-2-yl- methylidene)-4,6-dimethyl-1h-pyrazolo- [3,4-b]pyridine-3-amine. Chem. Heterocycl. Compd..

[81] Ebrahimi S. (2010). Synthesis of some pyridyl and cyclohexyl substituted 1,2,4 triazole, 1,3,4-thiadiazole and 1,3,4-oxadiazole derivatives. Eur. J. Chem..

[82] Yang S., Lee S., Kwak H., Gong Y. (2013). Regioselective synthesis of 2-amino-substituted 1,3,4-oxadiazole and 1,3,4-thiadiazole derivatives via reagent-based cyclization of thiosemicarbazide intermediate. J. Org. Chem..

[83] Niu P., Kang J., Tian X., Song L., Liu H., Wu J., Yu W., Chang J. (2015). Synthesis of 2-amino-1,3,4-oxadiazoles and 2-amino-1,3,4-thiadiazoles via sequential condensation and i2-mediated oxidative c-o/c-s bond formation. J. Org. Chem..

[84] Renuka N., Vivek H.K., Pavithra G., Ajay Kumar K. (2017). Synthesis of coumarin appended pyrazolyl-1,3,4-oxadiazoles and pyrazolyl-1,3,4-thiadiazoles: Evaluation of their in vitro antimicrobial and antioxidant activities and molecular docking studies. Russ. J. Bioorganic Chem..

[85] Aksenov N.A., Arutiunov N.A., Kirillov N.K., Aksenov D.A., Aksenov A.V., Rubin M. (2020). Preparation of 1,3,4-oxadiazoles and 1,3,4-thiadiazoles via chemoselective cyclocondensation of electrophilically activated nitroalkanes to (thio)semicarbazides or thiohydrazides. Chem. Heterocycl. Compd..

[86] Kumar S., Srivastava D.P. (2010). Efficient electrochemical synthesis, antimicrobial and antiinflammatory activity of 2–amino-5-substituted- 1,3,4-oxadiazole derivatives. Indian J. Pharm. Sci..

[87] Tresse C., Radigue R., Gomes Von Borowski R., Thepaut M., Hanh Le H., Demay F., Georgeault S., Dhalluin A., Trautwetter A., Ermel G. (2019). Synthesis and evaluation of 1,3,4-oxadiazole derivatives for development as broad-spectrum antibiotics. Bioorg. Med. Chem..

[88] Arafa W.A.A., Abdel-Magied A.F. (2017). Utilization of ultrasonic irradiation as green and effective one-pot protocol to prepare a novel series of bis-2-amino-1,3,4-oxa(thia)diazoles and bis-tetrazoles. Arkivoc.

[89] Gurunanjappa P., Kariyappa A.K. (2016). Design, synthesis and biological evaluation of 1,3,4-oxadiazoles/thiadiazoles bearing pyrazole scaffold as antimicrobial and antioxidant candidates. Curr. Chem. Lett..

[90] Szocs B., Tóth M., Docsa T., Gergely P., Somsák L. (2013). Synthesis of 2-(β-d-glucopyranosyl)-5-(substituted-amino)-1,3,4-oxa- and -thiadiazoles for the inhibition of glycogen phosphorylase. Carbohydr. Res..

[91] Lungu L., Ciocarlan A., Smigon C., Ozer I., Shova S., Gutu I., Vornicu N., Mangalagiu I., D’Ambrosio M., Aricu A. (2020). Synthesis and evaluation of biological activity of homodrimane sesquiterpenoids bearing 1,3,4-oxadiazole or 1,3,4-thiadiazole units. Chem. Heterocycl. Compd..

[92] Lin H., Qiao Y., Yang H., Li Q., Chen Y., Qu W., Liu W., Feng F., Sun H. (2020). Design and evaluation of Nrf2 activators with 1,3,4-oxa/thiadiazole core as neuro-protective agents against oxidative stress in PC-12 cells. Bioorg. Med. Chem. Lett..

[93] Aggarwal N., Kumar R., Dureja P., Khurana J.M. (2012). Synthesis of novel nalidixic acid-based 1,3,4-thiadiazole and 1,3,4-oxadiazole derivatives as potent antibacterial agents. Chem. Biol. Drug Des..

[94] Zhang L., Yu Y., Tang Q., Yuan J., Ran D., Tian B., Pan T., Gan Z. (2020). TiCl4 mediated facile synthesis of 1,3,4-oxadiazoles and 1,3,4-thiadiazoles. Synth. Commun..

[95] Fan X., Jiang X., Zhang Y., Chen Z., Zhu Y. (2015). Palladium-catalyzed one-pot synthesis of diazoles via tert-butyl isocyanide insertion. Org. Biomol. Chem..

[96] Dekhane D.V., Pawar S.S., Gupta S., Shingare M.S., Patil C.R., Thore S.N. (2011). Synthesis and anti-inflammatory activity of some new 4,5-dihydro-1,5-diaryl-1h-pyrazole-3-substituted-heteroazole derivatives. Bioorg. Med. Chem. Lett..

[97] Knyazyan A., Eliazyan K., Pivazyan V., Ghazaryan E., Harutyunyan S., Yengoyan A. (2012). Synthesis and growth regulatory activity of novel 5-(3-alkyl-4-methyl-2-thioxo-2,3-dihydro-thiazol-5-Yl)-3H-[1,3,4] thiadiazole(oxadiazole)-2-thiones and their derivatives. Heterocycl. Commun..

[98] Yadagiri B., Gurrala S., Bantu R., Nagarapu L., Polepalli S., Srujana G., Jain N. (2015). Synthesis and evaluation of benzosuberone embedded with 1,3,4-oxadiazole, 1,3,4-thiadiazole and 1,2,4-triazole moieties as new potential anti proliferative agents. Bioorg. Med. Chem. Lett..

[99] Padmavathi V., Reddy G.D., Reddy S.N., Mahesh K. (2011). Synthesis and biological activity of 2-(bis((1,3,4-oxadiazolyl/1,3,4-thiadiazolyl) methylthio)methylene)malononitriles. Eur. J. Med. Chem..

[100] Ramadoss N.S., Alumasa J.N., Cheng L., Wang Y., Li S., Chambers B.S., Chang H., Chatterjee A.K., Brinker A., Engels I.H. (2013). Small molecule inhibitors of trans-translation have broad-spectrum antibiotic activity. Proc. Natl. Acad. Sci. USA.

[101] Karabanovich G., Zemanová J., Smutný T., Székely R., Šarkan M., Centárová I., Vocat A., Pávková I., Čonka P., Němeček J. (2016). Development of 3,5-dinitrobenzylsulfanyl-1,3,4-oxadiazoles and thiadiazoles as selective antitubercular agents active against replicating and nonreplicating mycobacterium tuberculosis. J. Med. Chem..

[102] Al-Azzawi A.M., Hamd A.S. (2013). Synthesis, characterization and antimicrobial activity evaluation of new cyclic iimides containing 1,3,4-thiadiazole and 1,3,4- oxadiazole moieties. Int. J. Res. Pharm. Chem..

[103] Maddila S., Jonnalagadda S.B. (2013). Synthesis and antimicrobial activity of new 1,3,4-thiadiazoles containing oxadiazole, thiadiazole and triazole nuclei. Pharm. Chem. J..

[104] Madhu Sekhar M., Nagarjuna U., Padmavathi V., Padmaja A., Reddy N.V., Vijaya T. (2018). Synthesis and antimicrobial activity of pyrimidinyl 1,3,4-oxadiazoles, 1,3,4-thiadiazoles and 1,2,4-triazoles. Eur. J. Med. Chem..

[105] Xu W.M., Li S.Z., He M., Yang S., Li X.Y., Li P. (2013). Synthesis and bioactivities of novel thioether/sulfone derivatives containing 1,2,3-thiadiazole and 1,3,4-oxadiazole/thiadiazole moiety. Bioorg. Med. Chem. Lett..

[106] Rajak H., Agarawal A., Parmar P., Thakur B.S., Veerasamy R., Sharma P.C., Kharya M.D. (2011). 2,5-disubstituted-1,3,4-oxadiazoles/thiadiazole as surface recognition moiety: Design and synthesis of novel hydroxamic acid based histone deacetylase inhibitors. Bioorg. Med. Chem. Lett..

[107] Dawood K.M., Gomha S.M. (2015). Synthesis and anti-cancer activity of 1,3,4-thiadiazole and 1,3-thiazole derivatives having 1,3,4-oxadiazole moiety. J. Heterocycl. Chem..

[108] Zhang K., Wang P., Xuan L.N., Fu X.Y., Jing F., Li S., Liu Y.M., Chen B.Q. (2014). Synthesis and antitumor activities of novel hybrid molecules containing 1,3,4-oxadiazole and 1,3,4-thiadiazole bearing schiff base moiety. Bioorg. Med. Chem. Lett..

[109] Özdemir A., Sever B., Altıntop M.D., Temel H.E., Atlı Ö., Baysal M., Demirci F. (2017). Synthesis and evaluation of new oxadiazole, thiadiazole, and triazole derivatives as potential anticancer agents targeting MMP-9. Molecules.

[110] Toma A., Hapău D., Vlase L., Mogoşan C., Zaharia V. (2013). Synthesis and anti-inflammatory activity of 5-(pyridin-4-yl)-1,3,4-oxadiazole-2-thiol, 5-(pyridin-4-yl)-1,3,4-thiadiazole-2-thiol and 5-(pyridin-4-yl)-1,2,4-triazole-3-thiol derivatives. Clujul Med..

